# Rhodium-Catalyzed Rearrangement Reaction of Azabicyclo[4.1.0]heptenes bearing Cyclopropyl and Aryl Groups to Arylhexahydroisoquinolines

**DOI:** 10.1002/open.201200022

**Published:** 2012-07-16

**Authors:** Sori Son, Sun Young Kim, Young Keun Chung

**Affiliations:** a Intelligent Textile System Research Center and Department of Chemistry, College of Natural Sciences, Seoul National UniversitySeoul 151-747 (Korea) E-mail: ykchung@snu.ac.kr

**Keywords:** bicyclo[4.1.0]heptenes, catalysis, hexahydroisoquinolines, rearrangements, rhodium

Recently, the transition metal-catalyzed cycloisomerization has attracted much attention,^1^ because it can provide useful cyclic compounds from readily available starting materials and produce different cyclic compounds from the same substrate, depending on the catalyst and reaction conditions. One of the most studied substrates are 1,6-enynes.^2^ The transition metal-catalyzed cycloisomerization of 1,6-enynes can also lead to a variety of different reaction products.^3^ Among them, we have been especially interested in derivatives of bicyclo[4.1.0]heptene because we want to use the activity of the cyclopropanes in further reactions.^4^ In particular, when enynes, having a cyclopropyl substituent, were used as substrates, cyclic compounds bearing bis-cyclopropyl groups were obtained easily as major products (**B** and **B’**, Scheme 1).4f When the bis-cyclopropyl group was used as two three-carbon donors in the presence of a rhodium catalyst under carbon monoxide, bicyclic heptenones were isolated in reasonable to high yields (Scheme 1).4g

**Scheme 1:**
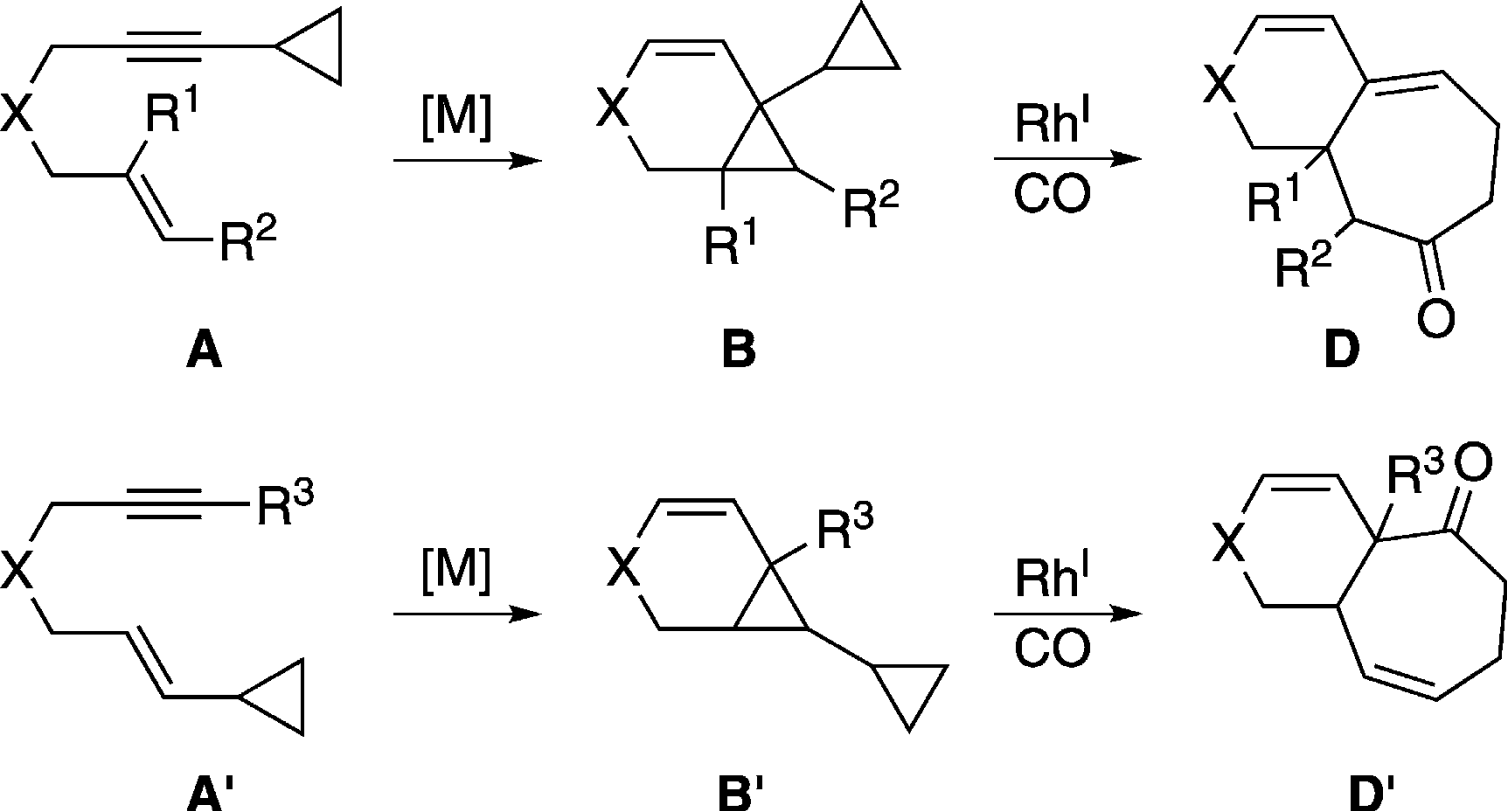
Transition metal-catalyzed cyclopropanation and carbonylative cycloaddition reactions.

Interestingly, the reaction pathway of the rhodium-catalyzed transformation of bicyclo[4.1.0]heptenes bearing a cyclopropyl group in the 7-position was highly dependent on the substituent in the 6-position (**B’**, Scheme 2).4h When the substituent was an aryl, a bicyclic diene (**E**) was obtained as a major product. However, when the substituent in the 6-position was an alkyl, a monocyclic triene (**F**) was isolated as a major product. Thus, the substituent on the substrate strongly influences the structure of the reaction product. These observations urged us to study a transformation of bicyclo[4.1.0]heptenes (**B**) bearing aryl and cyclopropyl groups in 1- and 6-positions, respectively, which had no reactivity in the [RhCl(COD)]_2_-catalyzed (cod=1,5-cyclooctadiene) carbonylative [3+3+1] cycloaddition reaction.4f However, we recently found that they are active in the presence of cationic rhodium compounds giving a new cyclization product, arylhexahydroisoquinoline. We herein communicate our new findings.

**Scheme 2:**
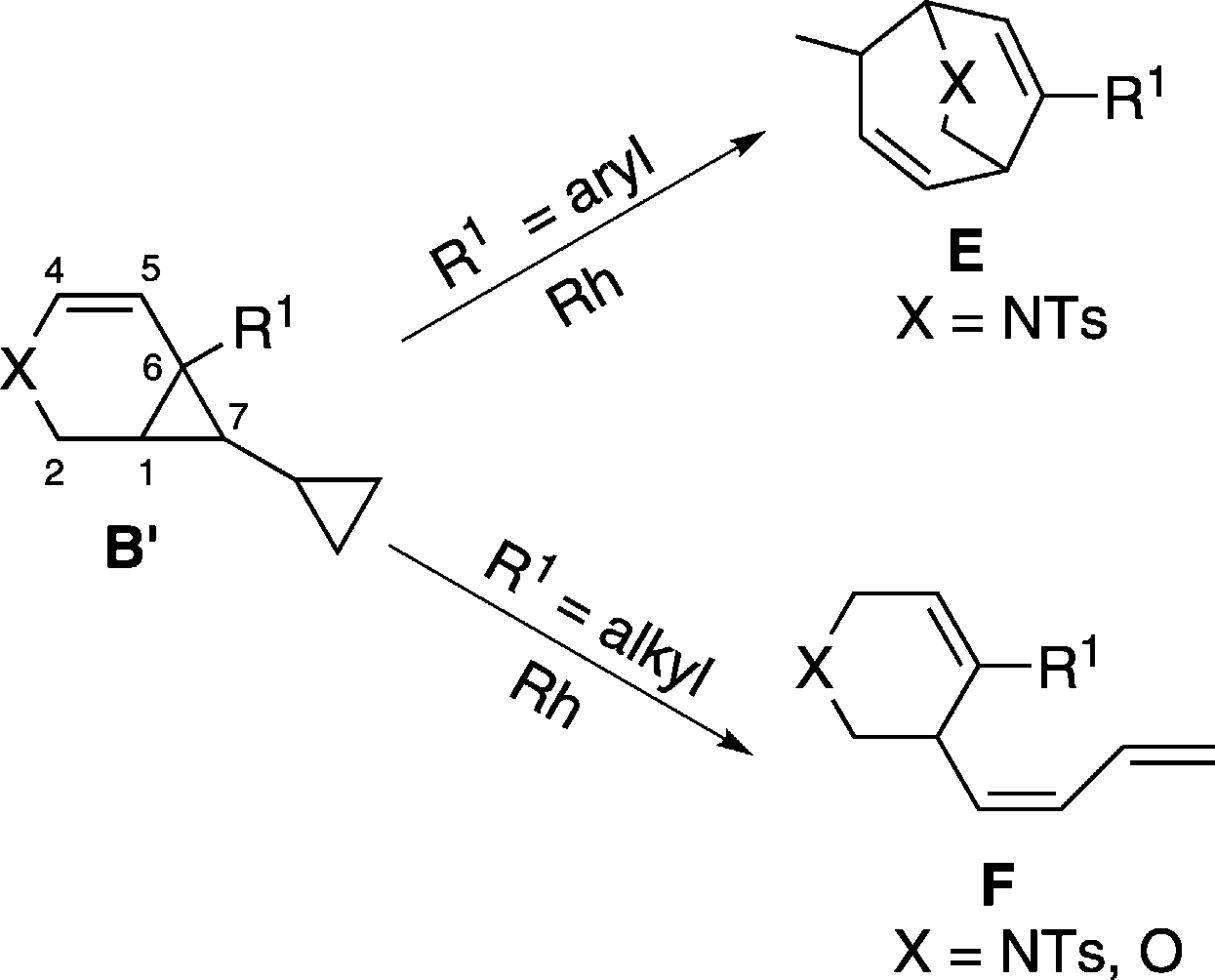
Rhodium-catalyzed isomerisation of bicyclo[4.1.0]heptenes.

Bicyclo[4.1.0]heptene derivatives were easily obtained from cyclopropylenynes by transition metal-catalyzed cycloisomerization reactions.4g, 5 6-Cyclopropylbicyclo[4.1.0]hept-2-ene derivatives **1 b**–**16 b** used in this study were easily obtained by PtCl_2_-catalyzed cycloisomerization of the corresponding enynes (**1 a**–**16 a**; Scheme 3 and Table S1 in the Supporting Information).^6^

**Scheme 3:**
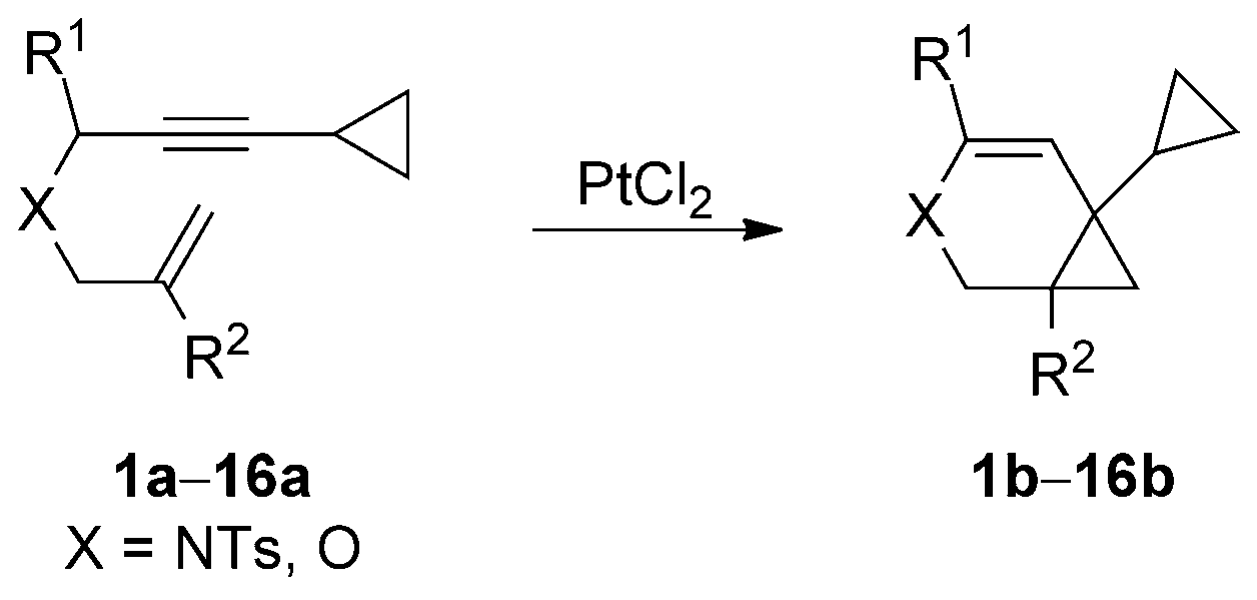
Synthesis of 6-cyclopropylbicyclo[4.1.0]hept-2-ene derivatives **1 b**–**16 b**.

In the hope of finding a new transformation of bis-cyclopropane compounds into other compounds, we treated **1 a** (X=*N*-tosyl [NTs], R^1^=H, R^2^=Me) with a cationic rhodium catalyst Rh(PPh_3_)_2_(CO)Cl/AgSbF_6_. However, intractable compounds were obtained. When **2 a** (X=NTs, R^1^=H, R^2^=Ph) was reacted with Rh(PPh_3_)_2_(CO)Cl/AgSbF_6_ in 1,4-dioxane at 100 °C for 24 h, we were able to confirm the formation of new cyclic compound **2 c** in 20 % yield (Scheme 4).

**Scheme 4:**
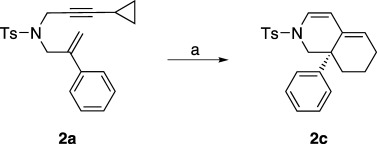
*Reagents and conditions:* a) Rh(PPh_3_)_2_(CO)Cl/AgSbF_6_, 1,4-dioxane, 100 °C, 24 h, 20 %.

Encouraged by this observation, we screened the conditions, including the rhodium species, counteranion, reaction solvent, reaction time, and reaction temperature (Table 1). Yields of the reaction were highly sensitive to the reaction solvent. When 1,2-dichloroethane was used instead of 1,4-dioxane, the yield dramatically increased to 68 % (Entry 2). As the amount of the catalyst used was increased, the yield also increased (Entries 3 and 4). Reaction conditions with a carbon monoxide atmosphere were found to be detrimental to the yield (Entry 5). The yield of the reaction was highly dependent upon the counteranion (Entries 2 and 6–10). When the counteranion was NO_3_^−^ or PF_6_^−^ (Entries 6 and 7), no reaction was observed. Moreover, in cases of ClO_4_^−^ and TfO^−^, very poor yields were observed (14 and 10 % yield, respectively; Entries 8 and 9). The BF_4_^−^ anion was found to give the best yield in 1,2-dichloroethane (74 %; Entry 10). We also screened other neutral and cationic rhodium compounds, such as [Rh(CO)Cl(dppe)]_2_/AgSbF_6_, [RhCl(COD)]_2_, [RhCl(COD)]_2_/AgSbF_6_, [RhCl(COD)]_2_/P(4-FC_6_H_4_)_3_/AgSbF_6_, [PPN][Rh(CO)_2_Cl_2_]/AgSbF_6_, and [Rh(CO)_2_Cl]_2_ and [RhCl(CO)(dppp)]_2_ under a carbon monoxide atmosphere [Entries 12–17; dppe=1,2-bis(diphenylphosphino)ethane, dppp=1,3-bis(diphenylphosphino)propane, PPN=bis(triphenylphosphoranylidene)ammonium]. Unfortunately, they were found to be ineffective. Thus, we established that Rh(PPh_3_)_2_(CO)Cl/AgBF_4_ in 1,2-dichloroethane was the best catalyst system in respect to the yield. The formation of **2 c** was confirmed using ^1^H NMR and ^13^C NMR spectroscopy, high-resolution mass spectrometry (HRMS), and X-ray crystallography (Figure 1).^6^

**Table 1:** Variety of rhodium-catalyzed rearrangement reactions of 2 B

Entry	Catalyst (mol %)	Solvent	Time [h]	Temp [°C]	Yield[a] [%]
1	Rh(CO)(PPh_3_)_2_Cl (10)/AgSbF_6_ (12)	1,4-dioxane	4	100	36
2	Rh(CO)(PPh_3_)_2_Cl (10)/AgSbF_6_ (12)	1,2-dichloroethane	4	80	68
3	Rh(CO)(PPh_3_)_2_Cl (5)/AgSbF_6_ (9)	1,4-dioxane	24	100	trace
4	Rh(CO)(PPh_3_)_2_Cl (20)/AgSbF_6_ (24)	1,4-dioxane	1	100	54
5[b]	Rh(CO)(PPh_3_)_2_Cl (10)/AgSbF_6_ (12)	1,2-dichloroethane	4	80	24
6	Rh(CO)(PPh_3_)_2_Cl (10)/AgNO_3_ (12)	1,2-dichloroethane	24	80	NR
7	Rh(CO)(PPh_3_)_2_Cl (10)/AgPF_6_ (12)	1,2-dichloroethane	24	80	NR
8[c]	Rh(CO)(PPh_3_)_2_Cl (10)/AgClO_4_ (12)	1,2-dichloroethane	1	80	14
9[d]	Rh(CO)(PPh_3_)_2_Cl (10)/AgOTf (12)	1,2-dichloroethane	1	80	10
10	Rh(CO)(PPh_3_)_2_Cl (10)/AgBF_4_ (12)	1,2-dichloroethane	4	80	74
11	RhCl(CO)(dppe) (10)/AgSbF_6_ (12)	1,4-dioxane	24	100	NR
12	[RhCl(COD)]_2_ (10)	toluene	17	100	trace
13	[RhCl(COD)]_2_ (10)/AgSbF_6_	toluene	24	100	NR
14	[Rh(CO)_2_Cl]_2_ (5)/P(4-FC_6_H_4_)_3_ (20)/AgSbF_6_ (12)	1,2-dichloroethane	24	80	NR
15	[PPN][Rh(CO)_2_Cl_2_] (4)/AgSbF_6_ (8)	dichloromethane	24	30	NR
16[e]	[Rh(CO)_2_Cl]_2_ (5)	tetrahydrofuran	24	60	NR
17[e]	[RhCl(CO)(dppp)]_2_ (10)	1,2-dichloroethane	24	80	NR

[a] Isolated yield.

[b] Under CO (1 atm).

[c] 16 % of reactant remained.

[d] 14 % of reactant remained.

[e] Under CO (13 atm). NR=no reaction.

**Figure 1:**
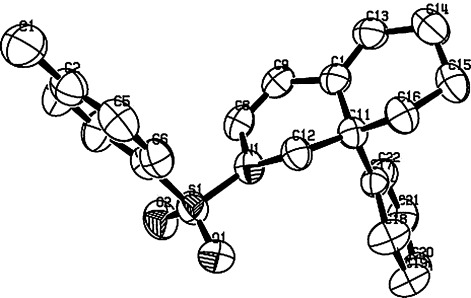
X-ray crystal structure of **2 c**.

Using Rh(PPh_3_)_2_(CO)Cl/AgBF_4_ as a catalyst, we investigated the rhodium(I)-catalyzed transformation of various NTs-tethered enynes to arylhexahydroisoquinolines (Table 2). Almost all the reactions went to completion within 4 h. Moderate to high yields were observed for all enynes bearing an aryl with an electron-withdrawing (Entries 4–6 and 10) or donating group (Entries 2, 3, and 7–9). It seems that the electronic nature of a substituent on the aryl ring does not exert any noticeable influence on the yield of the reaction. However, the position of the substituent (3- versus 4-position; cf. Entry 2 and 8, 3 and 9, and 4 and 10) has a slight influence on the yield of the reaction. Substrates with a substituent at the 4-position produce 10 % higher yields than those with a substituent at the 3-position. Interestingly, in the case of the *tert*-butyl group, a rather low yield is obtained when the substituent is located at the 4-position (60 %; Entry 7). As expected, a substituent at the 2-position exerts a dramatic effect on the yield of the reaction. Thus, enynes having an aryl with *ortho* substituents did not give any products (Entries 12 and 13) and the reactant was recovered intact. Introduction of a methyl group to the α-position of the NTs group did not have any effect on the yield of the reaction (Entry 15). Unfortunately, all attempts with *O*-tethered substrate **16 b** were unsuccessful. The substrate **16 b** readily decomposed in the presence of the catalytic system and no characterizable products were isolated. Thus, the transformation of the bis-cyclopropanyl derivatives to hexahydronaphthalenes is unique to the NTs-tethered substrates.

**Table 2:** Rhodium-catalyzed rearrangement reaction[a]

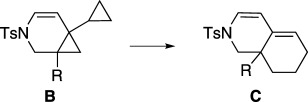							
Entry	Substrate		R	Time [h]	Product		Yield[b] [%]
1	**2 b**		C_6_H_5_	4	**2 c**		74
2	**3 b**		4-CH_3_C_6_H_4_	5	**3 c**		72
3	**4 b**		4-CH_3_OC_6_H_4_	4	**4 c**		70
4	**5 b**		4-ClC_6_H_4_	4	**5 c**		73
5	**6 b**		4-FC_6_H_4_	4	**6 c**		72
6	**7 b**		4-F_3_CC_6_H_4_	4	**7 c**		79
7	**8 b**		4-*t*-BuC_6_H_4_	4	**8 c**		60
8	**9 b**		3-CH_3_C_6_H_4_	3	**9 c**		67
9	**10 b**		3-CH_3_OC_6_H_4_	4	**10 c**		62
10	**11 b**		3-ClC_6_H_4_	4	**11 c**		58
11	**12 b**		*m*-xylene	4	**12 c**		50
12	**13 b**		naphthyl	24	no reaction		
13	**14 b**		mesitylene	24	no reaction		
14	**15 b**	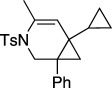	–	4	**15 c**	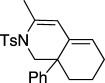	70
15	**1 b**	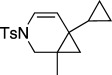	–	24	no reaction		
16	**16 b**	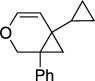	–	–	decomposed		

[a] *Reagents and conditions:* substrate, [Rh(CO)(PPh_3_)_2_Cl] (10 mol %), AgBF_4_ (12 mol %), 1,2-dichloroethane, 80 °C.

[b] Isolated yield.

Next, we investigated the possibility of integrating the platinum- and rhodium-catalyzed reactions into a “one-pot” transformation. However, none of the attempts were promising, presumably due to the different reaction conditions of each catalyzed reaction. The first step was usually carried out in toluene and the second step in 1,2-dichloroethane. When the second step was carried out in toluene, the yield of the second step was 36 %. Thus, we had to find a solvent that could be used in both steps. After much experimentation, 1,2-dichloroethane was chosen as the solvent. However, the integration of the two reactions in 1,2-dichloroethane afforded the expected product in up to 43 % yield (Scheme 5).

**Scheme 5:**
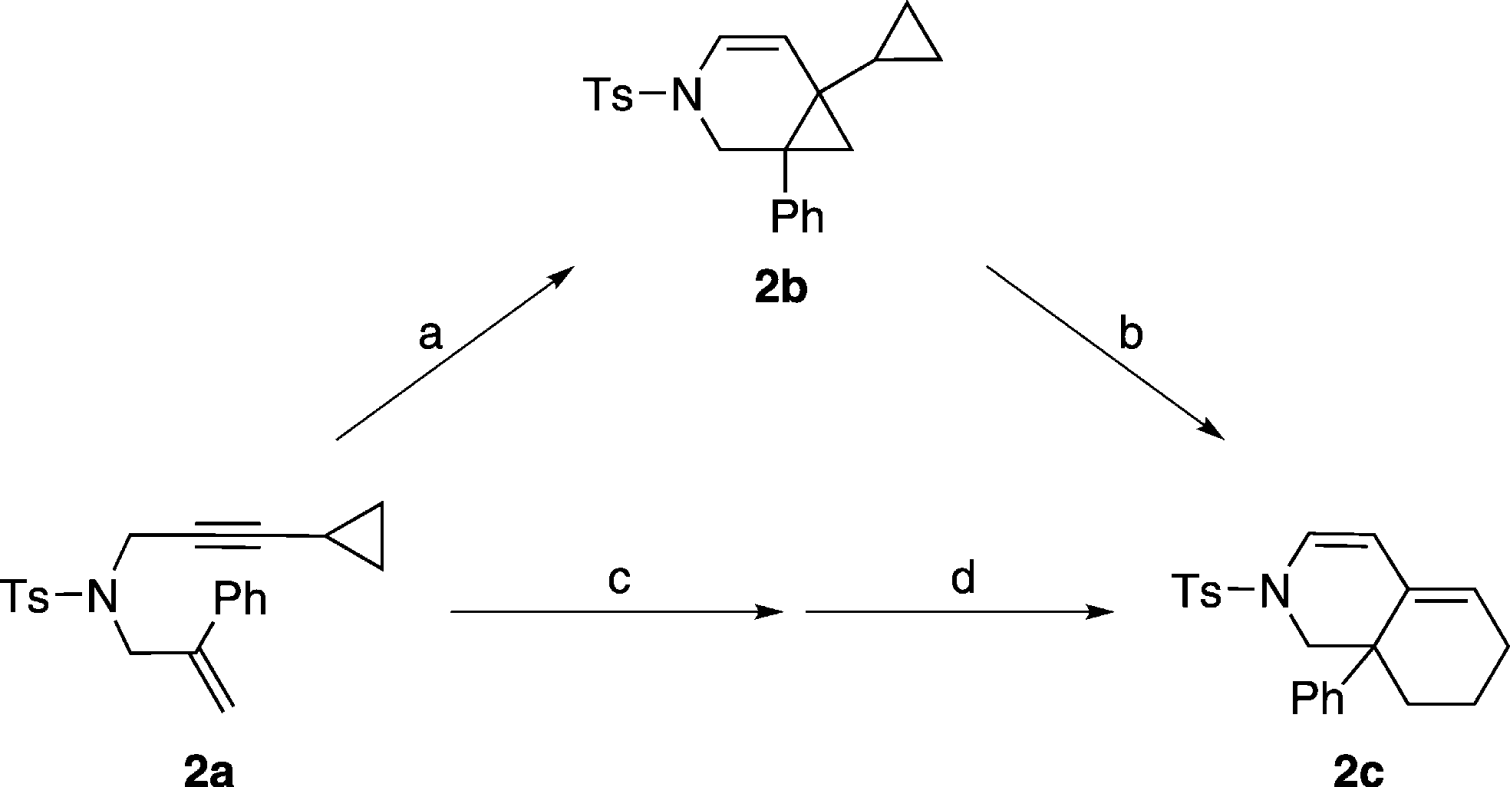
*Reagents and conditions:* a) PtCl_2_ (5 mol %), toluene, 80 °C, 7 h, 83%; b) RhCl(CO)(PPh_3_)_2_ (10 mol %), AgBF_4_ (12 mol %), 1,2-dichloroethane, 80 °C, 4 h, 74 %; c) PtCl_2_ (5 mol %), 1,2-dichloroethane, 80 °C, 15 h; d) RhCl(CO)(PPh_3_)_2_ (10 mol %), AgBF_4_ (12 mol %), 1,2-dichloroethane, 80 °C, 4 h, 43 % over c) and d).

Although little mechanistic information has been obtained, a plausible mechanism is proposed on the basis of the above results and previous studies (Scheme 6). The precoordination of the rhodium(I) center to the double bond (**I**) leads to the formation of a (π-allyl)(σ-alkyl)rhodium(III) intermediate (**II**). Intermediate **III** may be stabilized by the coordination of an aryl group.^7^ Successive insertions leading to the metallacycle (**IV**) followed by a reductive elimination allows the formation of the product (**C**).

**Scheme 6:**
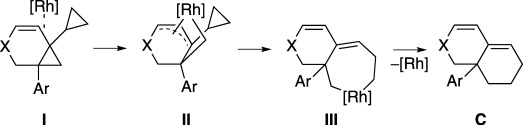
Plausible reaction mechanism.

In conclusion, we have developed a rhodium-catalyzed rearrangement reaction of azabicyclo[4.1.0]heptenes bearing an aryl and a cyclopropyl group in the 1- and 6-position, respectively, to arylhexahydroisoquinolines, which are difficult to prepare by conventional synthetic pathways. The reaction pathway is highly sensitive to the identity and position of substituents at the cyclopropyl moiety. The reaction was shown to be an efficient and simple method for the synthesis of arylhexahydroisoquinolines.

## Experimental Section

**General Procedure**: [RhCl(CO)(PPh_3_)_2_] (0.03 mmol), AgBF_4_ (0.036 mmol) and 1,2-dichloroethane (2 mL) were added to a tube-type Schenk flask equipped with a stirring bar and capped with a rubber septum. The reaction mixture was stirred at RT for 5 min. The substrate (0.3 mmol) and 1,2-dichloroethane (2 mL) were added to the flask. The resulting mixture was stirred at 80 °C until the substrate completely disappeared (as analyzed by thin-layer chromatography). The reaction products were purified by flash chromatography on a silica-gel column eluting with *n*-hexane/ethyl acetate (20:1, *v*/*v*).

## Supplementary material

Detailed facts of importance to specialist readers are published as ”Supporting Information”. Such documents are peer-reviewed, but not copy-edited or typeset. They are made available as submitted by the authors.
