# Supplementary material for: Rhodium-Catalyzed Rearrangement Reaction of Azabicyclo[4.1.0]heptenes bearing Cyclopropyl and Aryl Groups to Arylhexahydroisoquinolines
Source: ChemistryOpen. 2012 Jul 16;1(4):169–72. doi: 10.1002/open.201200022 (PMC3922445; doi:10.1002/open.201200022)
Supplement: Supplementary file 1 [file open0001-0169-SD1.pdf]

ChemistryOPEN

Supporting Information

© Copyright Wiley-VCH Verlag GmbH & Co. KGaA, 69451 Weinheim, 2012

Rhodium-Catalyzed Rearrangement Reaction of Azabicyclo[4.1.0]heptenes bearing Cyclopropyl and Aryl Groups to Arylhexahydroisoquinolines

Sori Son, Sun Young Kim, and Young Keun Chung*[a]

open_201200022_sm_miscellaneous_information.pdf

General Experimental

All solvents were dried and distilled according to standard methods before use. [RhCl(CO)(PPh_3)$_2$] was prepared according to the literature procedures.[1] Silver tetrafluoroborate ($AgBF_4$) was purchased from Acros Organics. Reactions were carried out in a flame-dried glassware equipped with a stirring bar and sealed with a rubber septum under N_2, unless otherwise indicated. Elevated temperatures were maintained in thermostat-controlled oil baths. Reactions were monitored by thin-layer chromatography carried out on 0.25 mm E. Merck silica gel plates (60F-254) using UV light as a visualizing agent and acidic *p*-anisaldehyde, and heat as a developing agent. Flash chromatography was carried out on Merck 60 silica gel (230 – 400 mesh). ^{1}H and ^{13}C NMR spectra were recorded with Bruker (300 MHz) spectrometer. ^{1}H NMR spectra were referenced to residual TMS (0 ppm) and reported as follows: chemical shift, multiplicity (s = singlet, d = doublet, t = triplet, q = quartet, m = multiplet). Chemical shifts of the ^{13}C NMR spectra were measured relative to $CDCl_3$ (75.00 ppm). Mass spectral data were obtained from the Korea Basic Science Institute (Daegu) on a Jeol JMS 700 high resolution mass spectrometer. Single crystal data for **1a** were collected on an Enraf-Nonius CCD single crystal X-ray diffractometer at room temperature using graphite-monochromated Mo K α radiation (λ = 0.71073 Å). The structure was solved by direct methods using SHELXS-97 and refined by full-matrix least-squares with SHELXL-97.

Preparation of 2-arylprop-2-en-1-ol

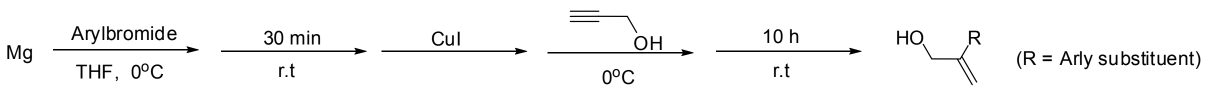

Magnesium turnings (1.24 g, 51 mmol) were dissolved in THF (20 mL) in a round-bottomed flask equipped with a stirrer. After aryl bromide (51 mmol) was added to the solution at 0℃, the resulting solution was stirred for 30 min. Then, copper iodide (0.16 g, 0.8 mmol) was also added. After propargyl alcohol (1mL, 17 mmol) was dropwisely added to the solution at 0℃, then resulting solution was stirred at room temperature for 10 h. The reaction mixture was quenched with NH_4Cl solution. The reaction product was extracted with ethyl acetate. The extract was dried over anhydrous $MgSO_4$, and was evaporated to give a crude product. Flash chromatography on a silica gel eluting with hexane and ethyl acetate (v/v, 9:1) afforded 2-arylprop-2-en-1-ol.

Representative procedure for preparation of N-(3-cyclopropylprop-2-ynyl)-4-methyl-N-(2-phenylallyl)benzenesulfonamide (2A)

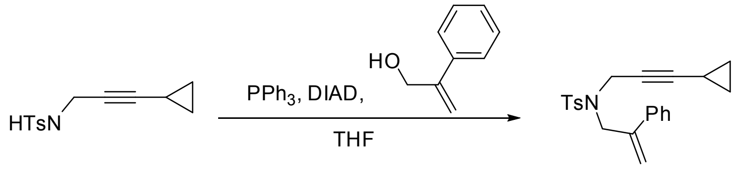

To a solution of N-(3-cyclopropylprop-2-ynyl)-4-methylbenzenesulfonamide(0.872 g, 3.5 mmol) and PPh_3 (0.92 g, 3.5 mmol) in THF (30 mL) was added DIAD (DiisopropylAzodicarboxylate) (0.68 mL, 3.5 mmol) at room temperature. To the solution, 2-phenylprop-2-en-1-ol was added. The reaction mixture was stirred for 20 h at room temperature. After the solvent was removed by a rotary evaporator, the residue was purified by flash column chromatography eluting with 10% EtOAc in hexane.

6-cyclopropyl-1-phenyl-3-tosyl-3-azabicyclo[4.1.0]hept-4-ene (2B)

N-(3-Cyclopropylprop-2-ynyl)-4-methyl-N-(2-phenylallyl)benzenesulfonamide (0.365 g, 1 mmol) was added to a solution of 5 mol% $PtCl_2$ (0.013 g, 0.05 mmol) in toluene (10 mL). The resulting mixture was stirred until the substrate was completely disappeared (as checked by TLC) at 80℃. The reaction mixture was purified by flash chromatography eluting with n-hexane/ethyl acetate (v/v = 9 : 1) to afford 6-cyclopropyl-1-phenyl-3-tosyl-3-azabicyclo[4.1.0]hept-4-ene (**2B**) as white solid (0.29 g, 83%).

**Table S1.** Platinum-Catalyzed Rearrangement Reaction[a]			
Entry	Substrate	Time (h)	Product[b]
	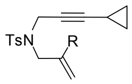<br>**A**		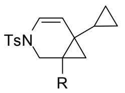<br>**B**
	R		
1	C_6H_5 (**2A**)	7	83 (**2B**)
2	4-$CH_3C_6H_4$ (**3A**)	4	85 (**3B**)
3	4-$CH_3OC_6H_4$ (**4A**)	4	84 (**4B**)
4	4-ClC_6H_4 (**5A**)	4	79 (**5B**)
5	4-FC_6H_4 (**6A**)	4	80 (**6B**)
6	4-$F_3CC_6H_4$ (**7A**)	4	80 (**7B**)
7	4-tert-butylbenzene (**8A**)	5	59 (**8B**)
8	3-$CH_3C_6H_4$ (**9A**)	4	77 (**9B**)
9	3-$CH_3OC_6H_4$ (**10A**)	3	80 (**10B**)
10	3-ClC_6H_4 (**11A)**	4	71 (**11B**)
11	m-xylene (**12A**)	4	60 (**12B**)
12	naphthyl (**13A**)	4	79 (**13B**)
13	Mesitylene (**14A**)	5	62 (**14B**)
14	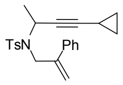<br>**15 A**	19	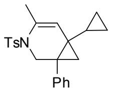<br>**15 B**<br>74
15	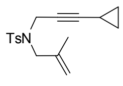<br>**1 A**	4	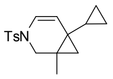<br>**1 B**<br>65
16	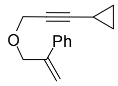<br>**16 A**	4	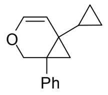<br>**16 B**<br>75

[a] Reaction conditions: substrate and 5 mol% $PtCl_2$ were reacted in toluene at 80℃. [b] Isolated yield.

Characterization of Compounds

N-(3-cyclopropylprop-2-ynyl)-4-methyl-N-(2-methylallyl)benzenesulfonamide (1A)
^{1}H NMR (300 MHz, $CDCl_3$) δ 7.73 (d, *J* = 8.2 Hz, 2 H), 7.30 (d, *J* = 8.4 Hz, 2 H), 4.95 (s, 2 H), 3.982 (s, 1 H), 3.976 (s, 1 H), 3.70 (s, 2 H), 2.44 (s, 3 H), 1.76 (s, 3 H), 0.85 – 0.96 (m, 1 H), 0.56 – 0.63 (m, 2 H), 0.26 – 0.31 (m, 2 H) ppm. **^{13}C NMR (75 MHz, $CDCl_3$)** δ 143.22, 139.40, 136.28, 129.30, 127.81, 115.20, 89.26, 67.52, 52.31, 36.02, 21.52, 19.74, 7.82, -0.94 ppm. **HRMS (EI)** calc. for [$C_{17}H_{21}O_2SN$]: 303.1293, found 303.1293.

N-(3-cyclopropylprop-2-ynyl)-4-methyl-N-(2-phenylallyl)benzenesulfonamide (2A)
^{1}H NMR (300 MHz, $CDCl_3$) δ 7.74 (d, *J* = 8.1 Hz, 2 H), 7.53 (d, *J* = 6.8 Hz, 2 H), 7.25 – 7.37 (m, 5 H), 5.56 (s, 1 H), 5.32 (s, 1 H), 4.22 (s, 2 H), 3.93 (s, 2 H), 2.45 (s, 3 H), 0.89 – 0.95 (m, 1 H), 0.57 – 0.63 (m, 2 H), 0.25 – 0.30 (m, 2 H) ppm. **^{13}C NMR (75 MHz, $CDCl_3$)** δ 143.39, 141.45, 137.84, 135.82, 129.36, 128.44, 128.12, 127.99, 126.43, 116.99, 89.73, 67.36, 49.93, 36.10, 21.55, 7.86, -0.82 ppm. **HRMS (EI)** calc. for [$C_{22}H_{23}NO_2S$]: 365.1450, found: 365.1447.

N-(3-cyclopropylprop-2-ynyl)-4-methyl-N-(2-p-tolylallyl)benzenesulfonamide (3A)
^{1}H NMR (300 MHz, $CDCl_3$) δ 7.74 (d, *J* = 7.9 Hz, 2 H), 7.43 (d, *J* = 7.8 Hz, 2 H), 7.31 (d, *J* = 7.8 Hz, 2 H), 7.15 (d, *J* = 7.6 Hz, 2 H), 5.52 (s, 1 H), 5.26 (s, 1 H), 4.20 (s, 2 H), 3.92 (s, 2 H), 2.45 (s, 3 H), 2.35 (s, 3 H), 0.93 (br s, 1 H), 0.60 (d, *J* = 6.1 Hz, 2 H), 0.27 (d, *J* = 2.3 Hz, 2 H) ppm. **^{13}C NMR (75 MHz, $CDCl_3$)** δ 143.30, 141.22, 137.95, 135.85, 134.87, 129.31, 129.11, 128.00, 126.29, 116.14, 89.62, 67.39, 49.95, 36.03, 21.53, 21.15, 7.83, -0.95 ppm. **HRMS (EI)** calc. for [$C_{23}H_{25}NO_2S$]: 379.1606, found: 379.1610.

N-(3-cyclopropylprop-2-ynyl)-N-(2-(4-methoxyphenyl)allyl)-4-methylbenzenesulfonamide (4A)
^{1}H NMR (300 MHz, $CDCl_3$) δ 7.74 (d, *J* = 8.3 Hz, 2 H), 7.50 (d, *J* = 8.8 Hz, 2 H), 7.32 (d, *J* = 8.1 Hz, 2 H), 6.88 (d, *J* = 8.8 Hz, 2 H), 5.48 (s, 1 H), 5.21 (s, 1 H), 4.19 (s, 2 H), 3.91 (d, *J* = 1.8Hz, 2 H), 3.81 (s, 3 H), 2.45 (s, 3 H), 0.88 - 0.97 (m, 1 H), 0.56 - 0.63 (m, 2 H), 0.24 - 0.29 (m, 2 H) ppm. **^{13}C NMR (75 MHz, $CDCl_3$)** δ 159.53, 143.32, 140.58, 135.76, 130.07, 129.30, 128.00, 127.59, 115.35, 113.76, 89.62, 67.31, 55.26, 50.03, 35.95, 21.51, 7.81, -0.97 ppm. **HRMS (EI)** calc. for [$C_{23}H_{25}NO_2S$]: 395.1555, found: 395.1555.

N-(2-(4-chlorophenyl)allyl)-N-(3-cyclopropylprop-2-ynyl)-4-methylbenzenesulfonamide (5A)
^{1}H NMR (300 MHz, $CDCl_3$) δ 7.73 (d, *J* = 8.2 Hz, 2 H), 7.48 (d, *J* = 8.6 Hz, 2 H), 7.30 – 7.33 (m, 4 H), 5.55 (s, 1 H), 5.33 (s, 1 H), 4.19 (s, 2 H), 3.90 (d, *J* = 1.7Hz, 2 H), 2.46 (s, 3 H), 0.93 (dd, *J* = 4.0, 9.1 Hz, 1 H), 0.57 – 0.64 (m, 2 H), 0.24 – 0.29 (m, 2 H) ppm. **^{13}C NMR (75 MHz, $CDCl_3$)** δ 143.51, 140.42, 136.10, 135.64, 134.01, 129.39, 128.61, 127.97, 127.78, 117.61, 89.84, 67.13, 49.90, 36.05, 21.56, 7.87, -0.96 ppm. **HRMS (EI)** calc. for [$C_{22}H_{22}NO_2ClS$]: 399.1060, found: 399.1060.

N-(3-cyclopropylprop-2-ynyl)-N-(2-(4-fluorophenyl)allyl)-4-methylbenzenesulfonamide (6A)
^{1}H NMR (300 MHz, $CDCl_3$) δ 7.75 (d, *J* = 8.2 Hz, 2 H), 7.54 (m, 2 H), 7.33 (d, *J* = 8.1 Hz, 2 H), 7.04 (t, *J* = 8.7 Hz, 2 H), 5.53 (s, 1 H), 5.31 (s, 1 H), 4.21 (s, 2 H), 3.92 (d, *J* = 1.5 Hz, 2 H), 2.46 (s, 3 H), 0.90 – 0.96 (m, 1 H), 0.58 - 0.65 (m, 2 H), 0.25 – 0.31 (m, 1H) ppm. **^{13}C NMR (75 MHz, $CDCl_3$)** δ 143.50, 140.37, 135.63, 129.38, 128.22, 128.11, 127.95, 117.04, 115.43, 115.14, 89.84, 67.11, 50.04, 36.03, 21.54, 7.86, -0.96 ppm. **HRMS (EI)** calc. for [$C_{22}H_{22}FNO_2S$]: 383.1355, found: 383.1359.

N-(3-cyclopropylprop-2-ynyl)-4-methyl-N-(2-(4-trifluoromethyl)phenyl)allyl)benzenesulfonamide (7A)
^{1}H NMR (300 MHz, $CDCl_3$) δ 7.73 (d, *J* = 8.2 Hz, 2 H), 7.67 (d, *J* = 8.3 Hz, 2 H), 7.59 (d, *J* = 8.4 Hz, 2 H), 7.32 (d, *J* = 8.1 Hz, 2 H), 5.65 (s, 1 H), 5.44 (s, 1 H), 4.25 (s, 2 H), 3.91 (d, *J* = 1.3 Hz, 2 H), 2.44 (s, 3 H), 0.91 – 0.95 (m, 1 H), 0.68 – 0.64 (m, 2 H), 0.25 – 0.29 (m, 1 H) ppm. **^{13}C NMR (75 MHz, $CDCl_3$)** δ 143.66, 140.45, 135.53, 129.43, 127.92, 126.81, 125.42, 125.36, 125.31, 119.26, 90.07, 66.91, 49.86, 36.10, 21.46, 7.85, -1.00 ppm. **HRMS (EI)** calc. for [$C_{23}H_{22}F_3NO_2S$]: 433.1323, found: 433.1325.

N-(2-(4-tert-butylphenyl)allyl)-N-(3-cyclopropylprop-2-ynyl)-4-methylbenzenesulfonamide (8A)
^{1}H NMR (300 MHz, $CDCl_3$) δ 7.67 (d, *J* = 8.2 Hz, 2 H), 7.42 (d, *J* = 8.4 Hz, 2 H), 7.23 – 7.31 (m, 4 H), 5.48 (s, 1 H), 5.20 (s, 1 H), 4.14 (s, 2 H), 3.86 (d, *J* = 1.5Hz, 2 H), 2.38 (s, 3 H), 1.25 (s, 9 H), 0.82 – 0.86 (m, 1 H), 0.51 – 0.54 (m, 2 H), 0.19 – 0.22 (m, 2 H) ppm. **^{13}C NMR (75 MHz, $CDCl_3$)** δ 151.12,

143.34, 141.02, 135.84, 134.82, 129.33, 128.02, 126.06, 125.37, 116.30, 89.67, 67.42, 49.94, 36.08, 34.56, 31.29 , 21.55, 7.85, -0.93 ppm. **HRMS (EI)** calc. for [$C_{26}H_{31}NO_2S$]: 421.2076, found: 421.2074.

N-(3-cyclopropylprop-2-ynyl)-4-methyl-N-(2-m-tolylallyl)benzenesulfonamide (9A)
^{1}H NMR (300 MHz, $CDCl_3$) δ 7.74 (d, *J* = 8.2 Hz, 2 H), 7.32 (br s, 2 H), 7.10 – 7.32 (m, 4 H), 5.53 (s, 1 H), 5.30 (s, 1 H), 4.20 (s, 2 H), 3.94 (d, *J* = 1.6Hz, 2 H), 2.45 (s, 3 H), 2.36 (s, 3 H), 0.90 – 1.01 (m, 1 H), 0.57 – 0.64 (m, 2 H), 0.26 – 0.31 (m, 2 H) ppm. **^{13}C NMR (75 MHz, $CDCl_3$)** δ 143.29, 141.58, 137.91, 135.91, 129.49, 129.31, 128.84, 128.29, 127.95, 127.16, 123.52, 116.66, 89.63, 67.45, 49.90, 36.10, 21.51, 21.47, 7.82, -0.95 ppm. **HRMS (EI)** calc. for [$C_{23}H_{25}NO_2S$]: 379.1606, found: 379.1606.

N-(3-cyclopropylprop-2-ynyl)-N-(2-(3-methoxyphenyl)allyl)-4-methylbenzenesulfonamide (10A)
^{1}H NMR (300 MHz, $CDCl_3$) δ 7.74 (d, *J* = 8.1 Hz, 2 H), 7.23 – 7.33 (m, 4 H), 7.12 (d, *J* = 6.4Hz, 1 H), 6.86 (d, *J* = 7.1 Hz, 1 H), 5.58 (s, 1 H), 5.32 (s, 1 H), 4.21 (s, 2 H), 3.94 (s, 2 H), 3.84 (s, 3 H), 2.45 (s, 3 H), 0.91 – 0.94 (m, 1 H), 0.57 – 0.63 (m, 2 H), 0.25 – 0.30 (m, 2 H) ppm. **^{13}C NMR (75 MHz, $CDCl_3$)** δ 159.63, 143.37, 141.28, 139.25, 135.85, 129.35(Ts carbon + 3-MeO carbon), 127.96, 118.78, 117.15, 114.08, 111.86, 89.74, 67.34, 55.36, 49.97, 36.11, 21.54, 7.86, -0.93 ppm. **HRMS (EI)** calc. for [$C_{23}H_{25}NO_3S$]: 395.1555, found: 395.1552.

N-(2-(3-chlorophenyl)allyl)-N-(3-cyclopropylprop-2-ynyl)-4-methylbenzenesulfonamide (11A)
^{1}H NMR (300 MHz, $CDCl_3$) δ 7.73 (d, *J* = 8.2Hz, 2 H), 7.42 – 7.45 (m, 1 H), 7.27 – 7.33 (m, 5 H), 5.56 (s, 1 H), 5.37 (s, 1 H), 4.19 (s, 2 H), 3.93 (d, *J* = 1.6 Hz, 2 H), 2.45 (s, 3 H), 0.87 – 0.99 (m, 1 H), 0.61 (dt, *J* = 4.1, 6.7 Hz, 2 H), 0.26 – 0.32 (m, 2 H) ppm. **^{13}C NMR (75 MHz, $CDCl_3$)** δ 143.50, 140.51, 139.80, 135.72, 134.32, 129.74, 129.40, 128.13, 127.96, 126.57, 124.781, 118.09, 89.90, 67.24, 49.79, 36.19. 21.56, 7.89, -0.93 ppm. **HRMS (EI)** calc. for [$C_{22}H_{22}NO_2SCl$]: 399.1060, found: 399.1060.

N-(3-cyclopropylprop-2-ynyl)-N-(2-(3,5-dimethylphenyl)allyl)-4-methylbenzenesulfonamide (12A)
^{1}H NMR (300 MHz, $CDCl_3$) δ 7.74 (d, *J* = 8.2 Hz, 2 H), 7.30 (d, *J* = 8.0 Hz, 2 H), 7.12 (s, 2 H), 6.94 (s, 1 H), 5.51 (s, 1 H), 5.28 (s, 1 H), 4.19 (s, 2 H), 3.94 (s, 2 H), 2.44 (s, 3 H), 2.32 (s, 6 H), 0.91 – 0.99 (m, 1 H), 0.57 – 0.64 (m, 2 H), 0.27 – 0.32 (m, 2 H) ppm. **^{13}C NMR (75 MHz, $CDCl_3$)** δ 143.29, 141.74, 138.01, 137.83, 136.01, 129.76, 129.33, 127.97, 124.33, 116.43, 89.63, 67.58, 49.94, 36.16, 21.55, 21.37, 7.87, -0.90 ppm. **HRMS (EI)** calc. for [$C_{24}H_{27}NO_2S$]: 393.1763, found: 393.1759.

N-(3-cyclopropylprop-2-ynyl)-4-methyl-N-(2-(naphthalen-1-yl)allyl)benzenesulfonamide (13A)
^{1}H NMR (300 MHz, $CDCl_3$) δ 8.00 – 8.03 (m, 1 H), 7.83 – 7.86 (m, 1 H), 7.78 (d, *J* = 8.1 Hz, 1 H), 7.63 (d, *J* = 8.2Hz, 2 H), 7.45 – 7.48 (m, 2 H), 7.41 (d, *J* = 8.0Hz, 1 H), 7.31 (d, *J* = 6.9Hz, 1 H), 7.18 (d, *J* = 8.1Hz, 2 H), 5.74 (s, 1 H), 5.35 (s, 1 H), 4.18 – 4.20 (m, 4 H), 2.37 (s, 3 H), 0.92 – 1.00 (m, 1 H), 0.59 (dt, *J* = 4.1, 6.6 Hz, 2 H), 0.27 – 0.32 (m, 2 H) ppm. **^{13}C NMR (75 MHz, $CDCl_3$)** δ 143.22, 142.13, 137.95, 136.23, 133.71, 131.28, 129.29, 128.40, 127.93, 127.66, 126.18, 125.81, 125.24, 117.89, 89.75, 67.83, 51.63, 36.92, 21.49, 7.88, -0.88 ppm. **HRMS (EI)** calc. for [$C_{26}H_{25}NO_2S$]: 415.1606, found: 415.1608.

N-(3-cyclopropylprop-2-ynyl)-N-(2-mesitylallyl)-4-methylbenzenesulfonamide (14A)
^{1}H NMR (300 MHz, $CDCl_3$) δ 7.70 (d, *J* = 8.2 Hz, 2 H), 7.25 (d, *J* = 8.0 Hz, 2 H), 6.87 (s, 2 H), 5.58 (s, 1 H), 5.02 (s, 1 H), 4.21 (s, 2 H), 3.84 (s, 2 H), 2.40 (s, 3 H), 2.27 (s, 3 H), 2.23 (s, 6 H), 0.87 (dd, *J* = 4.7, 11.6 Hz, 1 H), 0.54 – 0.60 (m, 2 H), 0.23 – 0.28 (m, 2 H) ppm. **^{13}C NMR (75 MHz, $CDCl_3$)** δ 143.26, 142.24, 136.89, 136.42, 135.80, 129.32, 128.23, 127.69, 114.14, 89.63, 67.99, 49.96, 37.23, 31.61, 22.67, 21.49, 20.97, 19.53, 14.14, 7.81, -0.96 ppm. **HRMS (EI)** calc. for [$C_{25}H_{29}NO_2S$]: 407.1919, found: 407.1915.

N-(4-cyclopropylbut-3-yn-2-yl)-4-methyl-N-(2-phenylallyl)benzenesulfonamide (15A)
^{1}H NMR (300 MHz, $CDCl_3$) δ 7.72 (d, *J* = 8.2 Hz, 2 H), 7.47 (m, 2 H), 7.25 – 7.37 (m, 5 H), 5.49 (d, *J* = 5.0 Hz, 2 H), 4.81 (q, *J* = 6.9 Hz, 1 H), 4.39 (d, *J* = 16.8Hz, 1 H), 3.98 (d, *J* = 16.8 Hz, 1 H), 2.43 (s, 3 H), 1.28 (d, *J* = 7.1Hz, 3 H), 0.93 – 1.02 (m, 1 H), 0.61 (dd, *J* = 2.3, 8.3 Hz, 2 H), 0.25 – 0.30 (m, 2 H) ppm. **^{13}C NMR (75 MHz, $CDCl_3$)** δ 144.78, 143.28, 139.26, 135.97, 129.35, 128.36, 127.85, 126.46, 115.28, 89.03, 72.17, 48.40, 47.15, 22.83, 21.52, 7.97, 7.89, -0.94 ppm. **HRMS (EI)** calc. for [$C_{23}H_{25}NO_2S$]: 379.1606, found: 397.1609.

(3-(3-cyclopropylprop-2-ynyloxy)prop-1-en-2-yl)benzene (16A)
^{1}H NMR (300 MHz, CDCl$_3$) δ 7.49 (d, *J* = 7.6 Hz, 2 H), 7.28 – 7.36 (m, 3 H), 5.53 (s, 1 H), 5.36 (s, 1 H), 4.44 (s, 2 H), 4.14 (s, 2 H), 1.24 – 1.30 (m, 1 H), 0.75 – 0.83 (m, 2 H), 0.67 – 0.73 (m, 2 H) ppm. **^{13}C NMR (75 MHz, CDCl$_3$)** δ 143.61, 138.62, 128.35, 127.78, 126.06, 115.03, 90.35, 86.48, 71.15, 57.61, 8.22, -0.51 ppm. **HRMS (EI)** calc. for [$C_{15}H_{16}O$]: 212.1201, found 212.1200.

6-cyclopropyl-1-methyl-3-tosyl-3-azabicyclo[4.1.0]hept-4-ene (1B)
^{1}H NMR (300 MHz, CDCl$_3$) δ 7.50 (d, *J* = 8.2 Hz, 2 H), 7.16 (d, *J* = 8.1 Hz, 2 H), 6.12 (d, *J* = 8.0 Hz, 1 H), 5.15 (d, *J* = 8.0 Hz, 1 H), 3.64 (d, *J* = 11.3 Hz, 1 H), 2.55 (d, *J* = 11.3 Hz, 1 H), 2.27 (s, 3 H), 1.02 (s, 3 H), 0.65 – 0.74 (m, 1 H), 0.35 – 0.41 (m, 1 H), 0.31 (d, *J* = 4.6 Hz, 1 H), 0.22 – 0.28 (m, 1 H), 0.16 (d, *J* = 4.4 Hz, 1 H), 0.01 (dd, *J* = 1.4, 4.7 Hz, 2 H) ppm. **^{13}C NMR (75 MHz, CDCl$_3$)** δ 143.59, 134.98, 129.74, 127.03, 120.12, 117.06, 46.67, 30.30, 23.58, 22.06, 21.55, 17.31, 12.40, 5.00, 2.83 ppm. **HRMS (EI)** calc. for [$C_{17}H_{21}O_2SN$]: 303.1293, found 303.1293.

6-cyclopropyl-1-phenyl-3-tosyl-3-azabicyclo[4.1.0]hept-4-ene (2B)
^{1}H NMR (300 MHz, CDCl$_3$) δ 7.63 – 7.66 (m, 2 H), 7.31 (d, *J* = 8.0 Hz, 2 H), 7.25 – 7.29 (m, 5 H), 6.40 (dd, *J* = 1.0, 8.1 Hz, 1 H), 5.34 (d, *J* = 8.1 Hz, 1 H), 3.96 (dd, *J* = 1.0, 11.5 Hz, 1 H), 2.99 (d, *J* = 11.5 Hz, 1 H), 2.43 (s, 3 H), 1.00 (d, *J* = 4.9 Hz, 1 H), 0.94 (d, *J* = 4.9 Hz, 1 H), 0.50 (m, 1 H), 0.33 – 0.42 (m, 1 H), 0.09 – 0.18 (m, 1 H), -0.14 - -0.05 (m, 1 H), -0.20 (m, 1 H) ppm. **^{13}C NMR (75 MHz, CDCl$_3$)** δ 143.72, 139.21, 134.94, 129.91, 129.82, 128.34, 127.09, 127.02, 121.23, 115.68, 48.09, 24.13, 21.58, 20.48, 13.82, 2.84 ppm. **HRMS (EI)** calc. for [$C_{22}H_{23}NO_2S$]: 365.1450, found: 365.1448.

6-cyclopropyl-1-p-tolyl-3-tosyl-3-azabicyclo[4.1.0]hept-4-ene (3B)
^{1}H NMR (300 MHz, CDCl$_3$) δ 7.63 (d, *J* = 8.2 Hz, 2 H), 7.30 (d, *J* = 8.1 Hz, 2 H), 7.16 (d, *J* = 8.0 Hz, 2 H), 7.09 (d, *J* = 8.0 Hz, 2 H), 6.38 (d, *J* = 8.1 Hz, 1 H), 5.31 (d, *J* = 8.1 Hz, 1 H), 3.94 (d, *J* = 11.3 Hz, 1 H), 2.95 (d, *J* = 11.5 Hz, 1 H), 2.43 (s, 3 H), 2.32 (s, 3 H), 1.00 (d, *J* = 4.8 Hz, 1 H), 0.92 (d, *J* = 4.8 Hz, 1 H), 0.49 (qd, *J* = 5.2, 8.2 Hz, 1 H), 0.33 – 0.42 (m, 1 H), 0.13 (m, 1 H), -0.07 (m, 1 H), -0.17 (m, 1 H) ppm. **^{13}C NMR (75 MHz, CDCl$_3$)** δ 143.69, 136.66, 136.17, 134.92, 129.80, 129.76, 129.04, 127.09, 121.15, 115.60, 48.12, 39.71, 24.08, 21.08, 21.14, 20.60, 13.83, 2.88, 2.82 ppm. **HRMS (EI)** calc. for [$C_{23}H_{25}NO_2S$]: 379.1606, found: 379.1605.

6-cyclopropyl-1-(4-methoxyphenyl)-3-tosyl-3-azabicyclo[4.1.0]hept-4-ene (4B)
^{1}H NMR (300 MHz, CDCl$_3$) δ 7.59 – 7.62 (m, 2 H), 7.27 (d, *J* = 8.0 Hz, 2 H), 7.13 – 7.17 (m, 2 H), 6.76 – 6.81 (m, 2 H), 6.34 (dd, *J* = 1.0, 8.1 Hz, 1 H), 5.28 (d, *J* = 8.1 Hz, 1 H), 3.91 (dd, *J* = 1.0, 11.5 Hz, 1 H), 3.75 (s, 3 H), 2.90 (d, *J* = 11.3 Hz, 1 H), 2.40 (s, 3 H), 0.95 (d, *J* = 4.9 Hz, 1 H), 0.86 (d, *J* = 5.2 Hz, 1 H), 0.46 (tt, *J* = 5.2, 8.2 Hz, 1 H), 0.30 – 0.39 (m, 1H), 0.08 (m, 1 H), -0.12 - -0.06 (m, 1 H), -0.26 - -0.17 (m, 1 H) ppm. **^{13}C NMR (75 MHz, CDCl$_3$)** δ 158.51, 143.68, 134.93, 131.31, 130.93, 129.80, 127.08, 121.09, 115.64, 113.69, 55.22, 48.11, 39.35, 24.14, 20.68, 13.78, 2.83, 0.00 ppm. **HRMS (EI)** calc. for [$C_{23}H_{25}NO_3S$]: 395.1555, found: 395.1558.

1-(4-chlorophenyl)-6-cyclopropyl-3-tosyl-3-azabicyclo[4.1.0]hept-4-ene (5B)
^{1}H NMR (300 MHz, CDCl$_3$) δ 7.64 (d, *J* = 8.2 Hz, 2 H), 7.32 (d, *J* = 8.1 Hz, 2 H), 7.19 – 7.27 (m, 4 H), 6.39 (d, *J* = 8.1 Hz, 1 H), 5.33 (d, *J* = 8.1 Hz, 1 H), 3.93 (d, *J* = 11.5 Hz, 1 H), 2.93 (d, *J* = 11.5 Hz, 1 H) , 2.44 (s, 3 H), 1.01 (d, *J* = 5.0 Hz, 1 H), 0.90 (d, *J* = 4.9 Hz, 1 H), 0.44 – 0.53 (m, 1 H), 0.40 (m, 1 H), 0.13 (m, 1 H), -0.11 - -0.03 (m, 1H) -0.21 (m, 1 H) ppm. **^{13}C NMR (75 MHz, CDCl$_3$)** δ 143.84, 137.76, 134.79, 131.26, 129.87, 128.59, 127.07, 121.38, 115.47, 47.91, 39.29, 24.23, 21.60, 20.41, 13.74, 2.97, 2.91 ppm. **HRMS (EI)** calc. for [$C_{22}H_{22}NO_2SCl$]: 399.1060, found: 399.1060.

6-cyclopropyl-1-(4-fluorophenyl)-3-tosyl-3-azabicyclo[4.1.0]hept-4-ene (6B)
^{1}H NMR (300 MHz, CDCl$_3$) δ 7.64 (d, *J* = 8.2 Hz, 2 H), 7.32 (d, *J* = 8.1 Hz, 2 H), 7.23 (dd, *J* = 6.1, 9.3 Hz, 2 H), 6.97 (t, *J* = 8.7 Hz, 2 H), 6.39 (d, *J* = 8.2 Hz, 1 H), 5.33 (d, *J* = 8.1 Hz, 1 H), 3.94 (d, *J* = 11.4 Hz, 1 H), 2.94 (d, *J* = 11.5 Hz, 1 H), 2.43 (s, 3 H), 0.99 (d, *J* = 4.9 Hz, 1 H), 0.89 (d, *J* = 5.3 Hz, 1 H), 0.49 (td, *J* = 4.1, 3.4 Hz, 1 H), 0.35 – 0.44 (m, 1 H), 0.13 (m, 1 H), -0.07 (m, 1 H), -0.22 (m, 1 H) ppm. **^{13}C NMR (75 MHz, CDCl$_3$)** δ 143.78, 134.89, 131.50, 131.39, 129.85, 127.06, 121.30, 115.56, 115.40, 48.05, 39.24, 24.20, 21.58, 20.55, 13.74, 2.88 ppm. **HRMS (EI)** calc. for [$C_{22}H_{22}NO_2FS$]: 383.1355, found: 383.1358.

6-cyclopropyl-3-tosyl-1-(4-(trifluoromethyl)phenyl)-3-azabicyclo[4.1.0]hept-4-ene (7B)

^{1}**H NMR (300 MHz, $CDCl_3$)** δ 7.64 (d, *J* = 8.2 Hz, 2 H), 7.55 (d, *J* = 8.1 Hz, 2 H), 7.39 (d, *J* = 8.1 Hz, 2 H), 7.32 (d, *J* = 8.1 Hz, 2 H), 6.42 (d, *J* = 8.1 Hz, 1 H), 5.35 (d, *J* = 8.1 Hz, 1 H), 3.94 (d, *J* = 11.5 Hz, 1 H), 2.98 (d, *J* = 11.5 Hz, 1 H), 2.44 (s, 3 H), 1.07 (d, *J* = 5.1 Hz, 1 H), 0.96 (d, *J* = 5.0 Hz, 1 H), 0.36 – 0.51 (m, 2 H), 0.15 (m, 1 H), -0.08 (m, 1 H), -0.22 (m, 1 H) ppm. ^{13}**C NMR (75 MHz, $CDCl_3$)** δ 143.90, 143.32, 134.80, 130.27, 129.89, 127.08, 125.40, 125.35, 121.63, 115.26, 47.87, 39.56, 24.33, 21.59, 20.40, 13.77, 2.95 ppm. **HRMS (EI)** calc. for [$C_{23}H_{22}F_3NO_2S$]: 433.1323, found: 433.1320.

1-(4-tert-butylphenyl)-6-cyclopropyl-3-tosyl-3-azabicyclo[4.1.0]hept-4-ene (8B)
^{1}**H NMR (300 MHz, $CDCl_3$)** δ 7.64 (d, *J* = 7.6 Hz, 2 H), 7.27 – 7.32 (m, 4 H), 7.18 (d, *J* = 7.5 Hz, 2 H), 6.38 (d, *J* = 7.9 Hz, 1 H), 5.30 (d, *J* = 8.0 Hz, 1 H), 3.96 (d, *J* = 11.4 Hz, 1 H), 2.97 (d, *J* = 11.4 Hz, 1 H), 2.43 (s, 3 H), 1.30 (s, 9 H), 1.00 (br s, 1 H), 0.95 (br s, 1 H), 0.42 (m, 2 H), 0.14 (d, *J* = 4.1 Hz, 1 H), -0.16 - -0.07 (m, 2H) ppm. ^{13}**C NMR (75 MHz, $CDCl_3$)** δ 149.88, 143.66, 136.01, 134.98, 129.79, 129.48, 127.09, 125.15, 121.15, 115.47, 48.04, 39.69, 34.47, 31.35, 24.03, 21.58, 20.74, 13.83, 2.78 ppm. **HRMS (EI)** calc. for [$C_{26}H_{31}NO_2S$]: 421.2076, found: 421.2074.

6-cyclopropyl-1-m-tolyl-3-tosyl-3-azabicyclo[4.1.0]hept-4-ene (9B)
^{1}**H NMR (300 MHz, $CDCl_3$)** δ 7.80 (d, *J* = 8.2 Hz, 2 H), 7.46 (d, *J* = 8.0 Hz, 2 H), 7.25 – 7.35 (m, 2 H), 7.21 (t, *J* = 7.9 Hz, 2 H), 6.55 (d, *J* = 8.1 Hz , 1 H), 5.48 (d, *J* = 8.1 Hz, 1 H), 4.12 (d, *J* = 11.5 Hz, 1 H), 3.14 (d, *J* = 11.5 Hz, 1 H), 2.58 (s, 3 H), 2.47 (s, 3 H) 1.15 (d, *J* = 4.8 Hz , 1 H), 1.09 (d, *J* = 4.7 Hz, 1 H), 0.61 – 0.70 (m, 1 H), 0.49 – 0.58 (m, 1 H), 0.29 (m, 1 H) 0.06 – 0.14 (m, 1 H), -0.03 - 0.04 (m, 1 H) ppm. ^{13}**C NMR (75 MHz, $CDCl_3$)** δ 143.75, 139.13, 137.93, 134.99, 130.63, 129.85, 128.23, 127.81, 127.10, 126.96, 121.22, 115.64, 48.17, 40.09, 24.11, 21.60, 21.43, 20.66, 13.89, 2.87 ppm. **HRMS (EI)** calc. for [$C_{23}H_{25}NO_2S$]: 379.1606, found: 379.1606.

6-cyclopropyl-1-(3-methoxyphenyl)-3-tosyl-3-azabicyclo[4.1.0]hept-4-ene (10B)
^{1}**H NMR (300 MHz, $CDCl_3$)** δ 7.78 (d, *J* = 8.1 Hz, 2 H), 7.45 (d, *J* = 8.1 Hz, 2 H), 7.34 (t, *J* = 7.9Hz, 1 H), 7.00 (d, *J* = 7.6 Hz, 1 H), 6.92 (dd, *J* = 3.6, 11.8 Hz, 2 H), 6.54 (d, *J* = 8.1 Hz, 1 H), 5.46 (d, *J* = 8.1 Hz, 1 H), 4.10 (d, *J* = 11.5 Hz, 1 H), 3.91 (s, 3 H), 3.13 (d, *J* = 11.5 Hz, 1 H), 2.56 (s, 3 H), 1.14 (d, *J* = 4.8 Hz, 1 H), 1.08 (d, *J* = 4.8 Hz, 1 H), 0.61 – 0.70 (m, 1 H), 0.48 – 0.57 (m, 1 H), 0.27 (m, 1 H), 0.07 – 0.12 (m, 1 H), -0.03 - 0.04 (m, 1 H) ppm. ^{13}**C NMR (75 MHz, $CDCl_3$)** δ 159.52, 143.77, 140.76, 134.92, 129.85, 129.35, 127.07, 122.29, 121.29, 115.79, 115.57, 112.24, 55.24, 48.03, 40.10, 24.25, 21.59, 20.68, 13.84, 2.86 ppm.. **HRMS (EI)** calc. for [$C_{23}H_{25}NO_3S$]: 395.1555, found: 395.1558.

1-(3-chlorophenyl)-6-cyclopropyl-3-tosyl-3-azabicyclo[4.1.0]hept-4-ene (11B)
^{1}**H NMR (300 MHz, $CDCl_3$)** δ 7.64 (d, *J* = 8.2 Hz, 2 H), 7.23 – 7.34(m, 4 H), 7.16 – 7.25 (m, 2 H), 6.40 (d, *J* = 8.1 Hz, 1 H), 5.34 (d, *J* = 8.1 Hz, 1 H), 3.94 (d, *J* = 11.5 Hz, 1 H), 2.97 (d, *J* = 11.5 Hz, 1 H), 2.44 (s, 3 H), 1.00 (d, *J* = 5.0 Hz, 1 H), 0.92 (d, *J* = 5.0 Hz, 1 H), 0.47 – 0.56 (m, 1 H), 0.37 – 0.46 (m, 1 H), 0.14 (dt, *J* = 5.4, 9.6 Hz, 1 H), -0.05 (m, 1 H), -0.20 (m, 1 H) ppm. ^{13}**C NMR (75 MHz, $CDCl_3$)** δ 143.86, 141.29, 134.86, 134.04, 130.01, 129.90, 129.70, 128.13, 127.32, 127.05, 121.45, 115.42, 47.84, 39.64, 24.31, 21.60, 20.43, 13.73, 2.92 ppm. **HRMS (EI)** calc. for [$C_{22}H_{22}NO_2SCl$]: 399.1060, found: 399.1062.

6-cyclopropyl-1-(3,5-dimethylphenyl)-3-tosyl-3-azabicyclo[4.1.0]hept-4-ene (12B)
^{1}**H NMR (300 MHz, $CDCl_3$)** δ 7.64 (d, *J* = 8.2 Hz, 2 H), 7.30 (d, *J* = 8.1 Hz, 2 H), 6.87 (d, *J* = 5.0 Hz, 3 H), 6.38 (d, *J* = 8.1H z, 1 H), 5.30 (d, *J* = 8.1 Hz, 1 H), 3.94 (d, *J* = 11.4 Hz, 1 H), 2.96 (d, *J* = 11.5 Hz, 1 H), 2.43 (s, 3 H), 2.27 (s, 6 H), 1.00 (d, *J* = 4.8 Hz, 1 H), 0.92 (d, *J* = 4.8 Hz, 1 H), 0.43 – 0.51 (m, 1 H), 0.33 – 0.42 (m, 1 H), 0.14 (m, 1 H), -0.07 – -0.01 (m, 1 H), -0.16 - -0.09 (m, 1 H) ppm. ^{13}**C NMR (75 MHz, $CDCl_3$)** δ 143.64, 139.06, 137.73, 135.07, 129.79, 128.65, 127.69, 127.10, 121.16, 115.46, 48.17, 40.09, 24.01, 21.57, 21.25, 20.78, 13.88, 2.75 ppm. **HRMS (EI)** calc. for [$C_{24}H_{27}NO_2S$]: 393.1763, found: 393.1759.

6-cyclopropyl-1-(naphthalen-1-yl)-3-tosyl-3-azabicyclo[4.1.0]hept-4-ene (13B)
^{1}**H NMR (300 MHz, $CDCl_3$)** δ 7.85 (d, *J* = 9.5 Hz, 2 H), 7.76 (m, 2 H), 7.63 (d, *J* = 8.1 Hz, 2 H), 7.41 (d, *J* = 5.1 Hz, 3 H), 7.28 (d, *J* = 8.2 Hz, 2 H), 6.56 (d, *J* = 8.2 Hz, 1 H), 5.42 (d, *J* = 8.1 Hz, 1 H), 3.88 (d, *J* = 11.9 Hz, 1 H), 3.40 (d, *J* = 11.9 Hz, 1 H), 2.42 (s, 3 H), 1.07 (s, 2 H), 0.39 - 0.43 (m, 1 H), 0.07 – 0.23 (m, 2 H), -0.41 - -0.24 (m, 2 H) ppm. ^{13}**C NMR (75 MHz, $CDCl_3$)** δ 143.79, 136.58, 134.95, 134.00, 132.05, 129.87, 128.70, 128.42, 128.07, 127.19, 127.06, 125.87, 125.68, 125.57, 121.35, 114.34, 46.49, 38.18, 25.26, 21.61, 21.14, 13.60, 3.12, 2.63 ppm. **HRMS (EI)** calc. for [$C_{26}H_{25}NO_2S$]: 415.1606, found: 415.1608.

6-cyclopropyl-1-mesityl-3-tosyl-3-azabicyclo[4.1.0]hept-4-ene (14B)
^{1}H NMR (300 MHz, CDCl$_3$) δ 7.56 (d, *J* = 8.1 Hz, 2 H), 7.22 (d, *J* = 8.0 Hz, 2 H), 6.72 (s, 2 H), 6.35 (d, *J* = 8.1 Hz, 1 H), 5.16 (d, *J* = 8.1 Hz, 1 H), 3.74 (d, *J* = 12.0 Hz, 1 H), 2.84 (d, *J* = 12.0 Hz, 1 H), 2.34 (s, 3 H), 2.17 (d, *J* = 12.1 Hz, 6 H), 2.13 (s, 3 H), 0.97 (d, *J* = 5.0 Hz, 1 H), 0.58 (d, *J* = 4.7Hz, 1 H), 0.42 – 0.51 (m, 1 H), 0.25 – 0.34 (m, 1 H), 0.07 – 0.16 (m, 1 H), -0.07 (dd, *J* = 4.4, 9.8 Hz, 1 H), -0.31 (m, 1 H) ppm. **^{13}C NMR (75 MHz, CDCl$_3$)** δ 143.73, 138.67, 138.55, 136.45, 135.02, 129.78, 129.56, 129.08, 127.07, 121.11, 114.55, 44.73, 37.46, 25.27, 22.77, 21.59, 20.85, 20.38, 13.11, 3.65, 1.84 ppm. **HRMS (EI)** calc. for [$C_{25}H_{29}NO_2S$]: 407.1919, found: 407.1921.

6-cyclopropyl-4-methyl-1-phenyl-3-tosyl-3-azabicyclo[4.1.0]hept-4-ene (15B)
^{1}H NMR (300 MHz, CDCl$_3$) δ 7.61 (d, *J* = 8.2 Hz, 2 H), 7.22 (m, 7 H), 5.34 (s, 1 H), 4.18 (d, *J* = 12.8 Hz, 1 H), 3.11 (d, *J* = 12.8 Hz, 1 H), 2.34 (s, 3 H), 1.86 (s, 3 H), 1.07 (d, *J* = 5.3 Hz, 1 H), 0.88 (d, *J* = 5.2 Hz, 1 H), 0.45 – 0.54 (m, 1 H), 0.28 – 0.37 (m, 1 H), 0.07 (m, 1 H), -0.15 (m, 1 H), -0.27 - -0.21 (m, 1 H) ppm. **^{13}C NMR (75 MHz, CDCl$_3$)** δ 143.10, 139.16, 138.73, 130.03, 129.88, 129.63, 128.31, 126.89, 126.62, 120.52, 50.46, 41.67, 24.92, 21.51, 20.77, 18.76, 14.21, 2.98 ppm. **HRMS (EI)** calc. for [$C_{23}H_{25}NO_2S$]: 379.1606, found: 379.1608.

6-cyclopropyl-1-phenyl-3-oxabicyclo[4.1.0]hept-4-ene (16B)
^{1}H NMR (300 MHz, CDCl$_3$) δ 7.24 – 7.39 (m, 5 H), 6.23 (d, *J* = 5.9 Hz, 1 H), 5.17 (d, *J* = 5.9 Hz, 1 H), 4.07 (d, *J* = 10.5 Hz, 1 H), 3.80 (d, *J* = 10.5 Hz, 1 H), 1.31 (d, *J* = 4.5 Hz, 1 H), 1.10 (d, *J* = 4.4 Hz, 1 H), 0.52 (m, 1 H), 0.39 – 0.43 (m, 1 H), 0.21 (m , 1 H), -0.10 - -0.01 (m, 2 H) ppm. **^{13}C NMR (75 MHz, CDCl$_3$)** δ 141.55, 138.85, 130.11, 128.27, 126.80, 109.74, 68.47, 38.76, 23.11, 20.61, 13.77, 2.65, 2.47 ppm. **HRMS (EI)** calc. for [$C_{15}H_{16}O$]: 212.1201, found 212.1200.

8a-phenyl-2-tosyl-1,2,6,7,8,8a-hexahydroisoquinoline (2C)
^{1}H NMR (300 MHz, CDCl$_3$) δ 7.42 (d, *J* = 8.1 Hz, 2 H), 7.15 – 7.17 (m, 7 H), 6.44 (d, *J* = 8.0 Hz, 1 H), 5.71 (t, *J* = 3.7 Hz, 1 H), 5.49 (d, *J* = 8.1 Hz, 1 H), 4.26 (d, *J* = 11.6 Hz, 1 H), 2.83 (d, *J* = 11.6 Hz, 1 H), 2.39 (s, 3 H), 2.14 (dd, *J* = 3.5, 7.7 Hz, 2 H), 1.82 – 1.87 (m, 1 H), 1.41 – 1.51 (m, 2 H), 1.26 – 1.27 (d, *J* = 4.5 Hz, 1 H) ppm. **^{13}C NMR (75 MHz, CDCl$_3$)** δ 143.42, 142.53, 135.25, 132.73, 129.61, 127.97, 127.40, 126.89, 126.21, 124.43, 123.80, 109.76, 54.28, 42.17, 34.81, 25.52, 21.52, 17.51 ppm. **HRMS (EI)** calc. for [$C_{22}H_{23}NO_2S$]: 365.1450, found: 365.1446.

8a-p-tolyl-2-tosyl-1,2,6,7,8,8a-hexahydroisoquinoline (3C)
^{1}H NMR (300 MHz, CDCl$_3$) δ 7.42 (d, *J* = 8.1 Hz, 2 H), 7.16 (d, *J* = 8.0 Hz, 2 H), 7.04 (d, *J* = 8.0 Hz, 2 H), 6.96 (d, *J* = 8.1 Hz, 2 H), 6.45 (d, *J* = 8.0 Hz, 1 H), 5.69 (t, *J* = 3. 6 Hz, 1 H), 5.48 (d, *J* = 8.0 Hz, 1 H), 4.23 (d, *J* = 11.6 Hz, 1 H), 2.82 (d, *J* = 11.6 Hz, 1 H), 2.39 (s, 3 H), 2.29 (s, 3 H), 2.11 – 2.14 (m, 2 H), 1.82 (td, *J* = 3.6, 5.5 Hz, 1 H), 1.39 – 1.48 (m, 2 H), 1.25 (t, *J* = 11.3 Hz, 1 H) ppm. **^{13}C NMR (75 MHz, CDCl$_3$)** δ 143.29, 139.46, 135.68, 135.39, 132.88, 129.51, 128.64, 127.24, 126.88, 124.28, 123.72, 109.78, 54.34, 41.82, 34.77, 25.53, 21.56, 20.99, 17.55 ppm. **HRMS (EI)** calc. for [$C_{23}H_{25}NO_2S$]: 379.1606, found: 379.1604.

8a-(4-methoxyphenyl)-2-tosyl-1,2,6,7,8,8a-hexahydroisoquinoline (4C)
^{1}H NMR (300 MHz, CDCl$_3$) δ 7.35 (d, *J* = 8.3 Hz, 2 H), 7.08 (d, *J* = 8.0 Hz, 2 H), 6.98 (d, *J* = 8.9 Hz, 2 H), 6.61 (d, *J* = 8.9 Hz, 2 H), 6.40 (d, *J* = 8.0 Hz, 1 H), 5.61 (t, *J* = 3.8 Hz, 1 H), 5.41 (d, *J* = 8.1 Hz, 1 H), 4.12 (d, *J* = 11.6 Hz, 1 H), 3.70 (s, 3 H), 2.76 (d, *J* = 11.7 Hz, 1 H), 2.32 (s, 3 H), 2.06 (dd, *J* = 3.9, 8.1 Hz, 2 H), 1.07 – 1.75 (m, 1 H), 1.32 – 1.43 (m, 2 H), 1.19 – 1.20 (m, 1 H) ppm. **^{13}C NMR (75 MHz, CDCl$_3$)** δ 157.96, 143.34, 135.47, 134.36, 132.96, 129.53, 128.39, 126.82, 124.28, 123.71, 113.25, 109.77, 55.08, 54.44, 41.51, 34.76, 25.54, 21.50, 17.52 ppm. **HRMS (EI)** calc. for [$C_{23}H_{25}NO_3S$]: 395.1555, found: 395.1552.

8a-(4-chlorophenyl)-2-tosyl-1,2,6,7,8,8a-hexahydroisoquinoline (5C)
^{1}H NMR (300 MHz, CDCl$_3$) δ 7.39 (d, *J* = 8.3 Hz, 2 H), 7.16 (d, *J* = 8.1 Hz, 2 H), 7.00 – 7.06 (m, 4 H), 6.53 (d, *J* = 8.0 Hz, 1 H), 5.72 (t, *J* = 3.7 Hz, 1 H), 5.50 (d, *J* = 8.0 Hz, 1 H), 4.16 (d, *J* = 12.0 Hz, 1 H), 2.93 (d, *J* = 12.0 Hz, 1 H), 2.43 (s, 3 H), 2.11 – 2.15 (m, 2 H), 1.79 – 1.84 (m, 1 H), 1.43 – 1.52 (m, 2 H), 1.10 – 1.19 (m, 1 H) ppm. **^{13}C NMR (75 MHz, CDCl$_3$)** δ 143.62, 141.16, 135.56, 132.34, 132.17, 129.56, 128.83, 127.96, 126.64, 124.72, 123.91, 109.39, 54.35, 41.85, 34.50, 25.46, 21.61, 17.49 ppm. **HRMS (EI)** calc. for [$C_{22}H_{22}NO_2SCl$]: 399.1060, found: 399.1062.

8a-(4-fluorophenyl)-2-tosyl-1,2,6,7,8,8a-hexahydroisoquinoline (6C)
^{1}H NMR (300 MHz, $CDCl_3$) δ 7.34 (d, *J* = 8.2 Hz, 2 H), 7.08 (d, *J* = 8.1 Hz, 2 H), 7.00 (dd, *J* = 5.4, 8.7 Hz, 2 H), 6.71 (t, *J* = 8.7 Hz, 2 H), 6.43 (d, *J* = 8.0 Hz, 1 H), 5.62 (t, *J* = 3.7 Hz, 1 H), 5.42 (d, *J* = 8.0 Hz, 1 H), 4.10 (d, *J* = 11.7 Hz, 1 H), 2.80 (d, *J* = 11.8 Hz, 1 H), 2.32 (s, 3 H), 2.04 – 2.07 (m, 2 H), 1.72 – 1.76 (m, 1 H), 1.34 – 1.43 (m, 2 H), 1.05 – 1.18 (m, 1 H) ppm. **^{13}C NMR (75 MHz, $CDCl_3$)** δ 143.56, 135.52, 132.63, 129.58, 129.03, 128.92, 126.73, 124.57, 123.88, 114.77, 114.49, 109.55, 54.47, 41.72, 34.69, 25.47, 21.45, 17.45 ppm. **HRMS (EI)** calc. for [$C_{22}H_{22}NO_2FS$]: 383.1355, found: 383.1353.

2-tosyl-8a-(4-(trifluoromethyl)phenyl)-1,2,6,7,8,8a-hexahydroisoquinoline (7C)
^{1}H NMR (300 MHz, $CDCl_3$) δ 7.32 (d, *J* = 8.3, Hz, 2 H), 7.28 (d, *J* = 8.8 Hz, 2 H), 7.16 (d, *J* = 8.3 Hz, 2 H), 7.06 (d, *J* = 8.1 Hz, 2 H), 6.44 (d, *J* = 8.0 Hz, 1 H), 5.67 (t, *J* = 3.6 Hz, 1 H), 5.43 (d, *J* = 8.1 Hz, 1 H), 4.16 (d, *J* = 11.9 Hz, 1 H), 2.87 (d, *J* = 11.9 Hz, 1 H), 2.29 (s, 3 H), 2.05 – 2.08 (m, 2 H), 1.75 – 1.79 (m, 1 H), 1.32 - 1.51 (m, 2 H), 0.98 – 1.11 (m, 1 H) ppm. **^{13}C NMR (75 MHz, $CDCl_3$)** δ 143.71, 135.40, 132.09, 129.58, 127.82, 126.70, 124.91, 124.83, 124.78, 124.73, 124.00, 109.24, 54.14, 42.30, 34.58, 25.40, 21.36, 17.44 ppm. **HRMS (EI)** calc. for [$C_{23}H_{22}F_3NO_2S$]: 433.1323, found: 133.1326.

8a-(4-tert-butylphenyl)-2-tosyl-1,2,6,7,8,8a-hexahydroisoquinoline (8C)
^{1}H NMR (300 MHz, $CDCl_3$) δ 7.41 (d, *J* = 8.2 Hz, 2 H), 7.12 – 7.16 (m, 4 H), 7.03 (d, *J* = 8.4 Hz, 2 H), 6.33 (d, *J* = 8.0 Hz, 1 H), 5.62 (t, *J* = 3.6 Hz, 1 H), 5.40 (d, *J* = 8.1 Hz, 1 H), 4.21 (d, *J* = 11.3 Hz, 1 H), 2.69 (d, *J* = 11.4 Hz, 1 H), 2.32 (s, 3 H), 2.05 – 2.07 (m, 2 H), 1.70 – 1.75 (m, 1 H), 1.32 – 1.43 (m, 2 H), 1.23 (s, 9 H), 1.23 (m, 1 H) ppm. **^{13}C NMR (75 MHz, $CDCl_3$)** δ 148.82, 143.37, 139.30, 135.08, 132.88, 129.62, 127.09, 126.98, 124.81, 124.15, 123.70, 109.76, 71.35, 54.00, 42.63, 41.74, 35.02, 34.27, 31.43, 25.53, 21.57, 17.52 ppm. **HRMS (EI)** calc. for [$C_{26}H_{31}NO_2S$]: 421.2076, found: 421.2079.

8a-m-tolyl-2-tosyl-1,2,6,7,8,8a-hexahydroisoquinoline (9C)
^{1}H NMR (300 MHz, $CDCl_3$) δ 7.33 (d, *J* = 8.2 Hz, 2 H), 7.07 (d, *J* = 8.1 Hz, 2 H), 6.98 (d, *J* = 7.7 Hz, 1 H), 685 – 6.90 (m, 3 H), 6.39 (d, *J* = 8.0 Hz, 1 H), 5.62 (t, *J* = 3.6 H, 1 H), 5.43 (d, *J* = 8.1 Hz, 1 H), 4.16 (d, *J* = 11.6 Hz, 1 H), 2.77 (d, *J* = 11.6 Hz, 1 H), 2.31 (s, 3 H), 2.17 (s, 3 H), 2.04 – 2.07 (m, 2 H), 1.75 – 1.80 (m, 1 H), 1.32 – 1.42 (m, 2 H), 1.13 – 1.20 (m, 1 H) ppm. **^{13}C NMR (75 MHz, $CDCl_3$)** δ 143.27, 142.57, 137.21, 135.38, 132.83, 129.51, 128.01, 127.79, 127.02, 126.79, 124.60, 124.40, 123.70, 109.90, 54.48, 42.07, 34.79, 25.55, 21.67, 21.52, 17.58 ppm. **HRMS (EI)** calc. for [$C_{23}H_{25}NO_2S$]: 379.1606, found: 379.1607.

8a-(3-methoxyphenyl)-2-tosyl-1,2,6,7,8,8a-hexahydroisoquinoline (10C)
^{1}H NMR (300 MHz, $CDCl_3$) δ 7.35 (d, *J* = 8.2 Hz, 2 H), 7.00 – 7.09 (m, 3 H), 6.71 (d, *J* = 7.5 Hz, 1 H), 6.59 (d, *J* = 7.5 Hz, 2 H), 6.40 (d, *J* = 8.0 Hz, 1 H), 5.62 (t, *J* = 3.5 Hz, 1 H), 5.42 (d, *J* = 8.0 Hz, 1 H), 4.16 (d, *J* = 11.6 Hz, 1 H), 3.66 (s, 3 H), 2.76 (d, *J* = 11.6 Hz, 1 H), 2.30 (s, 3 H), 2.05 – 2.06 (m, 2 H), 1.79 (dd, *J* = 3.5, 12.7 Hz, 1 H), 1.32 – 1.41 (m, 2 H), 1.19 (dd, *J* = 8.7, 11.6 Hz, 1 H) ppm. **^{13}C NMR (75 MHz, $CDCl_3$)** δ 159.14, 144.40, 143.36, 135.31, 132.74, 129.56, 128.88, 126.81, 124.50, 123.74, 119.74, 114.04, 111.13, 109.81, 55.03, 54.37, 42.22, 34.67, 25.51, 21.53, 17.55 ppm. **HRMS (EI)** calc. for [$C_{23}H_{25}NO_3S$]: 395.1555, found: 395.1559.

8a-(3-chlorophenyl)-2-tosyl-1,2,6,7,8,8a-hexahydroisoquinoline (11C)
^{1}H NMR (300 MHz, $CDCl_3$) δ 7.41 (d, *J* = 8.2 Hz, 2 H), 7.16 (d, *J* = 8.1 Hz, 2 H), 7.03 – 7.07 (m, 4 H), 6.52 (d, *J* = 8.0 Hz, 1 H), 5.72 (t, *J* = 3.7 Hz, 1 H), 5.51 (d, *J* = 8.1 Hz, 1 H), 4.16 (d, *J* = 11.8 Hz, 1 H), 2.90 (d, *J* = 11.9 Hz, 1 H), 2.39 (s, 3 H), 2.12 – 2.15 (m, 2 H), 1.81 – 1.86 (m, 1 H), 1.42 – 1.52 (m, 2 H), 1.11 – 1.22 (m, 1 H) ppm. **^{13}C NMR (75 MHz, $CDCl_3$)** δ 145.07, 143.52, 135.31, 133.80, 132.14, 129.61, 129.26, 127.90, 126.67, 126.46, 125.49, 124.92, 123.93, 109.51, 54.35, 42.18, 34.55, 25.44, 21.55, 17.49 ppm. **HRMS (EI)** calc. for [$C_{22}H_{22}NO_2SCl$]: 399.1060, found: 399.1062.

8a-(3,5-dimethylphenyl)-2-tosyl-1,2,6,7,8,8a-hexahydroisoquinoline (12C)
^{1}H NMR (300 MHz, $CDCl_3$) δ 7.40 (d, *J* = 8.2 Hz, 2 H), 7.15 (d, *J* = 8.1 Hz, 2 H), 6.74 (m + s, 3 H), 6.51 (d, *J* = 8.0 Hz, 1 H), 5.71 (t, *J* = 3.4 Hz, 1 H), 5.54 (d, *J* = 8.0 Hz, 1 H), 4.24 (d, *J* = 11.7 Hz, 1 H), 2.89 (d, *J* = 11.7 Hz, 1 H), 2.40 (s, 3 H), 2.23 (s, 6 H), 2.14 – 2.17 (m, 2 H), 1.86 – 1.91 (m, 1 H), 1.40 – 1.49 (m, 2 H), 1.30 (br s, 1 H) ppm. **^{13}C NMR (75 MHz, $CDCl_3$)** δ 143.12, 142.62, 136.98, 135.56, 132.96, 129.35, 127.98, 126.68, 125.24, 124.38, 123.60, 110.07, 54.72, 41.98, 34.74, 25.58, 21.52,

17.64, 1.04 ppm. **HRMS (EI)** calc. for [$C_{24}H_{27}NO_2S$]: 393.1763, found: 393.1765.

3-methyl-8a-phenyl-2-tosyl-1,2,6,7,8,8a-hexahydroisoquinoline (15C)
^{1}H NMR (300 MHz, $CDCl_3$) δ 7.21 – 7.27 (m, 5 H), 6.91 – 6.98 (m, 4 H), 5.54 (t, *J* = 3.5 Hz, 1 H), 5.24 (s, 1 H), 4.78 (d, *J* = 12.2 Hz, 1 H), 3.13 (d, *J* = 12.2 Hz, 1 H), 2.26 (s, 3 H), 2.08 – 2.10 (m, 2 H), 1.91 (dd, *J* = 3.1, 12.0 Hz, 1 H), 1.73 (s, 3 H), 1.47 (t, *J* = 5.6 Hz, 2 H), 1.42 (dd, *J* = 6.1, 9.1Hz, 1 H) ppm. **^{13}C NMR (75 MHz, $CDCl_3$)** δ 143.13, 144.00, 138.73, 134.46, 132.83, 129.49, 128.22, 128.16, 126.94, 126.42, 122.38, 111.29, 56.57, 42.75, 34.26, 25.40, 21.43, 20.94, 17.75 ppm. **HRMS (EI)** calc. for [$C_{23}H_{25}NO_2S$]: 379.1606, found: 379.1608.

Table S2. Melting Point of **C** compounds

Entry		Products	Melting Point (℃)
		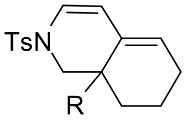 **C**	
		R	
1	**2C**	C_6H_5	136
2	**3C**	4-$CH_3C_6H_4$	116.8
3	**4C**	4-$CH_3OC_6H_4$	97.4
4	**5C**	4-ClC_6H_4	156.3
5	**6C**	4-FC_6H_4	107
6	**7C**	4-$F_3CC_6H_4$	124
7	**8C**	4-tert-butylbenzene	Sticky
8	**9C**	3-$CH_3C_6H_4$	Oil
9	**10C**	3-$CH_3OC_6H_4$	Oil
10	**11C**	3-ClC_6H_4	Oil
11	**12C**	m-xylene	Oil
14	**15C**	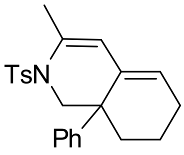	Oil

NMR Spectrums

1A

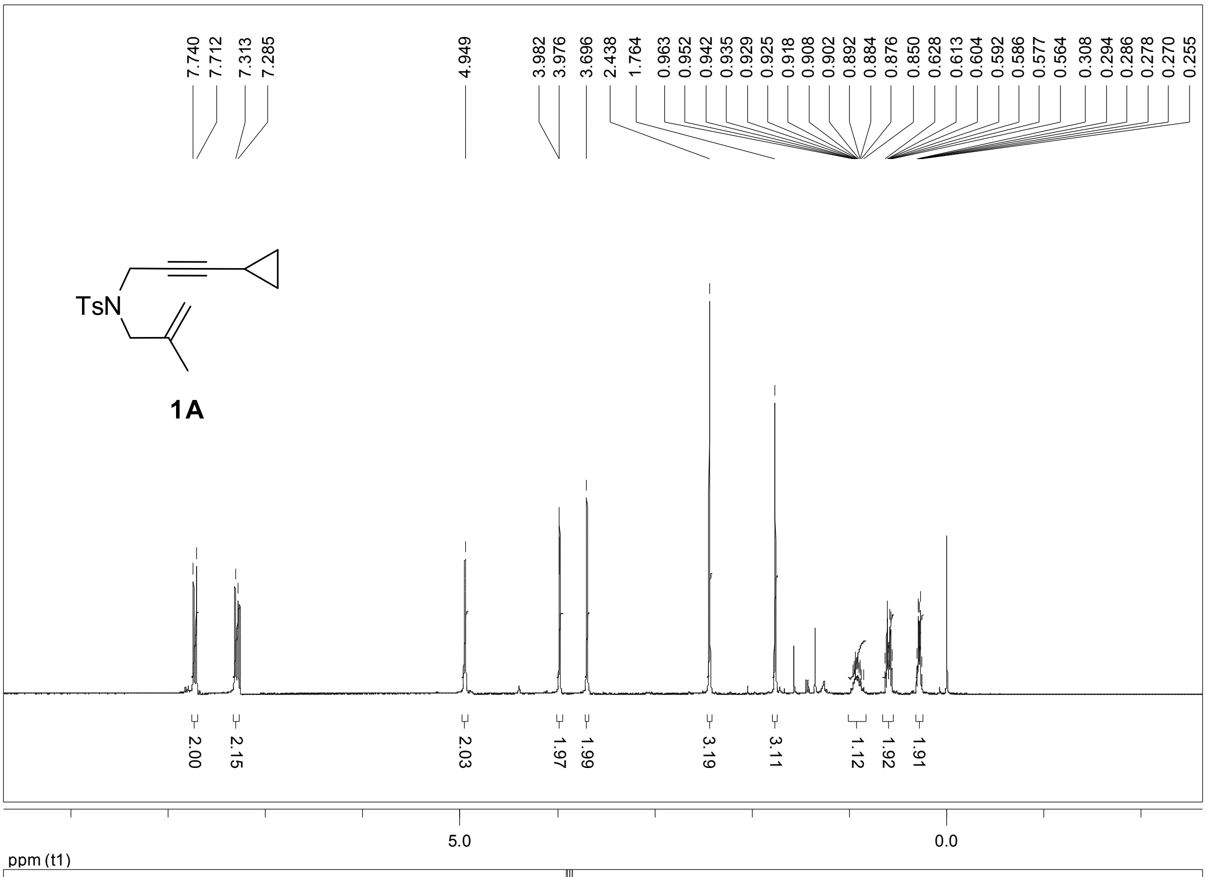

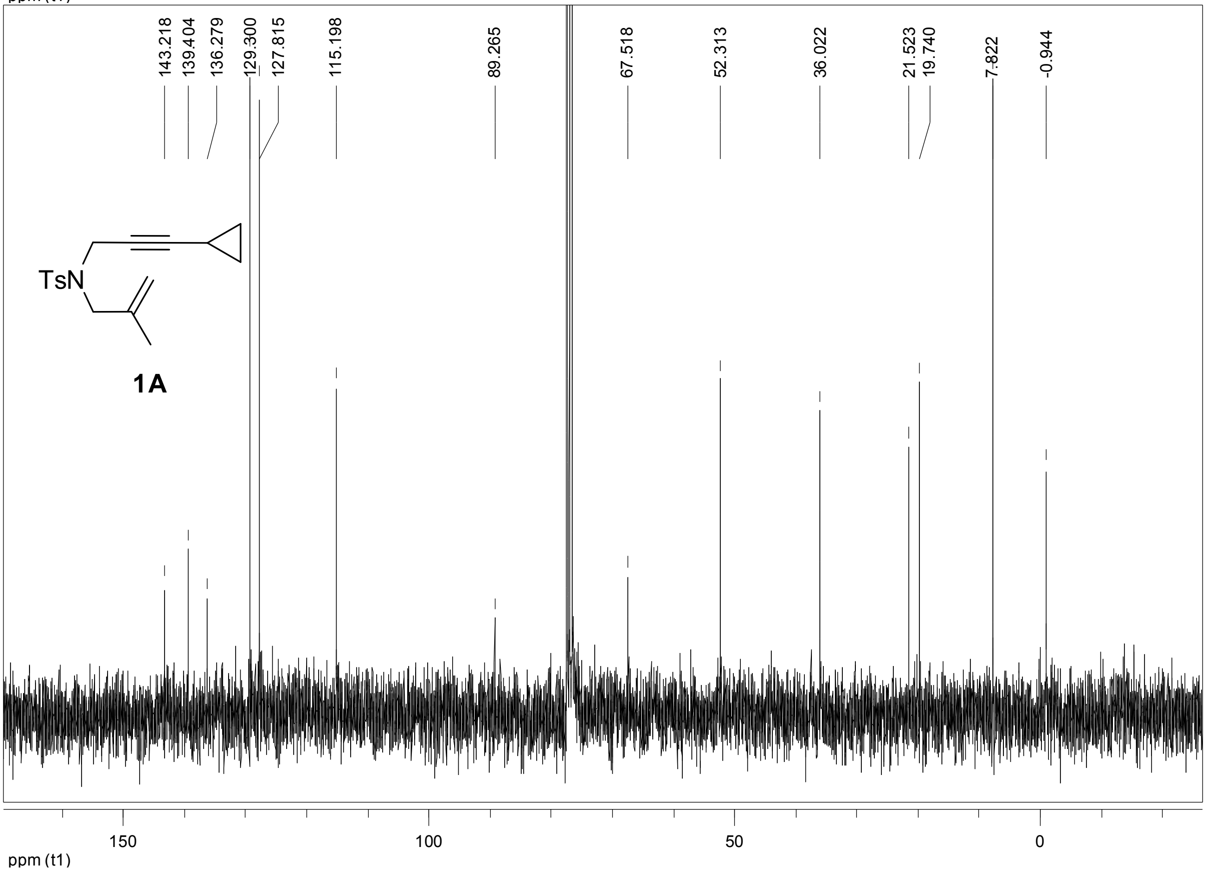

2A

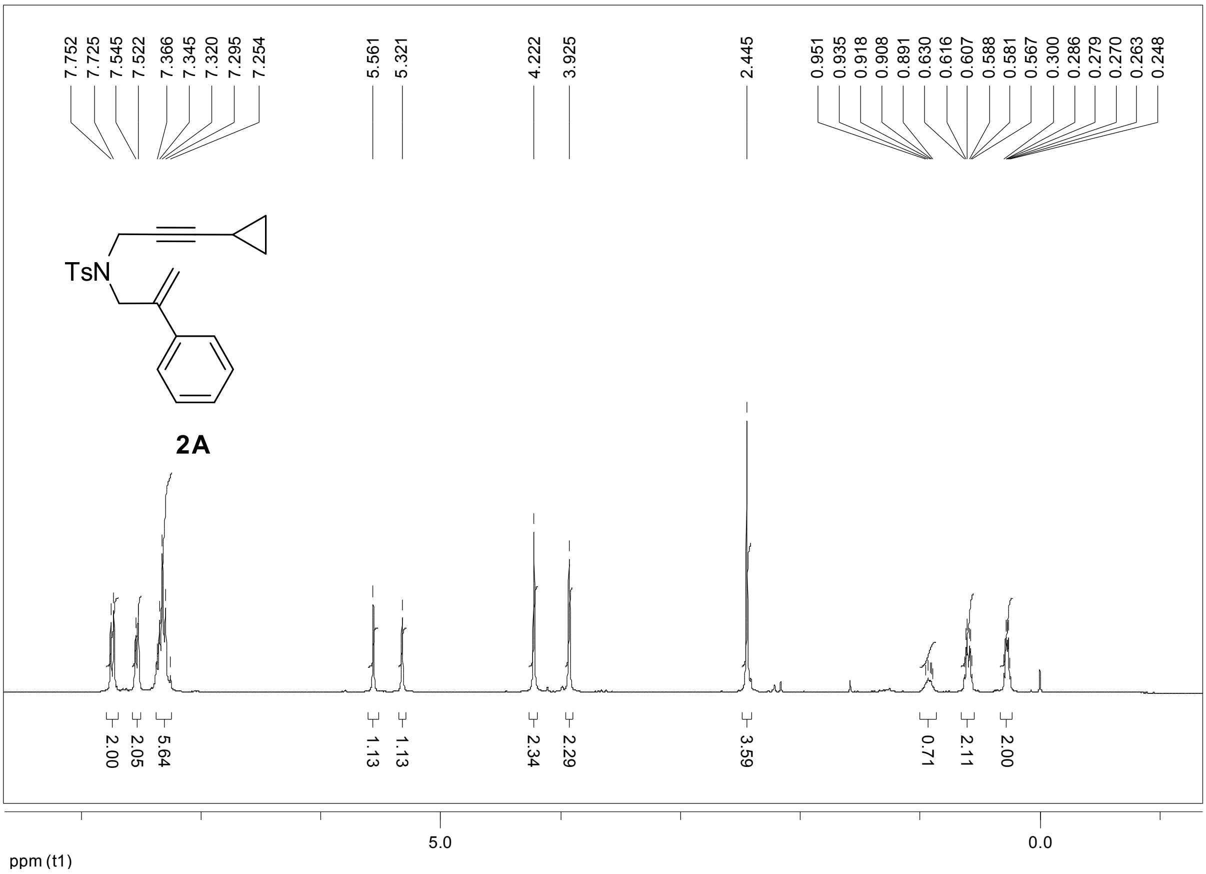
7.752
7.725
7.545
7.522
7.366
7.345
7.320
7.295
7.254
5.561
5.321
4.222
3.925
2.445
0.951
0.935
0.918
0.908
0.891
0.630
0.616
0.607
0.588
0.581
0.567
0.300
0.286
0.279
0.270
0.263
0.248
TsN
2A
2.00
2.05
5.64
1.13
1.13
2.34
2.29
3.59
0.71
2.11
2.00
5.0
0.0
ppm (t1)

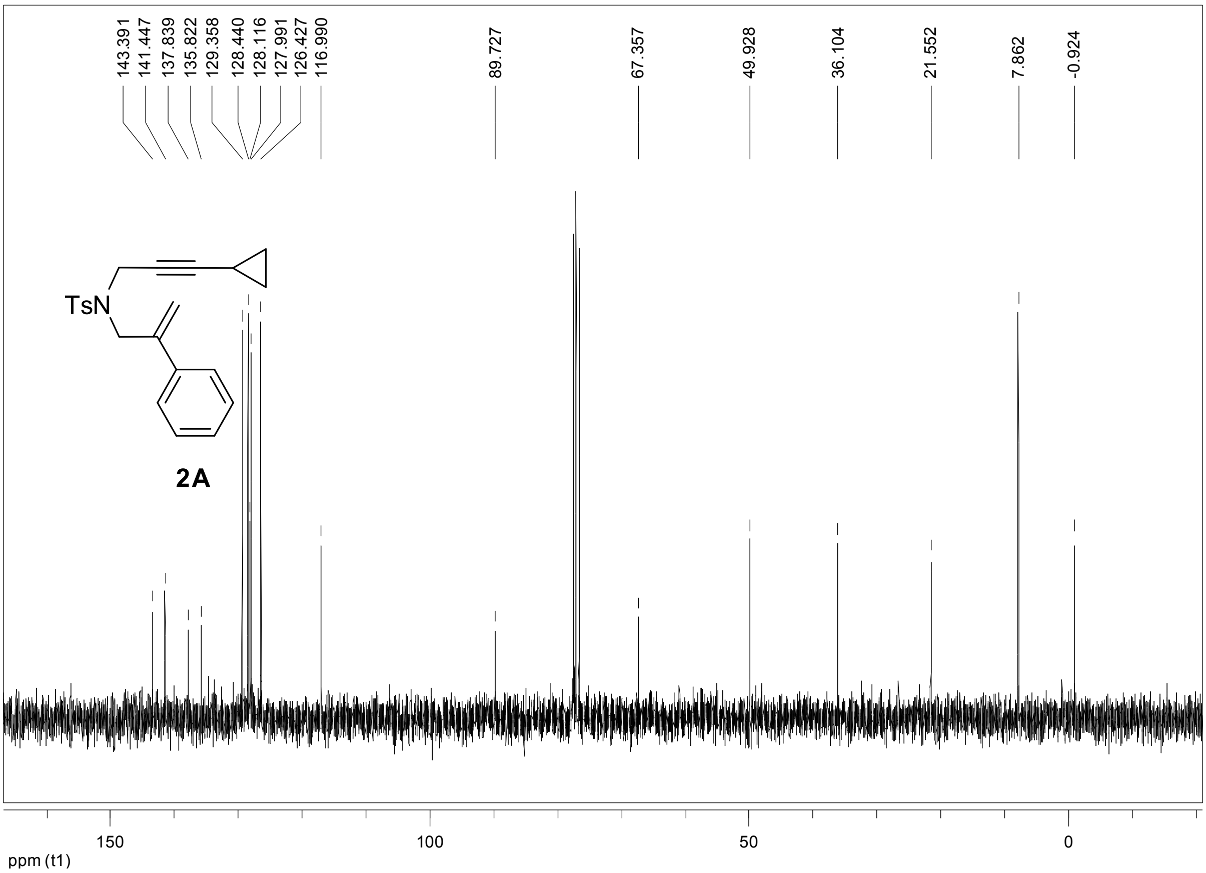
143.391
141.447
137.839
135.822
129.358
128.440
128.116
127.991
126.427
116.990
89.727
67.357
49.928
36.104
21.552
7.862
-0.924
TsN
2A
150
100
50
0
ppm (t1)

3A

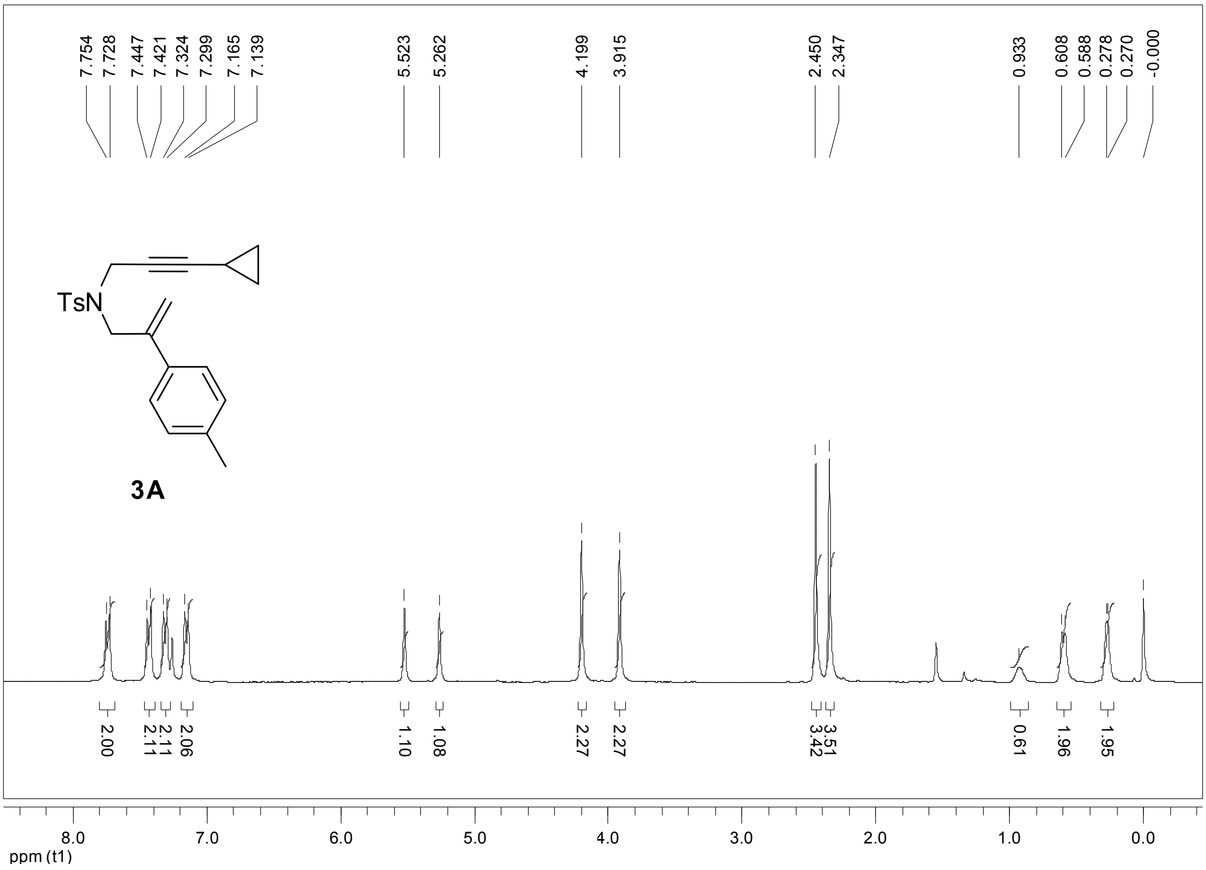
7.754
7.728
7.447
7.421
7.324
7.299
7.165
7.139
5.523
5.262
4.199
3.915
2.450
2.347
0.933
0.608
0.588
0.278
0.270
-0.000
TsN
3A
2.00
2.11
2.11
2.06
1.10
1.08
2.27
2.27
3.42
3.51
0.61
1.96
1.95
8.0
7.0
6.0
5.0
4.0
3.0
2.0
1.0
0.0
ppm (t1)

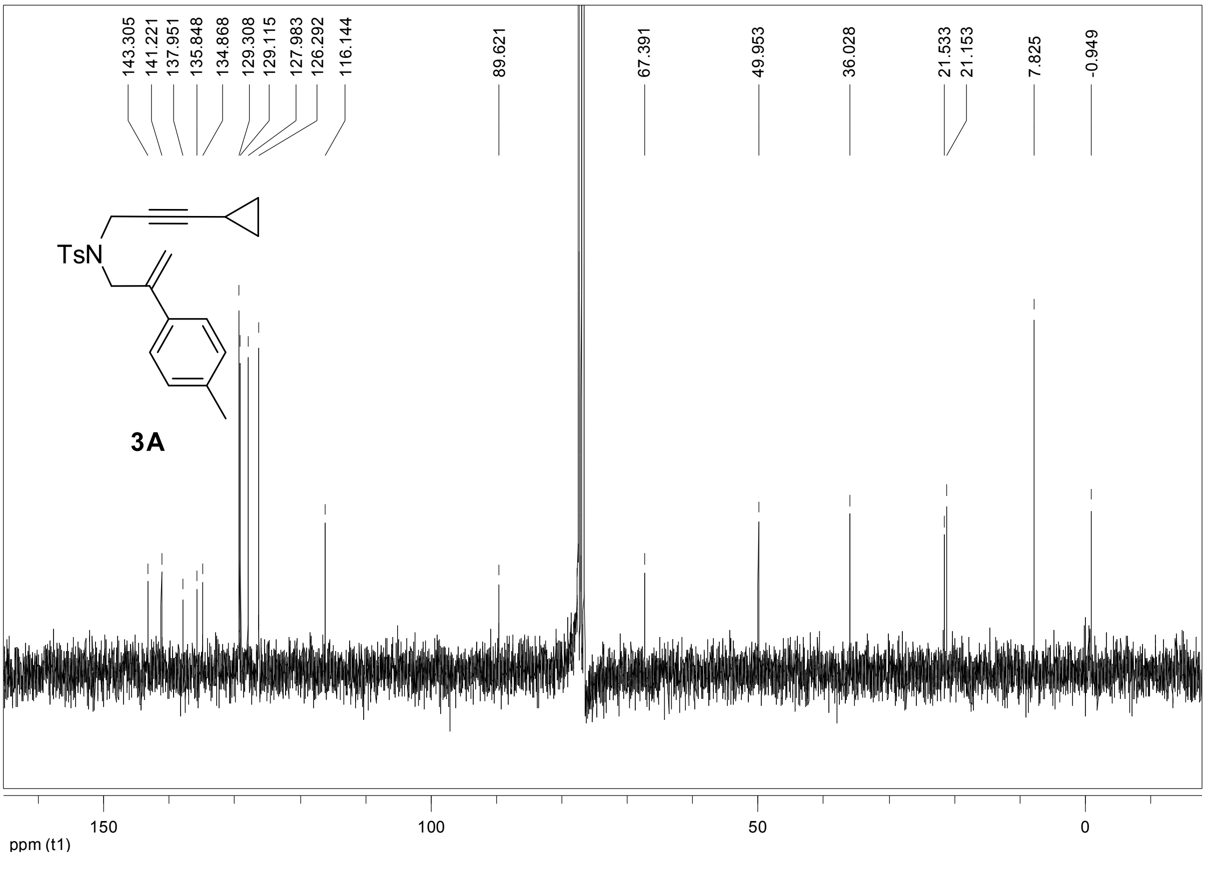
143.305
141.221
137.951
135.848
134.868
129.308
129.115
127.983
126.292
116.144
89.621
67.391
49.953
36.028
21.533
21.153
7.825
-0.949
TsN
3A
150
100
50
0
ppm (t1)

4A

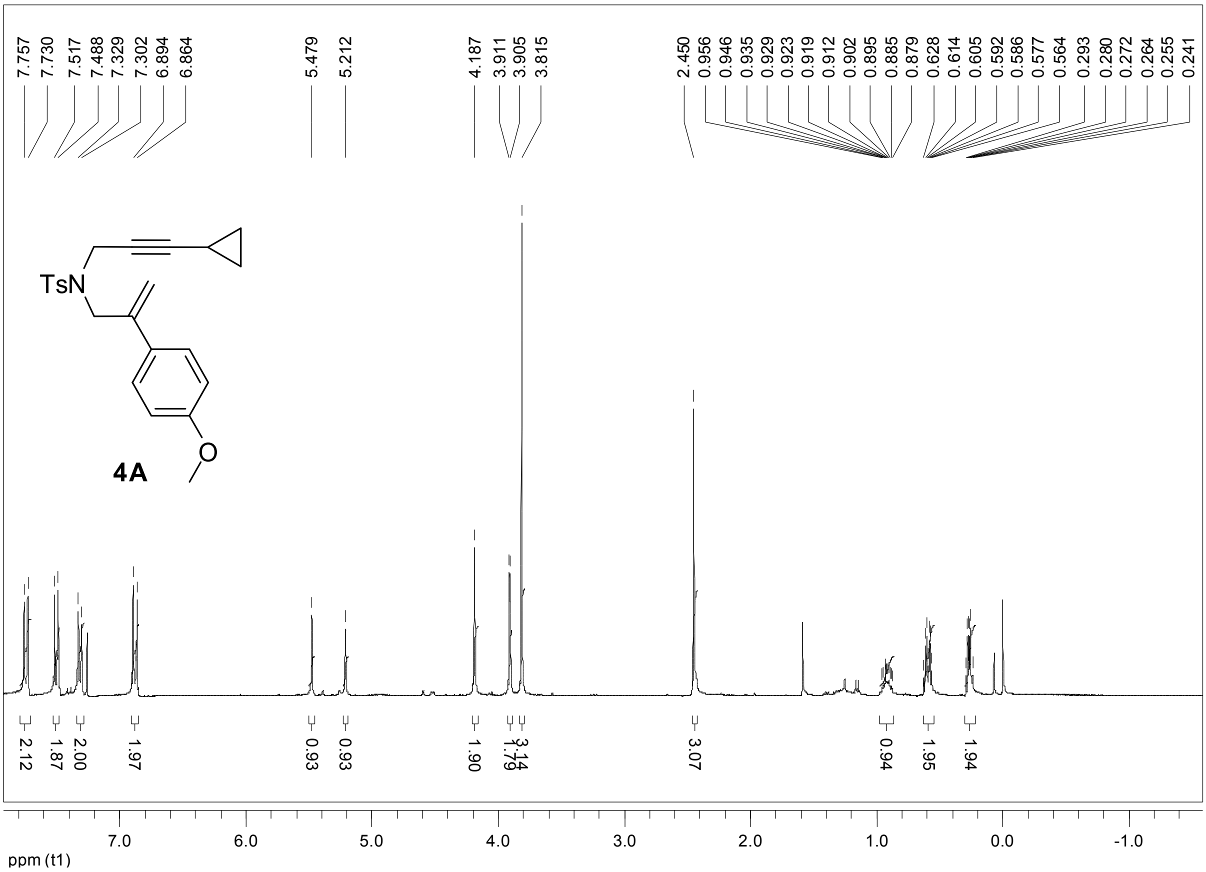
7.757
7.730
7.517
7.488
7.329
7.302
6.894
6.864
5.479
5.212
4.187
3.911
3.905
3.815
2.450
0.956
0.946
0.935
0.929
0.923
0.919
0.912
0.902
0.895
0.885
0.879
0.628
0.614
0.605
0.592
0.586
0.577
0.564
0.293
0.280
0.272
0.264
0.255
0.241
TsN
4A
O
2.12
1.87
2.00
1.97
0.93
0.93
1.90
1.79
3.14
3.07
0.94
1.95
1.94
7.0
6.0
5.0
4.0
3.0
2.0
1.0
0.0
-1.0
ppm (t1)

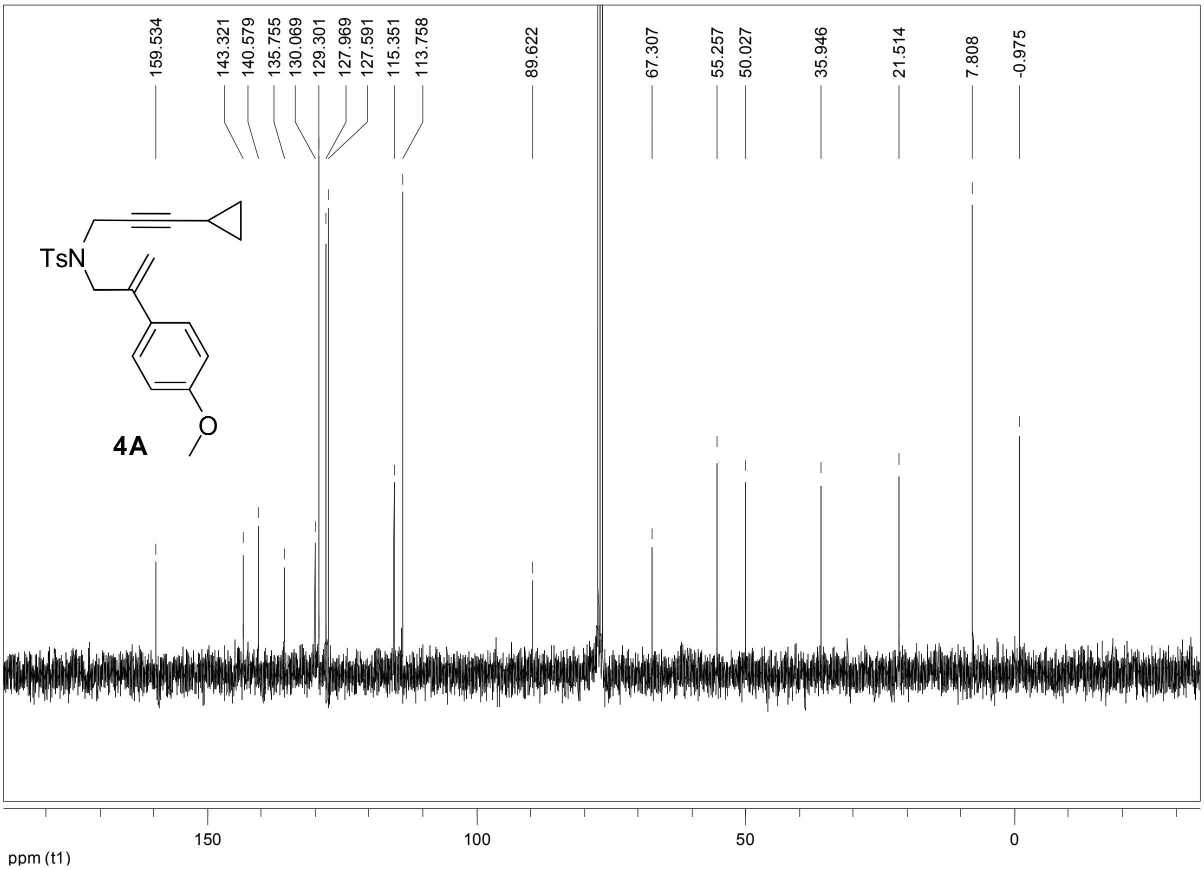
159.534
143.321
140.579
135.755
130.069
129.301
127.969
127.591
115.351
113.758
89.622
67.307
55.257
50.027
35.946
21.514
7.808
-0.975
TsN
4A
O
150
100
50
0
ppm (t1)

5A

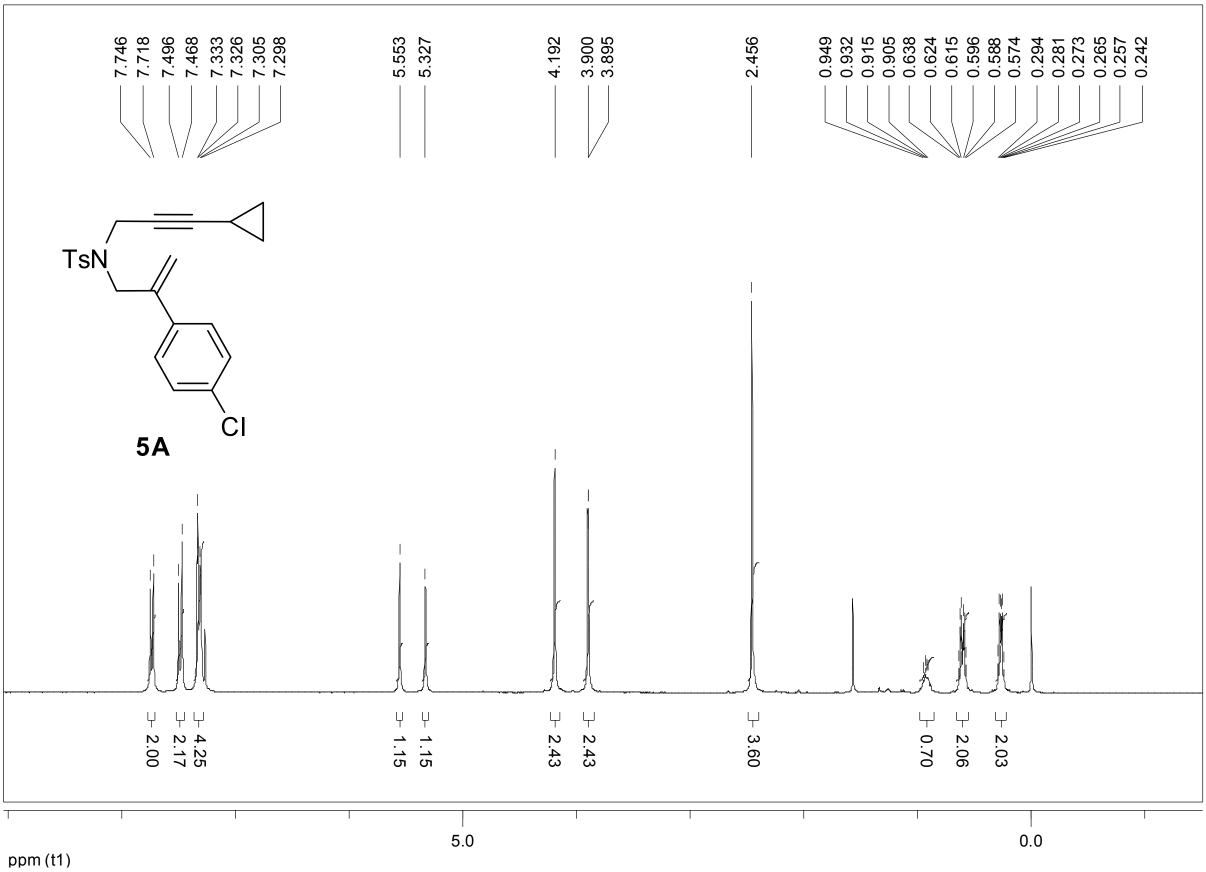
7.746
7.718
7.496
7.468
7.333
7.326
7.305
7.298
5.553
5.327
4.192
3.900
3.895
2.456
0.949
0.932
0.915
0.905
0.638
0.624
0.615
0.596
0.588
0.574
0.294
0.281
0.273
0.265
0.257
0.242
TsN
Cl
5A
2.00
2.17
4.25
1.15
1.15
2.43
2.43
3.60
0.70
2.06
2.03
5.0
0.0
ppm (t1)

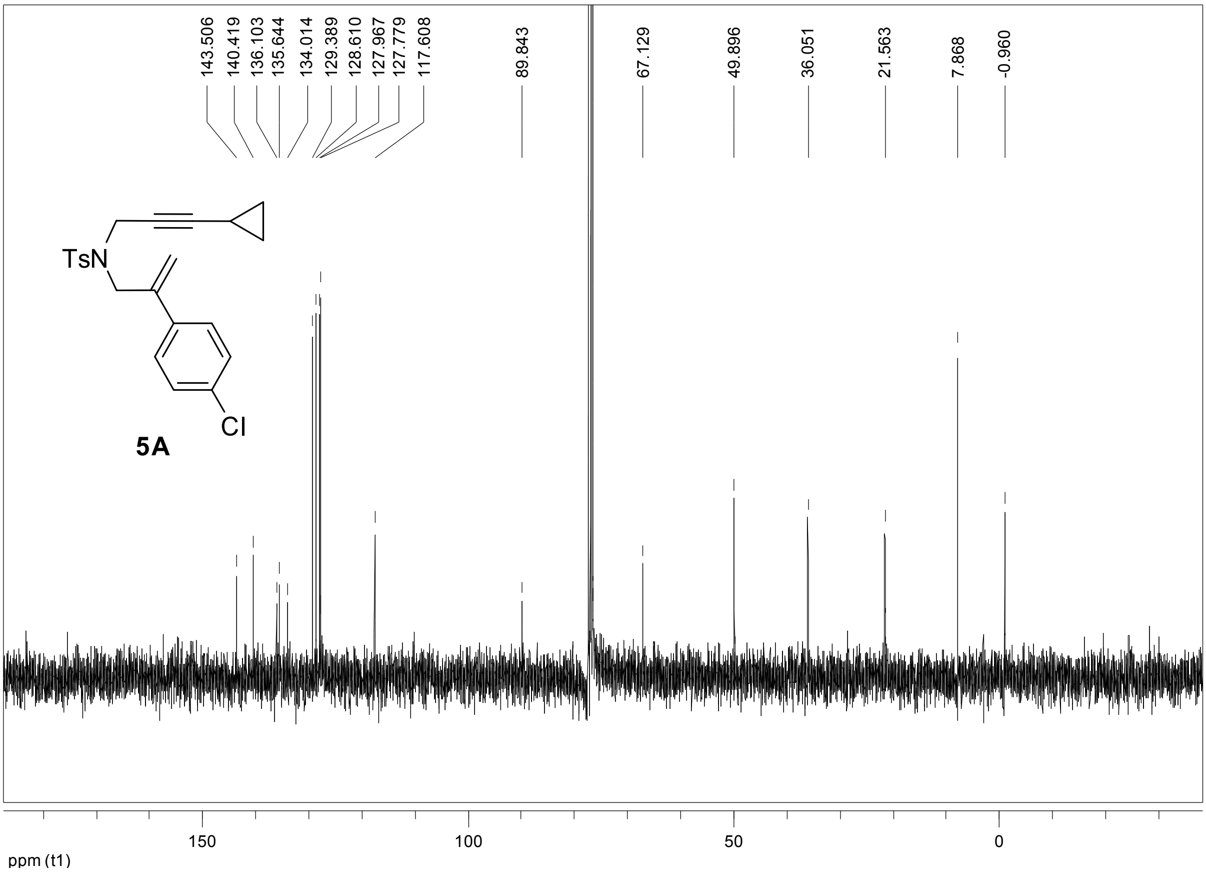
143.506
140.419
136.103
135.644
134.014
129.389
128.610
127.967
127.779
117.608
89.843
67.129
49.896
36.051
21.563
7.868
-0.960
TsN
Cl
5A
150
100
50
0
ppm (t1)

6A

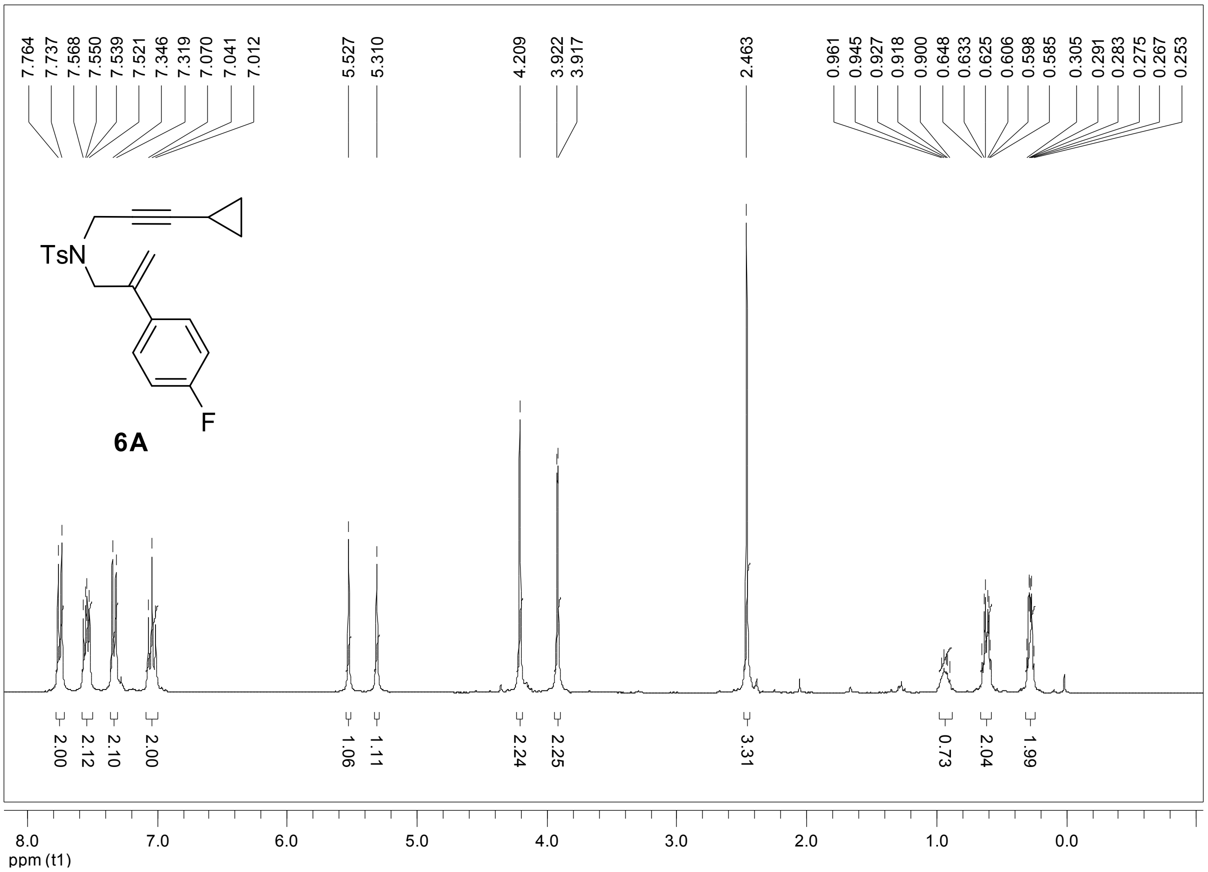
7.764
7.737
7.568
7.550
7.539
7.521
7.346
7.319
7.070
7.041
7.012
5.527
5.310
4.209
3.922
3.917
2.463
0.961
0.945
0.927
0.918
0.900
0.648
0.633
0.625
0.606
0.598
0.585
0.305
0.291
0.283
0.275
0.267
0.253
TsN
F
6A
2.00
2.12
2.10
2.00
1.06
1.11
2.24
2.25
3.31
0.73
2.04
1.99
8.0
7.0
6.0
5.0
4.0
3.0
2.0
1.0
0.0
ppm (t1)

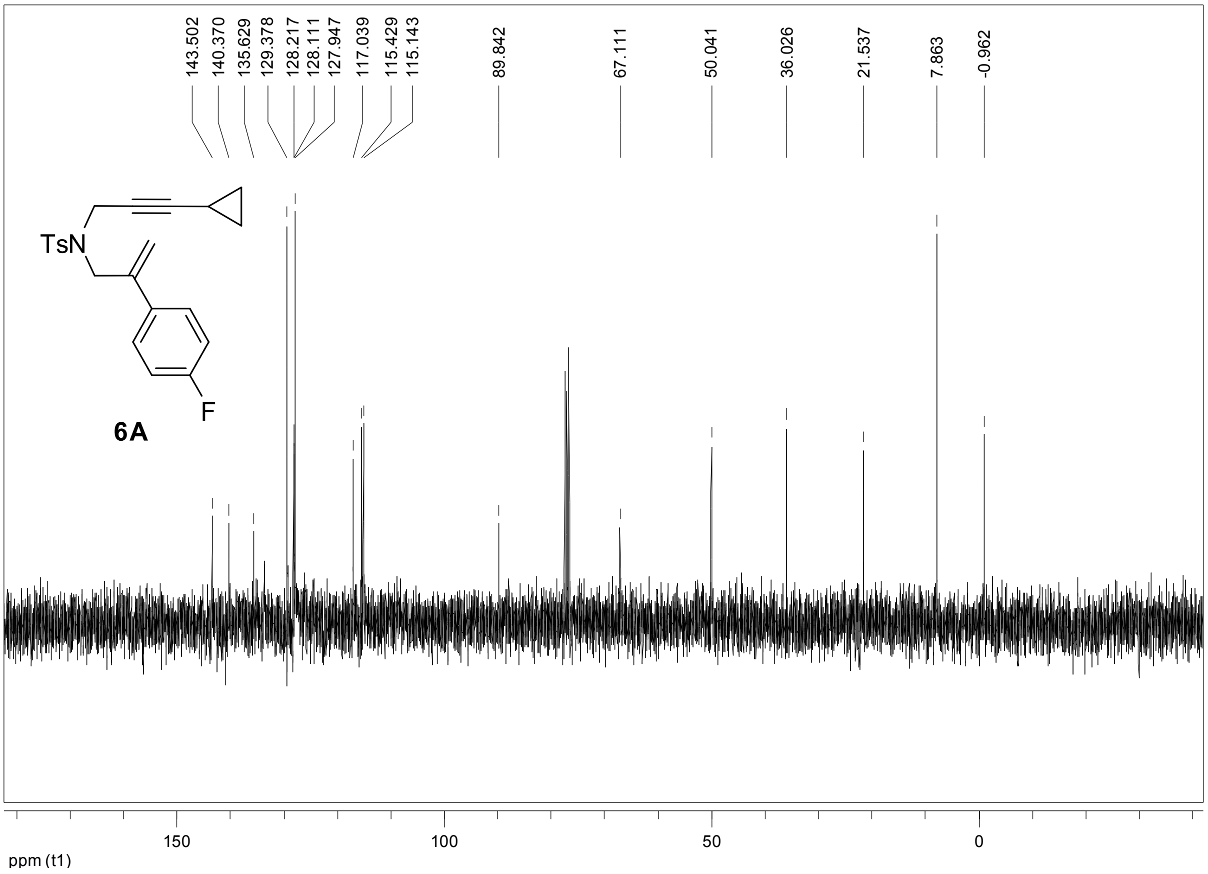
143.502
140.370
135.629
129.378
128.217
128.111
127.947
117.039
115.429
115.143
89.842
67.111
50.041
36.026
21.537
7.863
-0.962
TsN
F
6A
150
100
50
0
ppm (t1)

7A

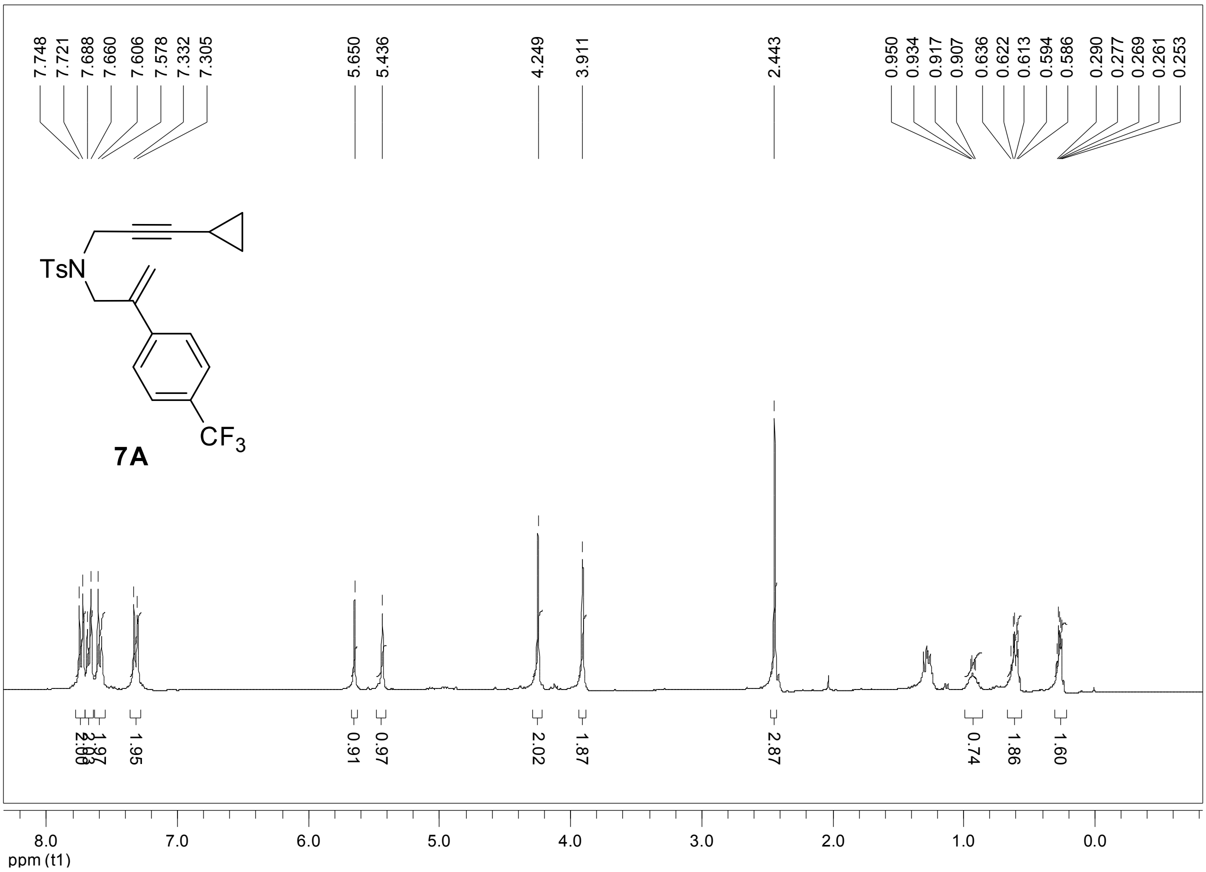
7.748
7.721
7.688
7.660
7.606
7.578
7.332
7.305
5.650
5.436
4.249
3.911
2.443
0.950
0.934
0.917
0.907
0.636
0.622
0.613
0.594
0.586
0.290
0.277
0.269
0.261
0.253
TsN
CF3
7A
1.95
0.91
0.97
2.02
1.87
2.87
0.74
1.86
1.60
8.0
7.0
6.0
5.0
4.0
3.0
2.0
1.0
0.0
ppm (t1)

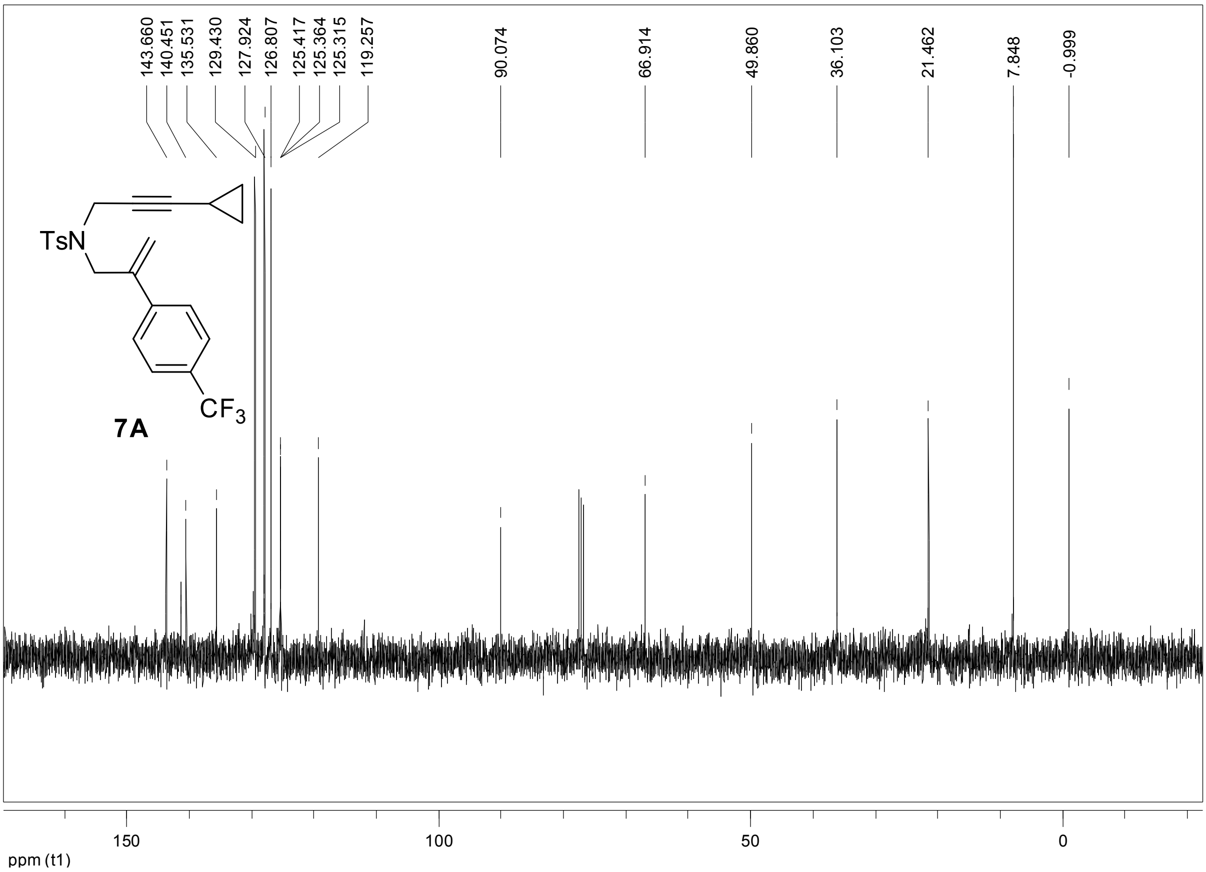
143.660
140.451
135.531
129.430
127.924
126.807
125.417
125.364
125.315
119.257
90.074
66.914
49.860
36.103
21.462
7.848
-0.999
TsN
CF3
7A
150
100
50
0
ppm (t1)

8A

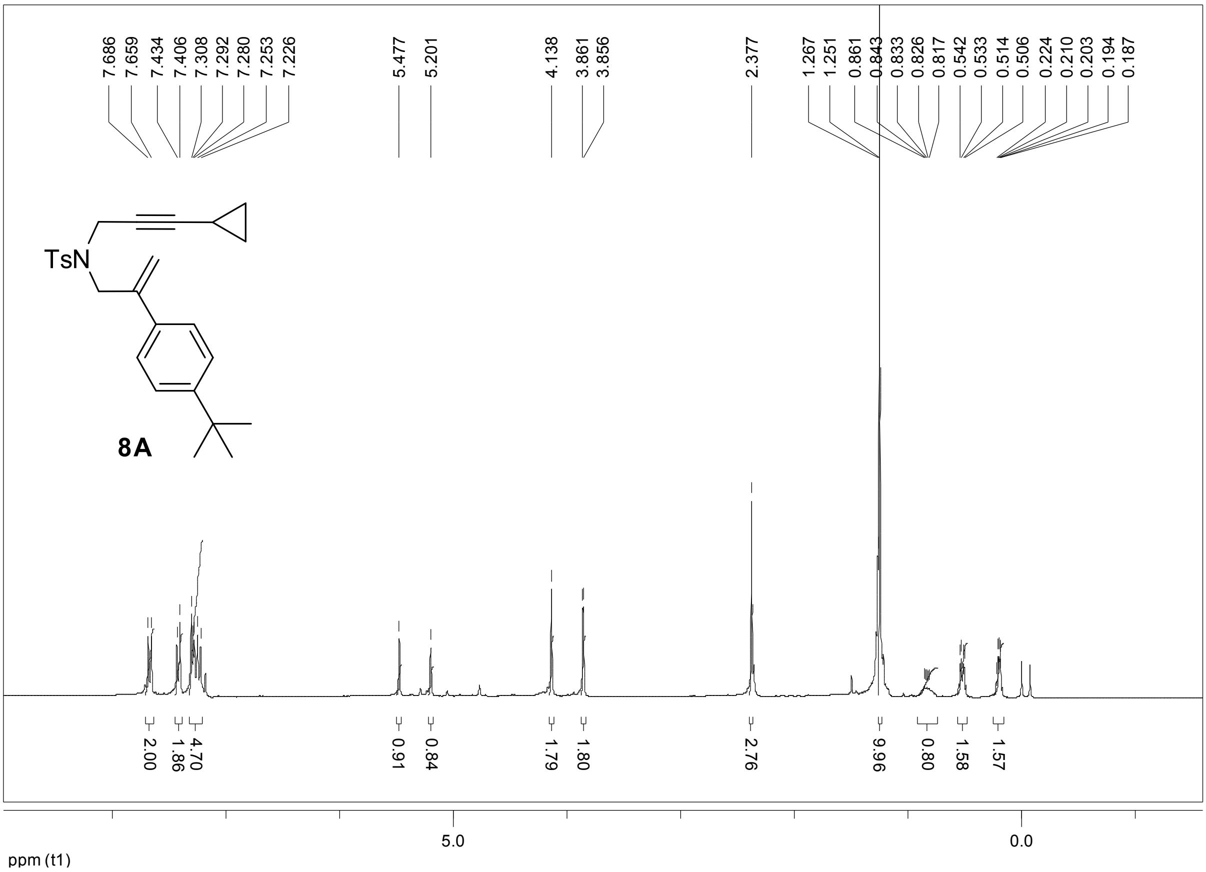
7.686
7.659
7.434
7.406
7.308
7.292
7.280
7.253
7.226
5.477
5.201
4.138
3.861
3.856
2.377
1.267
1.251
0.861
0.843
0.833
0.826
0.817
0.542
0.533
0.514
0.506
0.224
0.210
0.203
0.194
0.187
TsN
8A
2.00
1.86
4.70
0.91
0.84
1.79
1.80
2.76
9.96
0.80
1.58
1.57
5.0
0.0
ppm (t1)

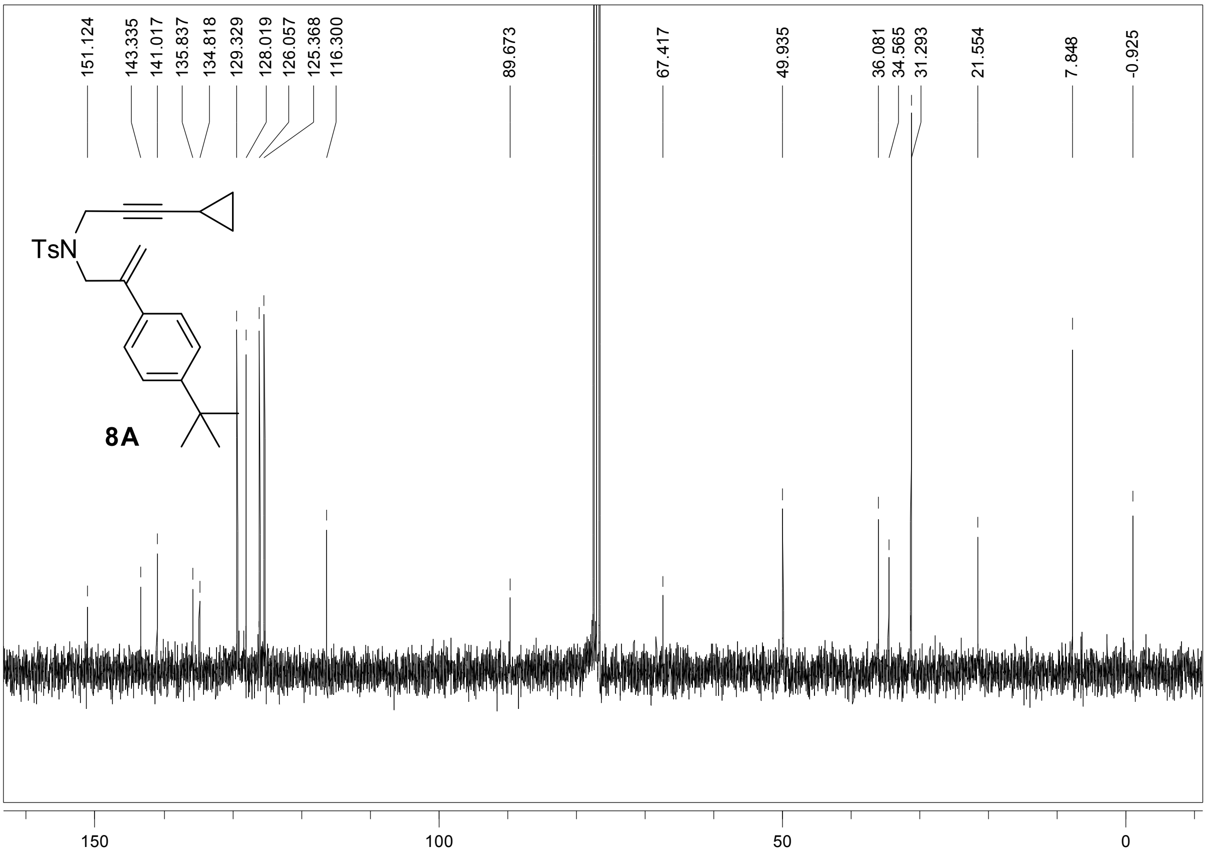
151.124
143.335
141.017
135.837
134.818
129.329
128.019
126.057
125.368
116.300
89.673
67.417
49.935
36.081
34.565
31.293
21.554
7.848
-0.925
TsN
8A
150
100
50
0
ppm (t1)

9A

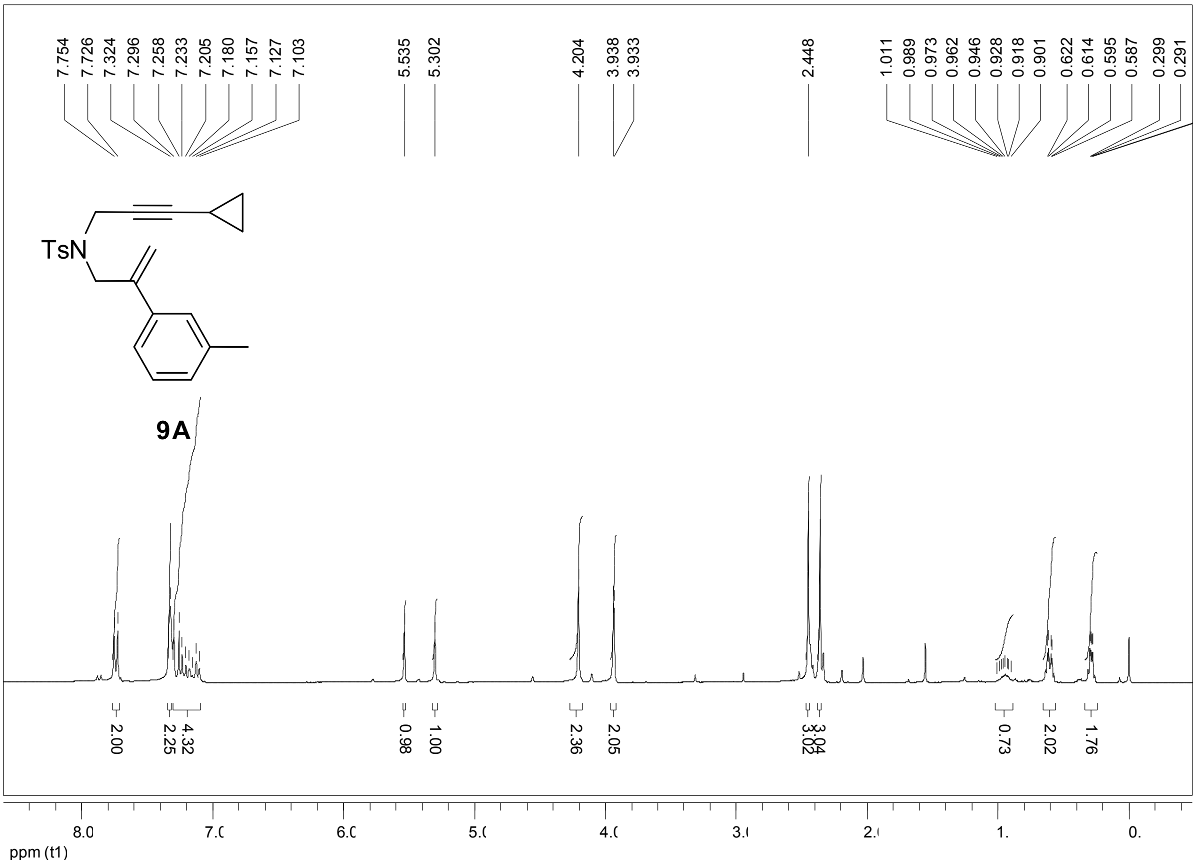

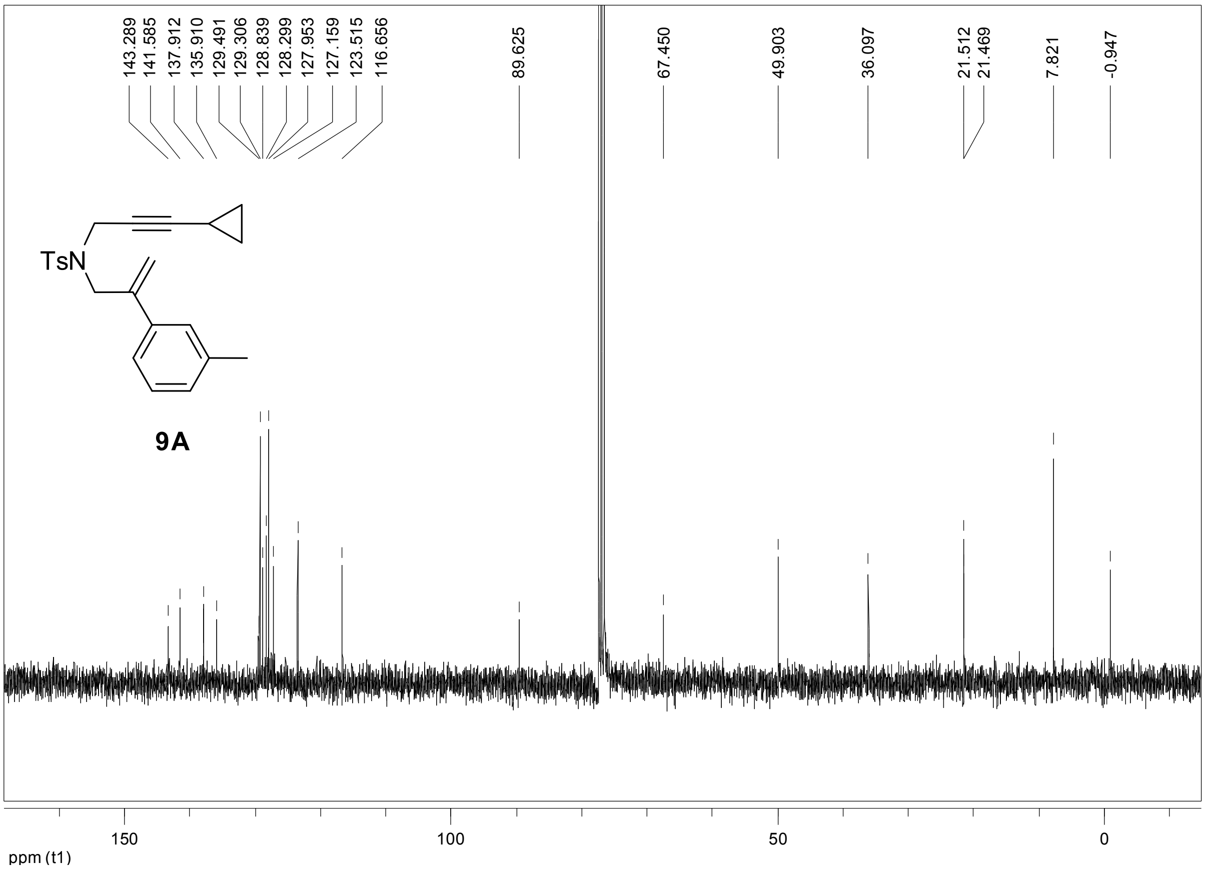

10A

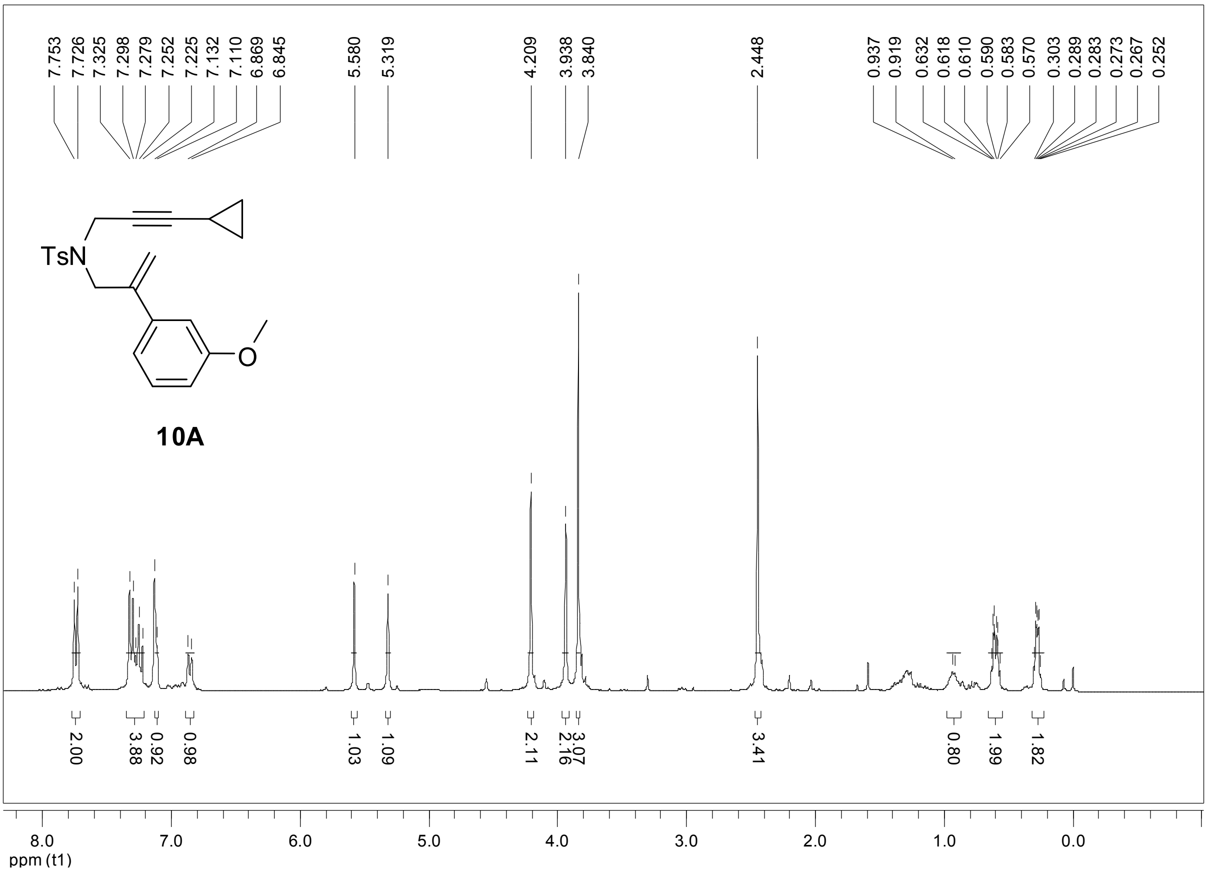

7.753
7.726
7.325
7.298
7.279
7.252
7.225
7.132
7.110
6.869
6.845
5.580
5.319
4.209
3.938
3.840
2.448
0.937
0.919
0.632
0.618
0.610
0.590
0.583
0.570
0.303
0.289
0.283
0.273
0.267
0.252
TsN
O
10A
2.00
3.88
0.92
0.98
1.03
1.09
2.11
2.16
3.07
3.41
0.80
1.99
1.82
8.0
7.0
6.0
5.0
4.0
3.0
2.0
1.0
0.0
ppm (t1)

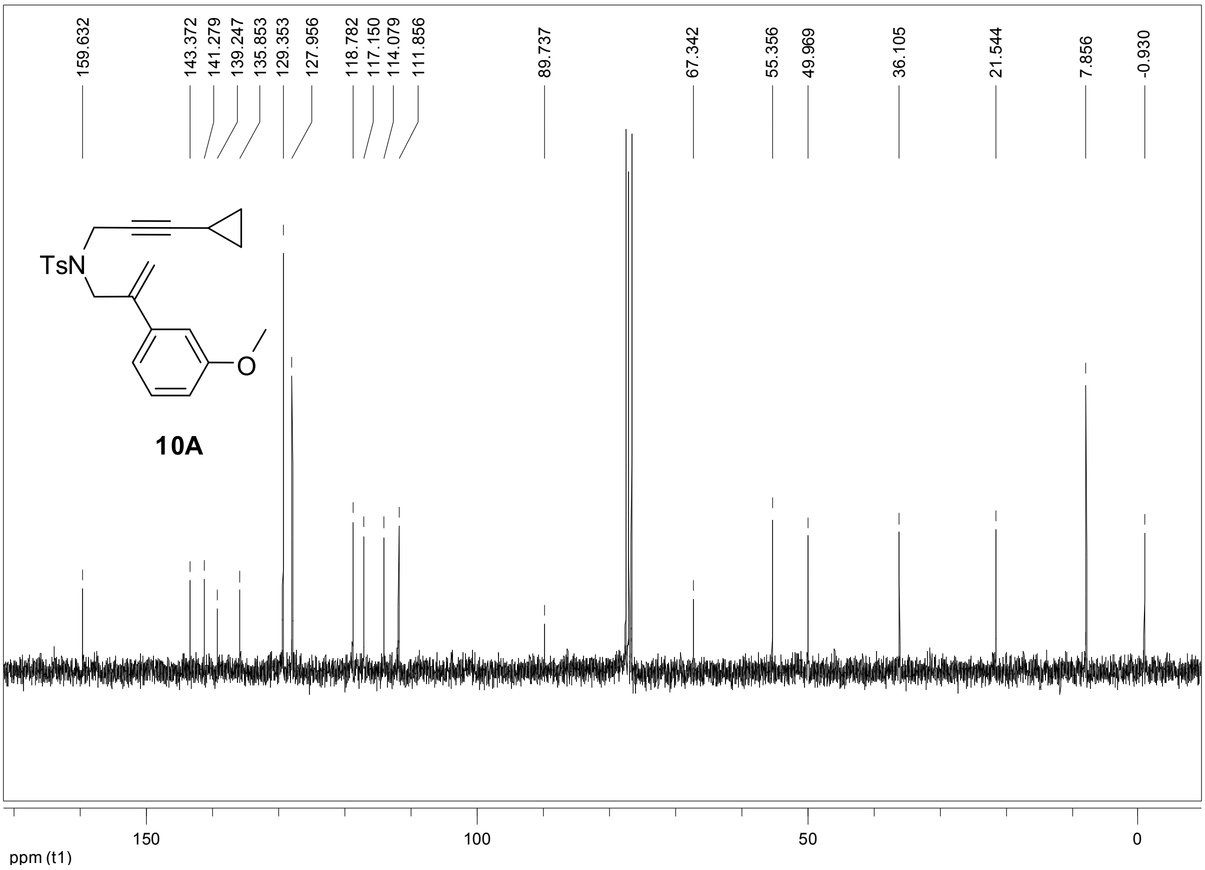

159.632
143.372
141.279
139.247
135.853
129.353
127.956
118.782
117.150
114.079
111.856
89.737
67.342
55.356
49.969
36.105
21.544
7.856
-0.930
TsN
O
10A
150
100
50
0
ppm (t1)

11A

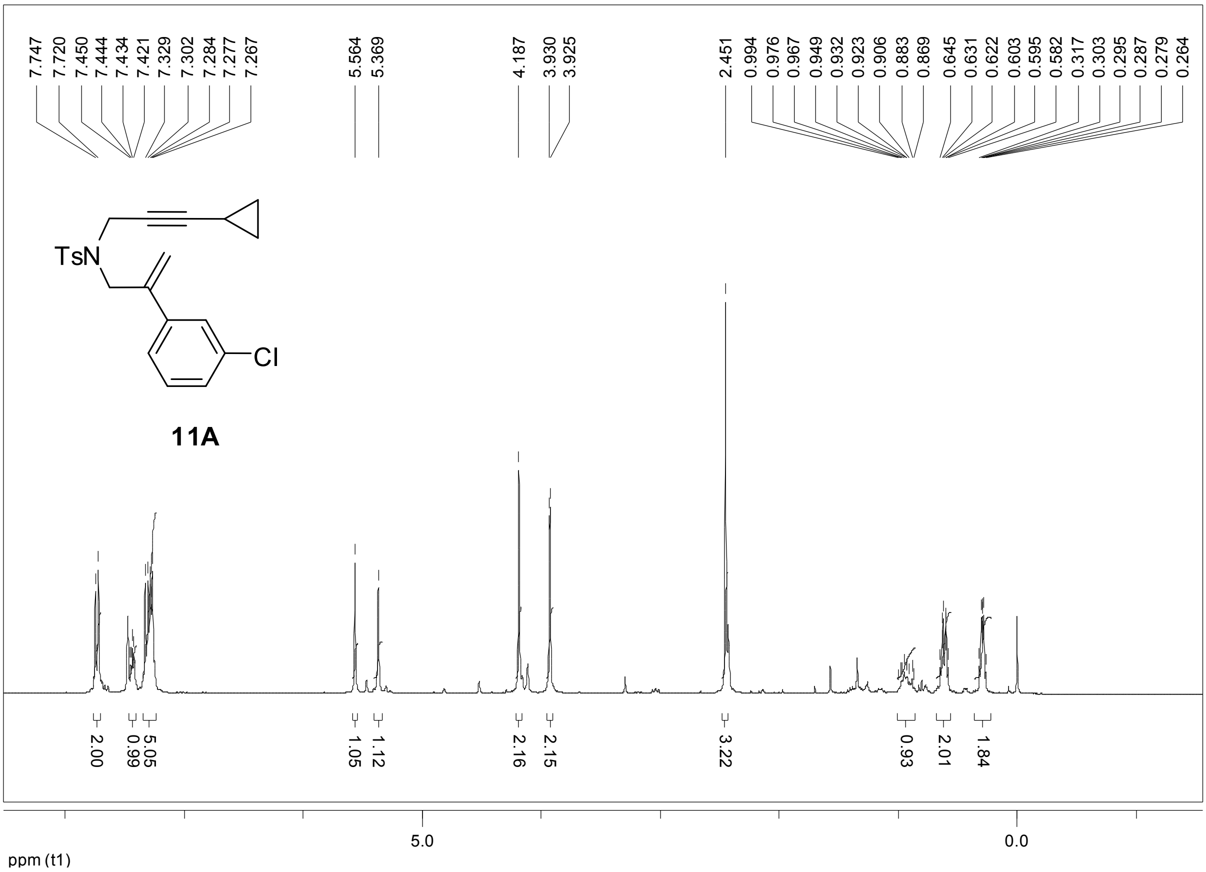
7.747
7.720
7.450
7.444
7.434
7.421
7.329
7.302
7.284
7.277
7.267
5.564
5.369
4.187
3.930
3.925
2.451
0.994
0.976
0.967
0.949
0.932
0.923
0.906
0.883
0.869
0.645
0.631
0.622
0.603
0.595
0.582
0.317
0.303
0.295
0.287
0.279
0.264
TsN
Cl
11A
2.00
0.99
5.05
1.05
1.12
2.16
2.15
3.22
0.93
2.01
1.84
5.0
0.0
ppm (t1)

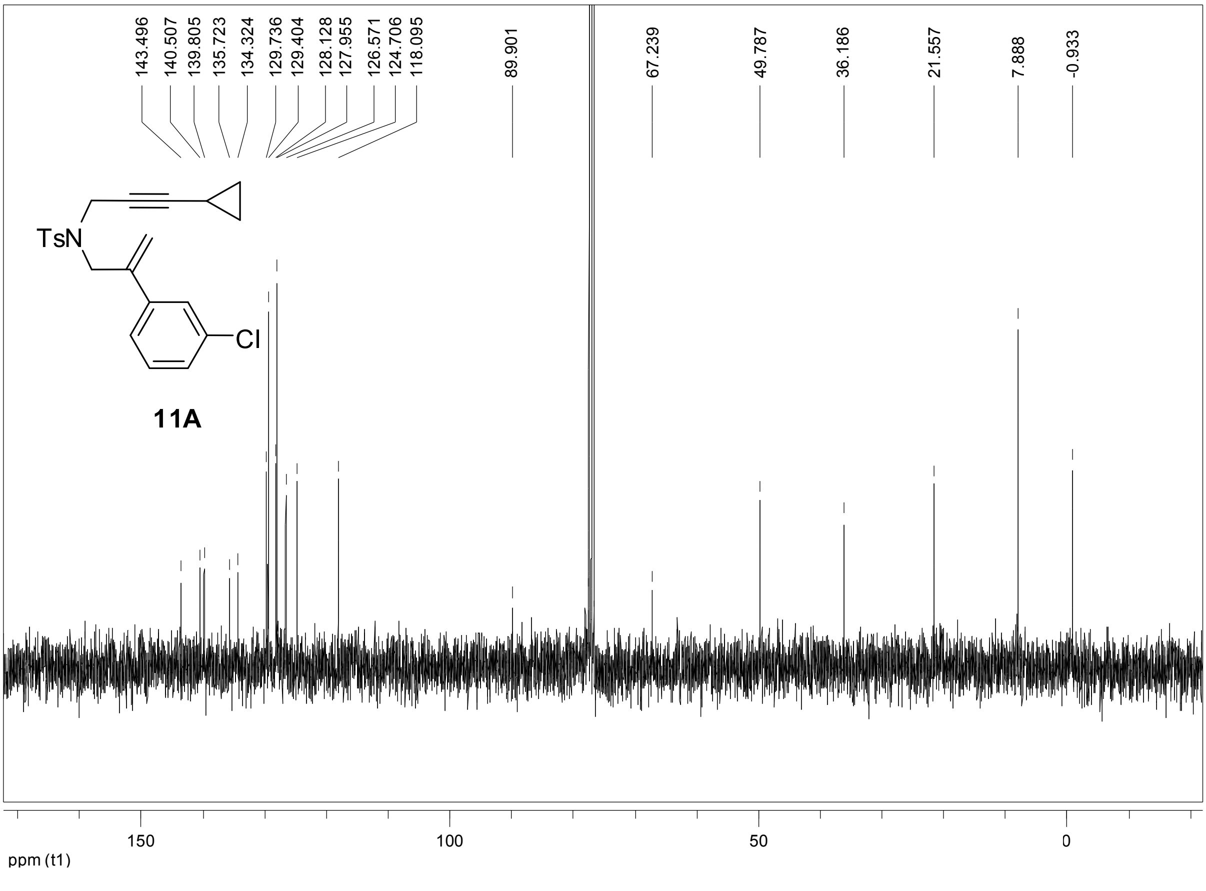
143.496
140.507
139.805
135.723
134.324
129.736
129.404
128.128
127.955
126.571
124.706
118.095
89.901
67.239
49.787
36.186
21.557
7.888
-0.933
TsN
Cl
11A
150
100
50
0
ppm (t1)

12A

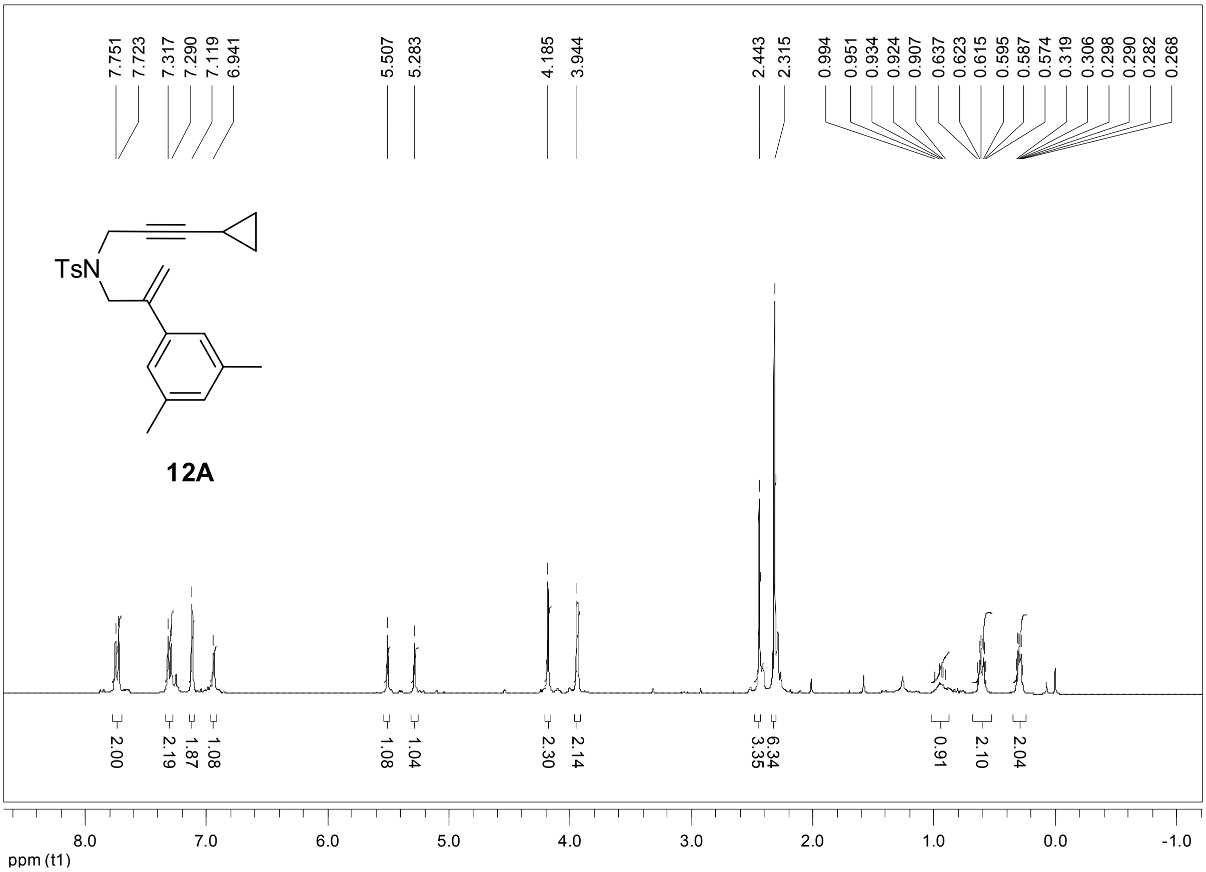

7.751
7.723
7.317
7.290
7.119
6.941
5.507
5.283
4.185
3.944
2.443
2.315
0.994
0.951
0.934
0.924
0.907
0.637
0.623
0.615
0.595
0.587
0.574
0.319
0.306
0.298
0.290
0.282
0.268
TsN
12A
2.00
2.19
1.87
1.08
1.08
1.04
2.30
2.14
3.35
6.34
0.91
2.10
2.04
8.0
7.0
6.0
5.0
4.0
3.0
2.0
1.0
0.0
-1.0
ppm (t1)

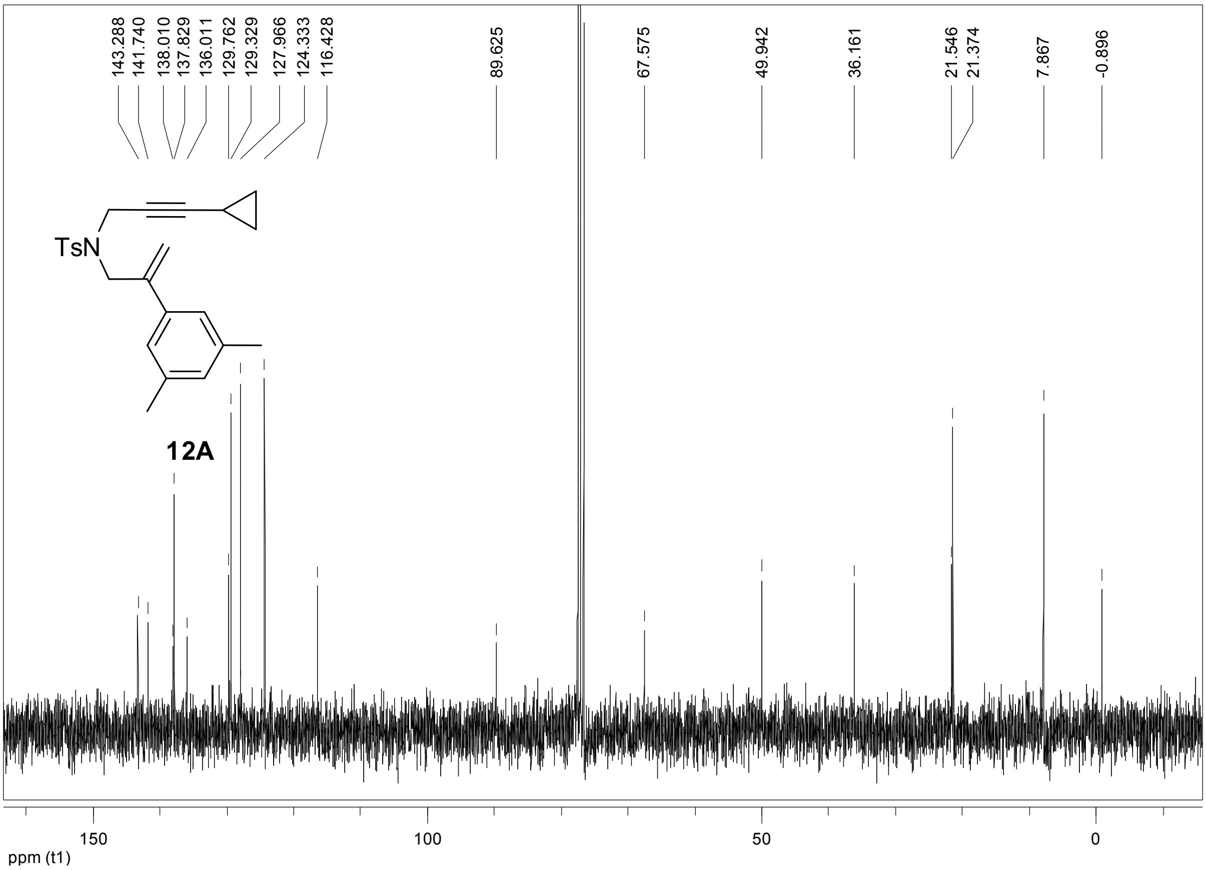

143.288
141.740
138.010
137.829
136.011
129.762
129.329
127.966
124.333
116.428
89.625
67.575
49.942
36.161
21.546
21.374
7.867
-0.896
TsN
12A
150
100
50
0
ppm (t1)

13A

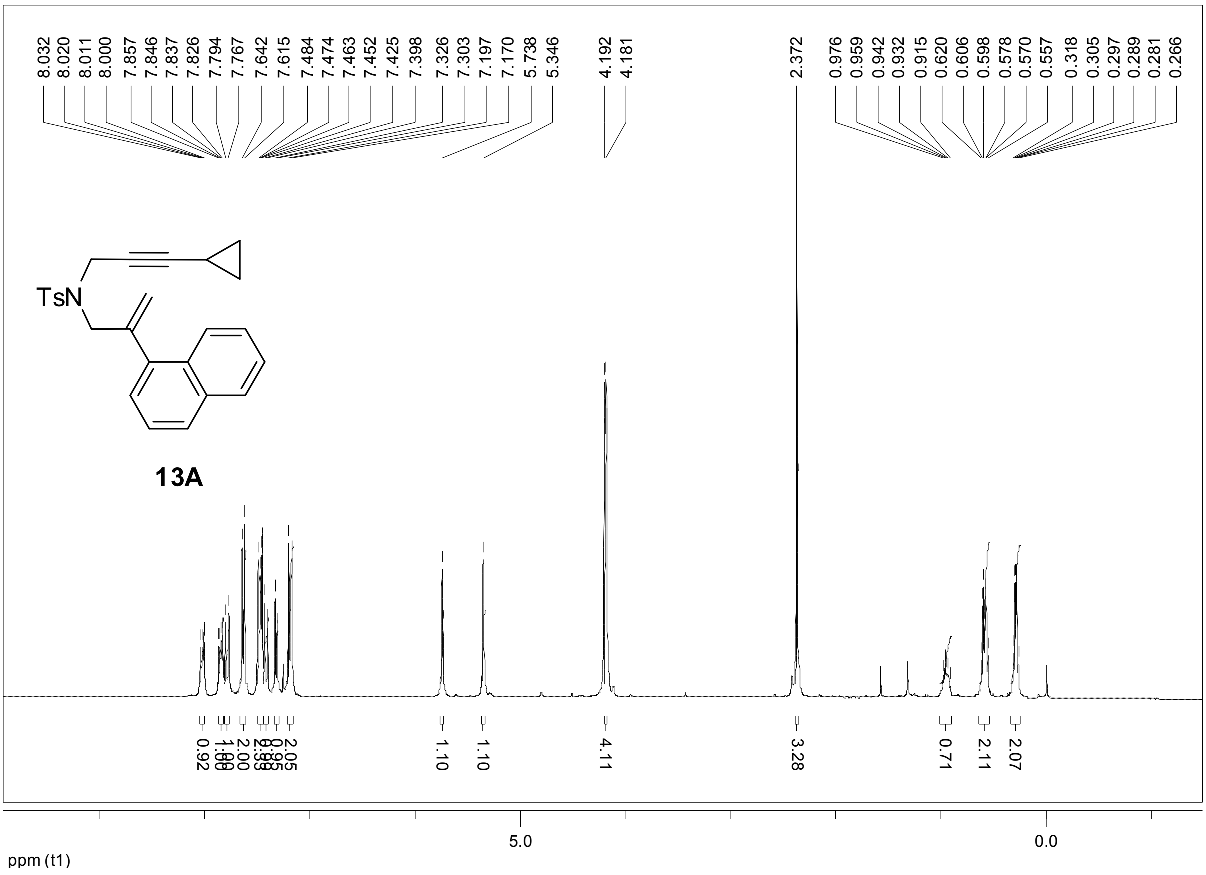
8.032 8.020 8.011 8.000 7.857 7.846 7.837 7.826 7.794 7.767 7.642 7.615 7.484 7.474 7.463 7.452 7.425 7.398 7.326 7.303 7.197 7.170 5.738 5.346
4.192 4.181
2.372
0.976 0.959 0.942 0.932 0.915 0.620 0.606 0.598 0.578 0.570 0.557 0.318 0.305 0.297 0.289 0.281 0.266
TsN
13A
0.92 2.00 2.05 1.10 1.10 4.11 3.28 0.71 2.11 2.07
5.0
0.0
ppm (t1)

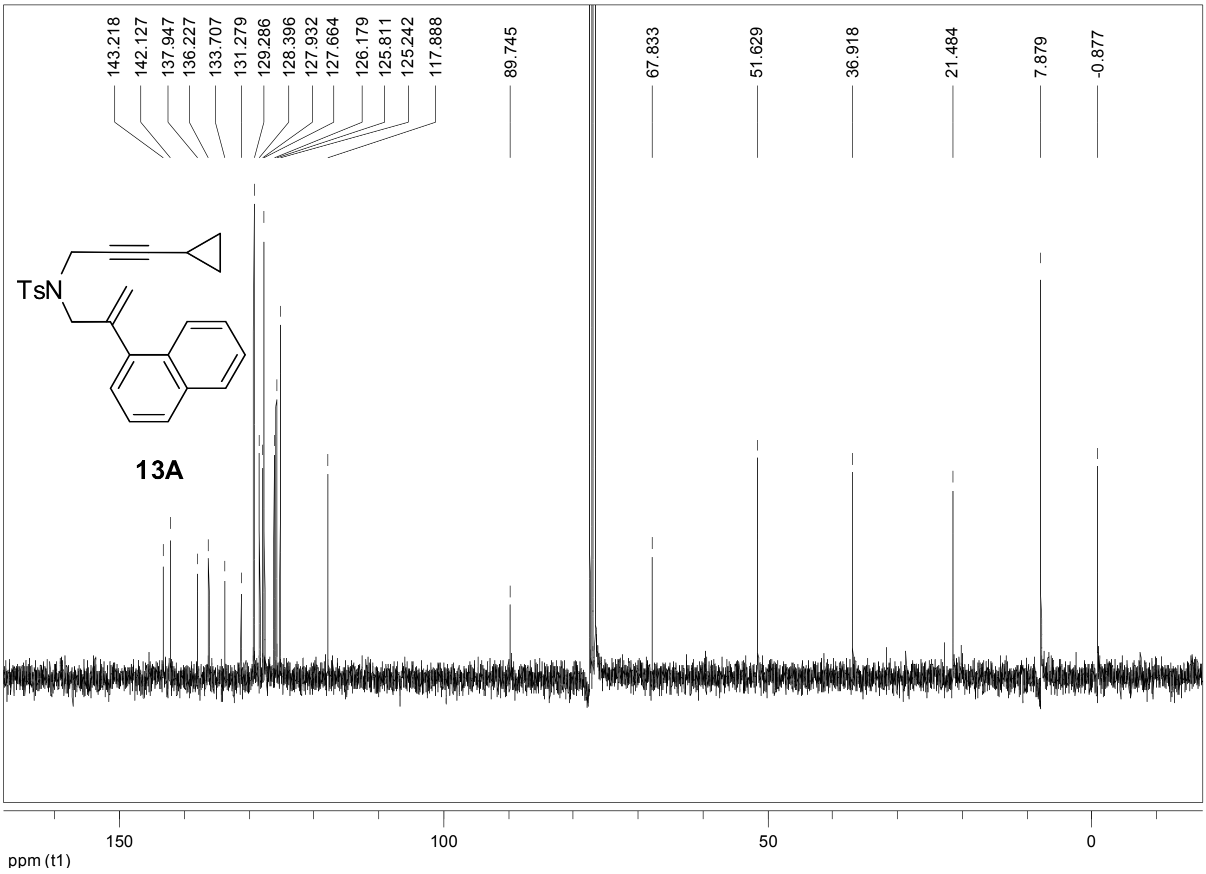
143.218 142.127 137.947 136.227 133.707 131.279 129.286 128.396 127.932 127.664 126.179 125.811 125.242 117.888
89.745
67.833
51.629
36.918
21.484
7.879
-0.877
TsN
13A
150
100
50
0
ppm (t1)

14A

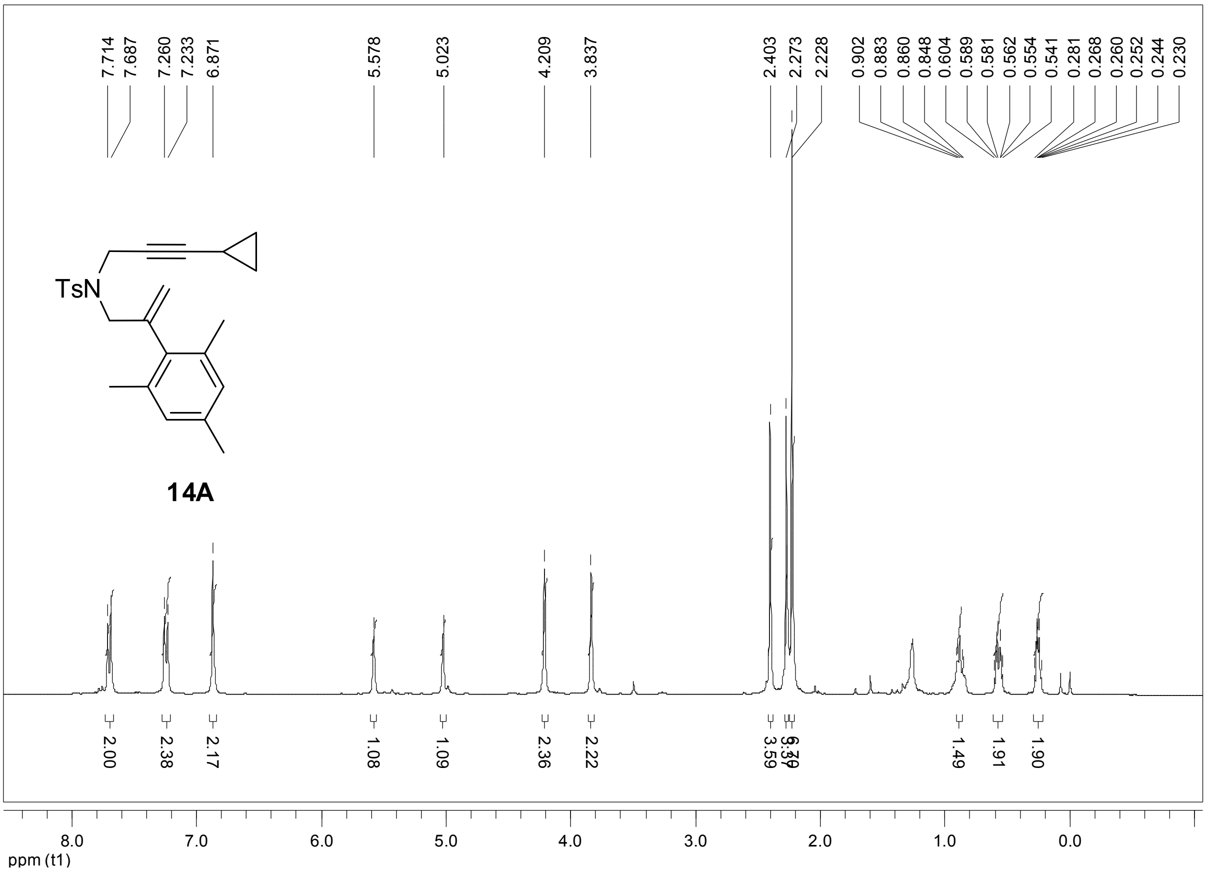
7.714
7.687
7.260
7.233
6.871
5.578
5.023
4.209
3.837
2.403
2.273
2.228
0.902
0.883
0.860
0.848
0.604
0.589
0.581
0.562
0.554
0.541
0.281
0.268
0.260
0.252
0.244
0.230
TsN
14A
2.00
2.38
2.17
1.08
1.09
2.36
2.22
3.59
1.49
1.91
1.90
8.0
7.0
6.0
5.0
4.0
3.0
2.0
1.0
0.0
ppm (t1)

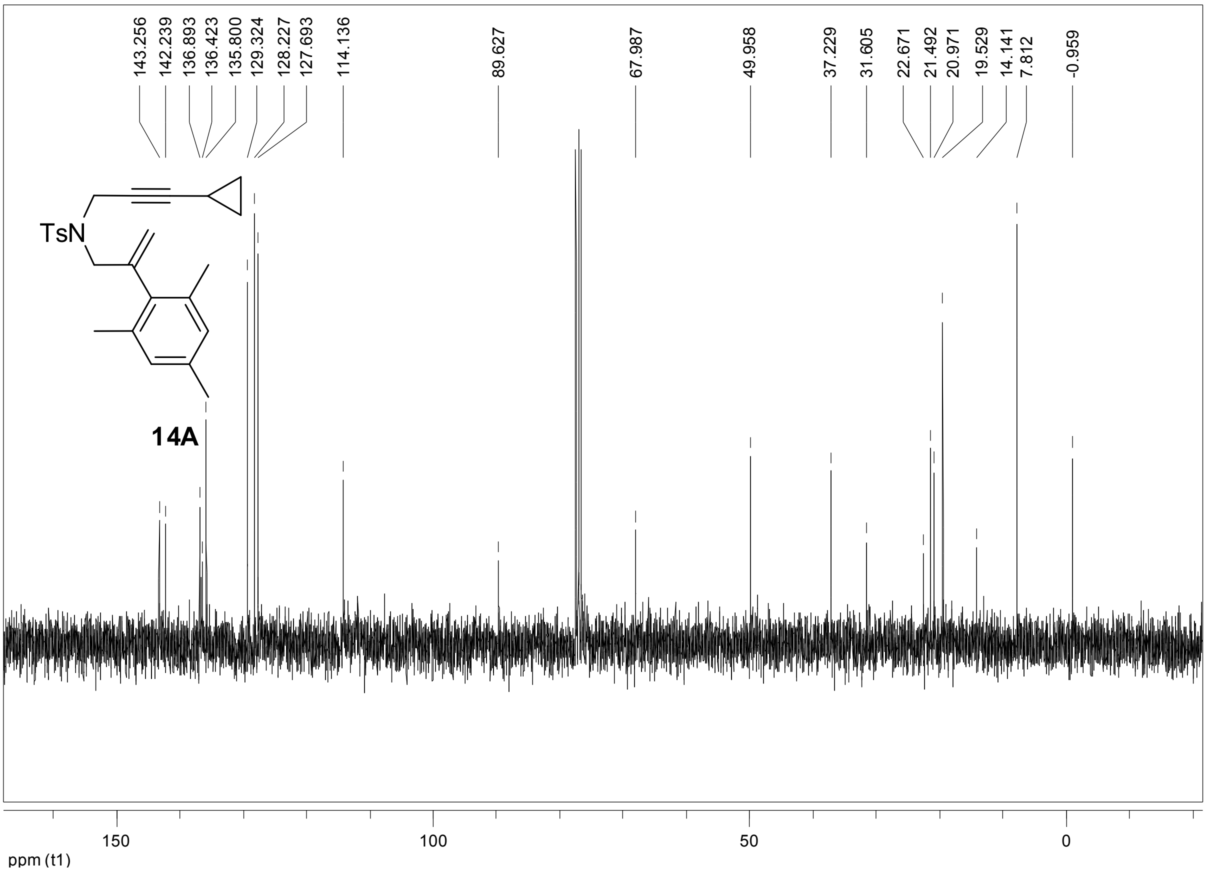
143.256
142.239
136.893
136.423
135.800
129.324
128.227
127.693
114.136
89.627
67.987
49.958
37.229
31.605
22.671
21.492
20.971
19.529
14.141
7.812
-0.959
TsN
14A
150
100
50
0
ppm (t1)

15A

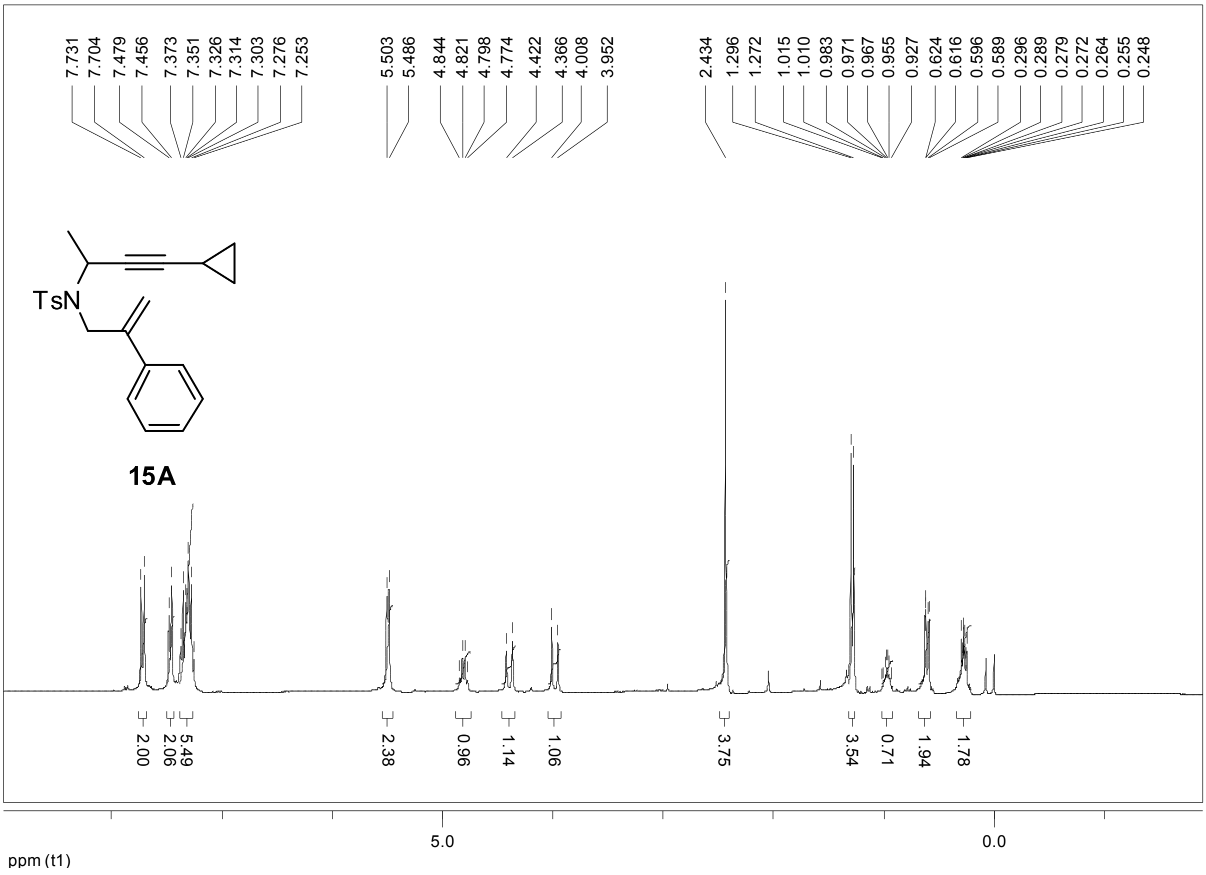

7.731
7.704
7.479
7.456
7.373
7.351
7.326
7.314
7.303
7.276
7.253
5.503
5.486
4.844
4.821
4.798
4.774
4.422
4.366
4.008
3.952
2.434
1.296
1.272
1.015
1.010
0.983
0.971
0.967
0.955
0.927
0.624
0.616
0.596
0.589
0.296
0.289
0.279
0.272
0.264
0.255
0.248
TsN
15A
2.00
2.06
5.49
2.38
0.96
1.14
1.06
3.75
3.54
0.71
1.94
1.78
5.0
0.0
ppm (t1)

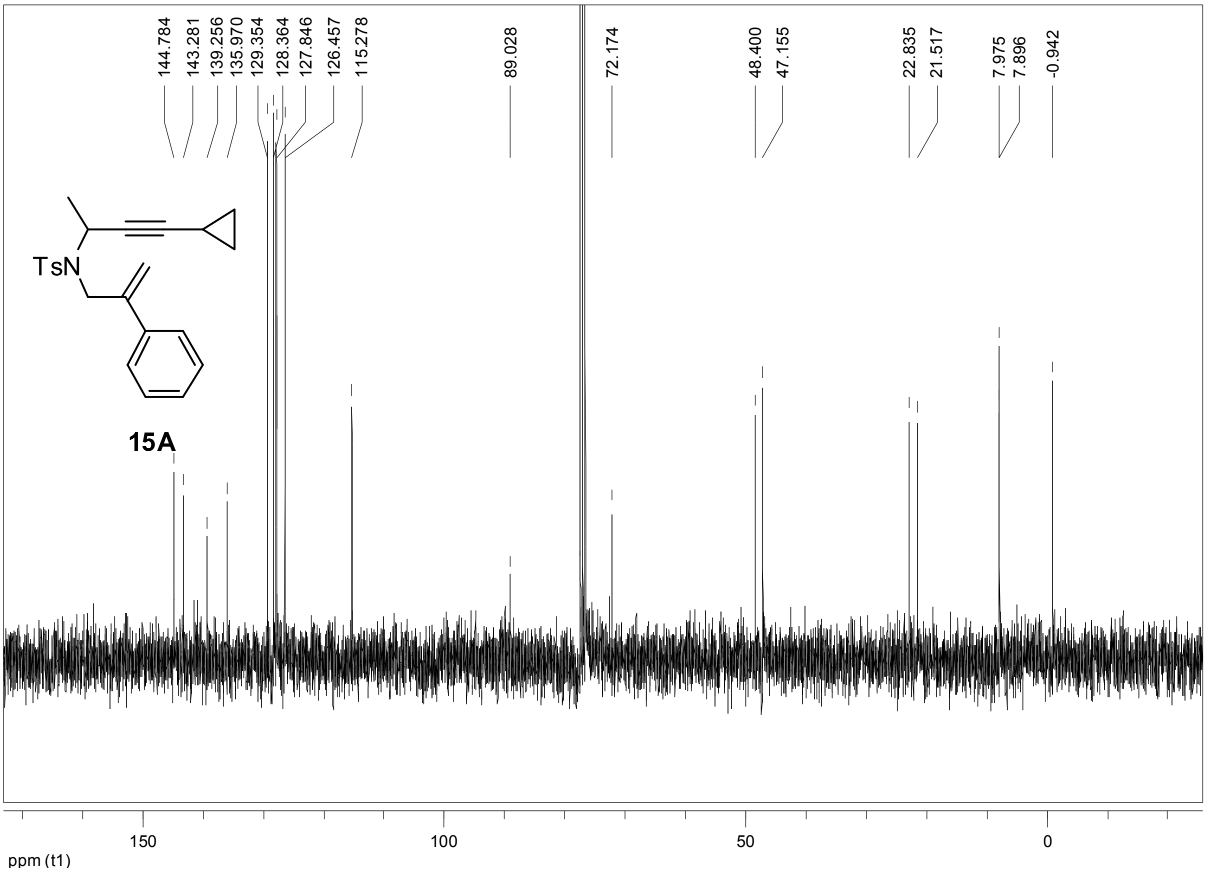

144.784
143.281
139.256
135.970
129.354
128.364
127.846
126.457
115.278
89.028
72.174
48.400
47.155
22.835
21.517
7.975
7.896
-0.942
TsN
15A
150
100
50
0
ppm (t1)

16A

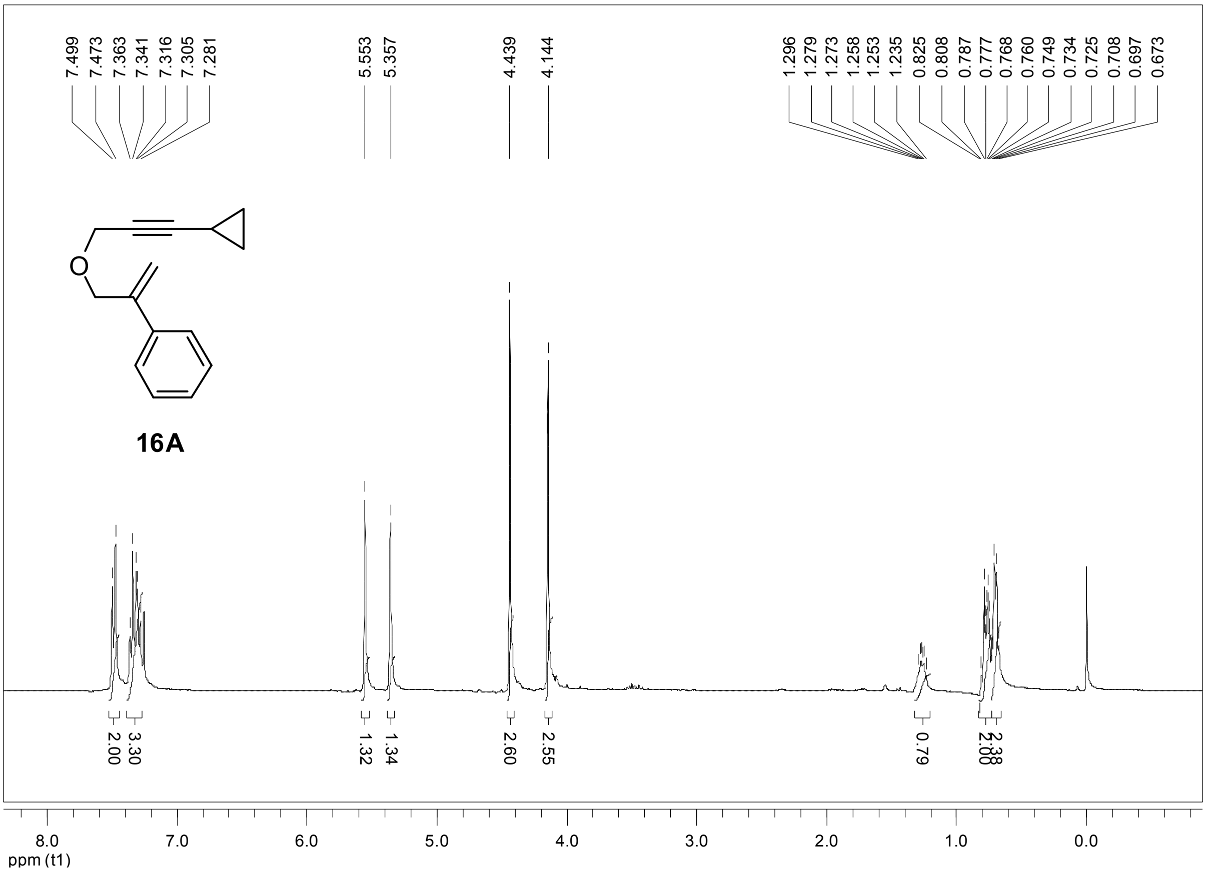
7.499
7.473
7.363
7.341
7.316
7.305
7.281
5.553
5.357
4.439
4.144
1.296
1.279
1.273
1.258
1.253
1.235
0.825
0.808
0.787
0.777
0.768
0.760
0.749
0.734
0.725
0.708
0.697
0.673
O
16A
2.00
3.30
1.32
1.34
2.60
2.55
0.79
2.00
2.38
8.0
7.0
6.0
5.0
4.0
3.0
2.0
1.0
0.0
ppm (t1)

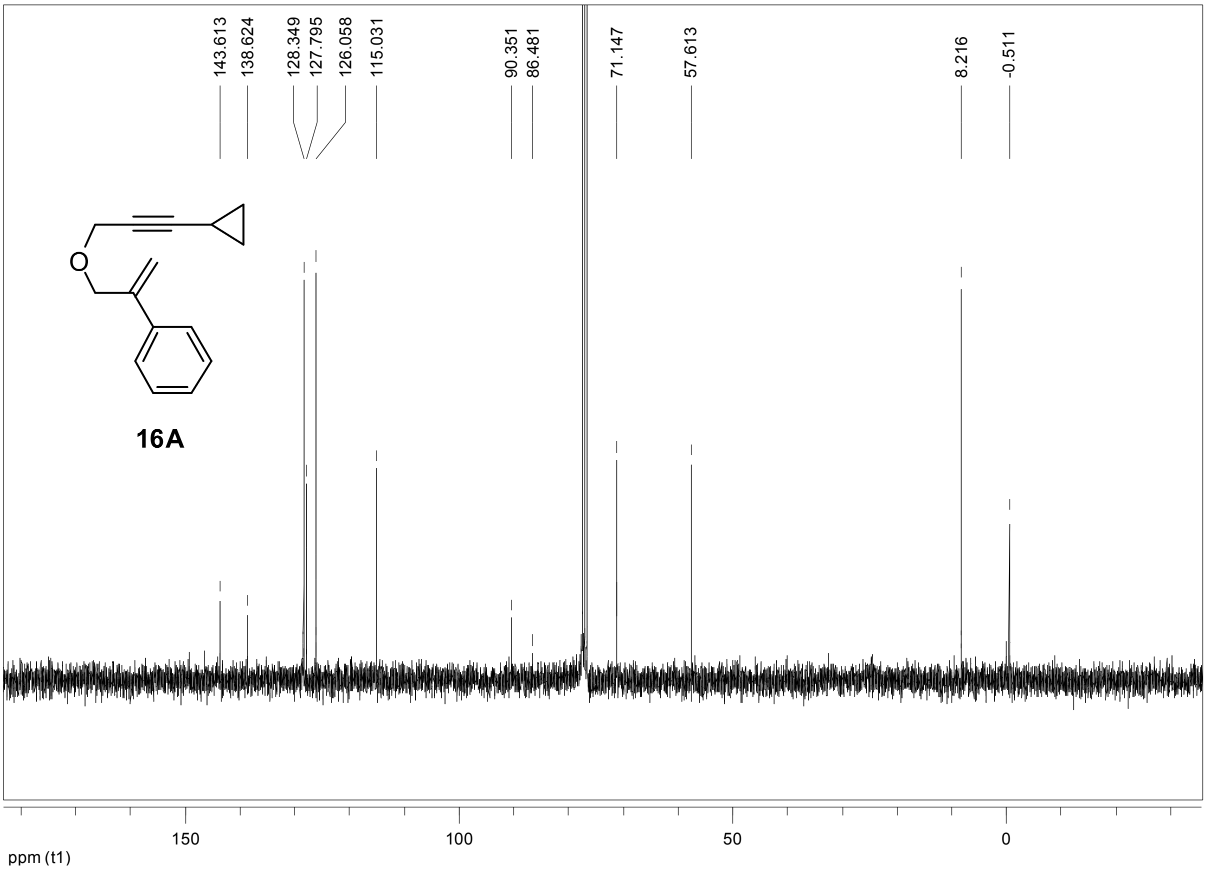
143.613
138.624
128.349
127.795
126.058
115.031
90.351
86.481
71.147
57.613
8.216
-0.511
O
16A
150
100
50
0
ppm (t1)

1B

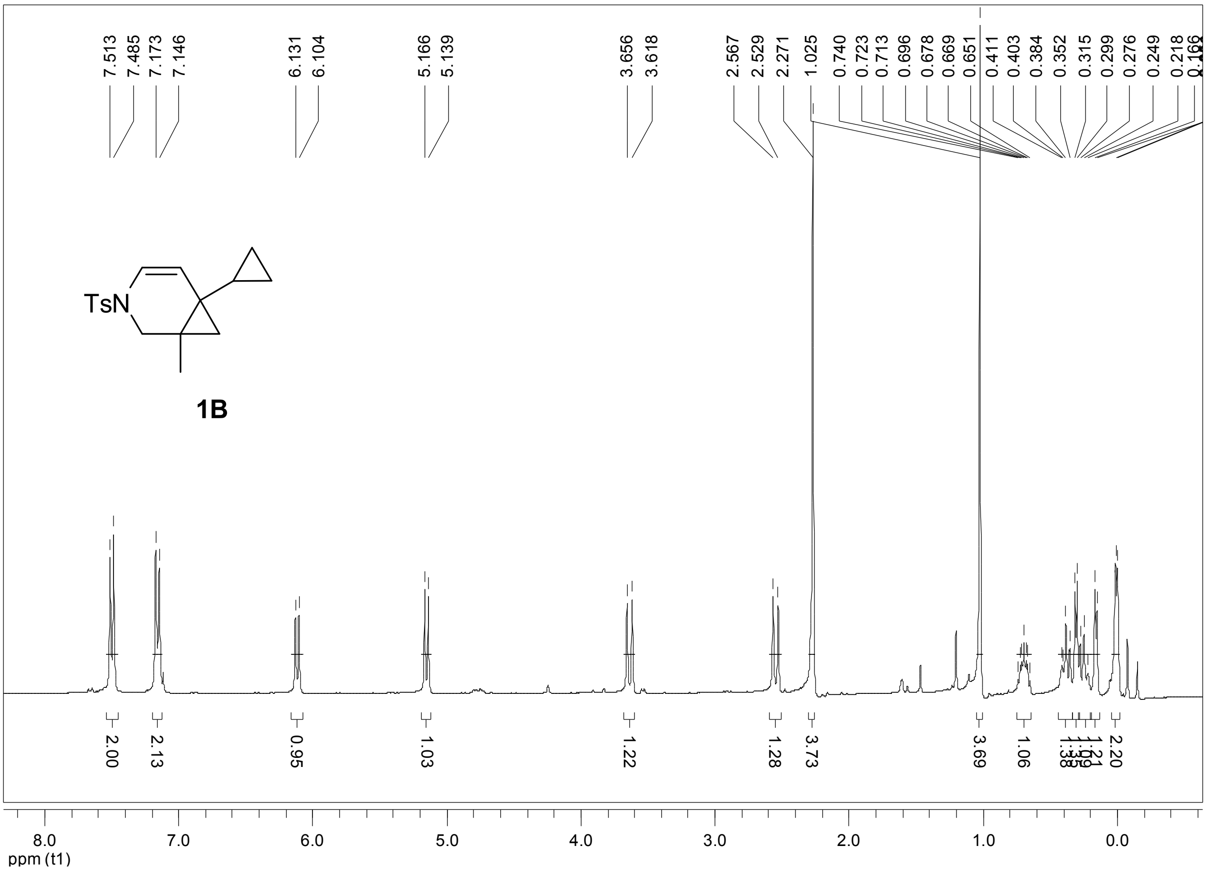
7.513
7.485
7.173
7.146
6.131
6.104
5.166
5.139
3.656
3.618
2.567
2.529
2.271
1.025
0.740
0.723
0.713
0.696
0.678
0.669
0.651
0.411
0.403
0.384
0.352
0.315
0.299
0.276
0.249
0.218
TsN
1B
2.00
2.13
0.95
1.03
1.22
1.28
3.73
3.69
1.06
2.20
8.0
7.0
6.0
5.0
4.0
3.0
2.0
1.0
0.0
ppm (t1)

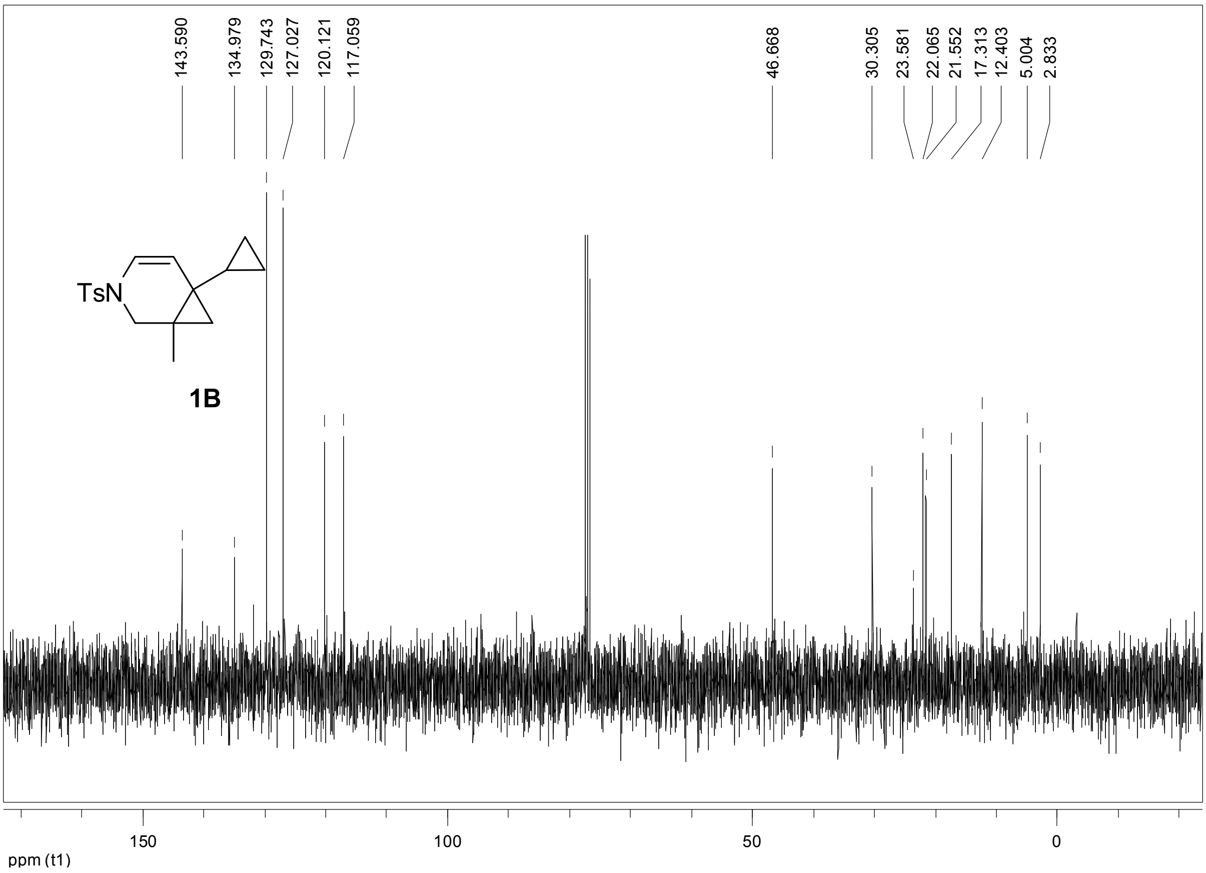
143.590
134.979
129.743
127.027
120.121
117.059
46.668
30.305
23.581
22.065
21.552
17.313
12.403
5.004
2.833
TsN
1B
150
100
50
0
ppm (t1)

2B

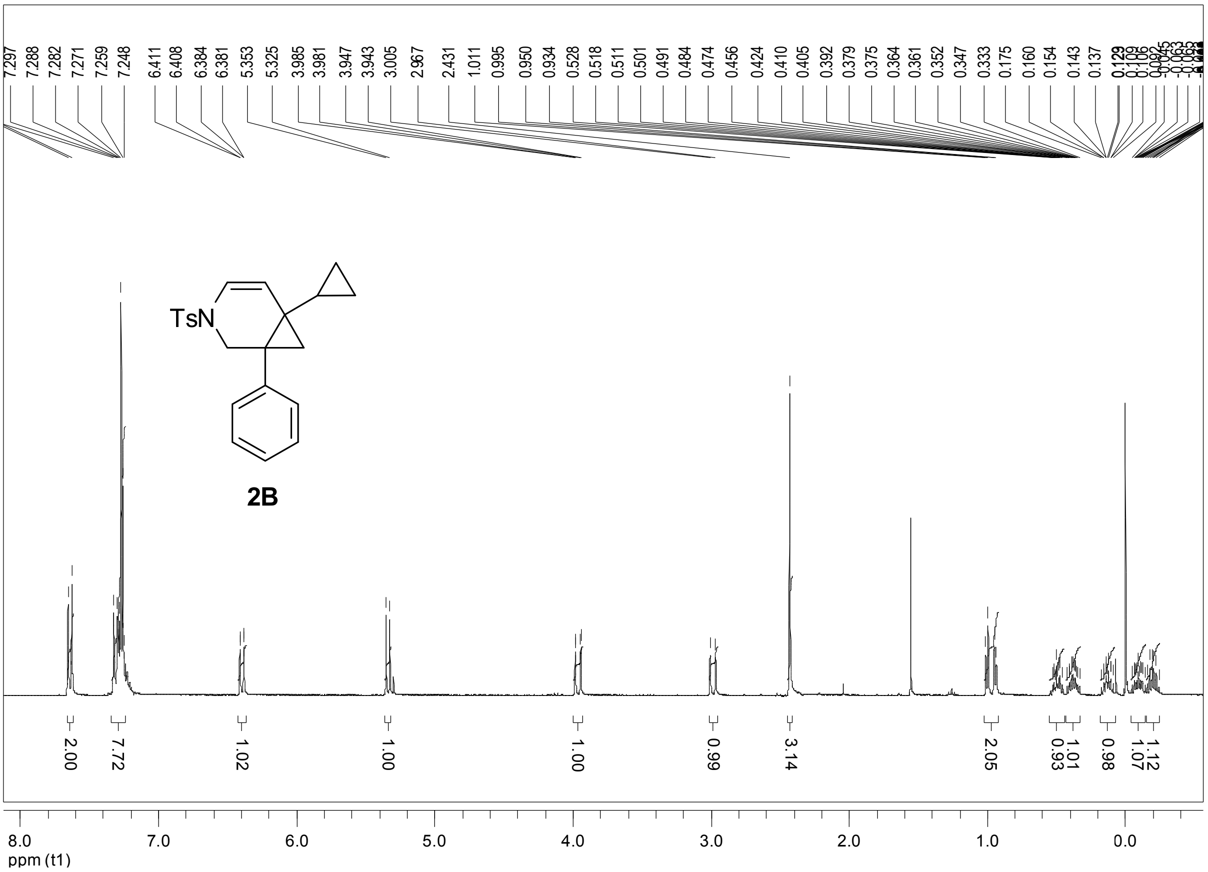
7.297
7.288
7.282
7.271
7.259
7.248
6.411
6.408
6.384
6.381
5.353
5.325
3.985
3.981
3.947
3.943
3.005
2.967
2.431
1.011
0.995
0.950
0.934
0.528
0.518
0.511
0.501
0.491
0.484
0.474
0.456
0.424
0.410
0.405
0.392
0.379
0.375
0.364
0.361
0.352
0.347
0.333
0.175
0.160
0.154
0.143
0.137
0.106
TsN
2B
2.00
7.72
1.02
1.00
1.00
0.99
3.14
2.05
0.93
1.01
0.98
1.07
1.12
8.0
7.0
6.0
5.0
4.0
3.0
2.0
1.0
0.0
ppm (t1)

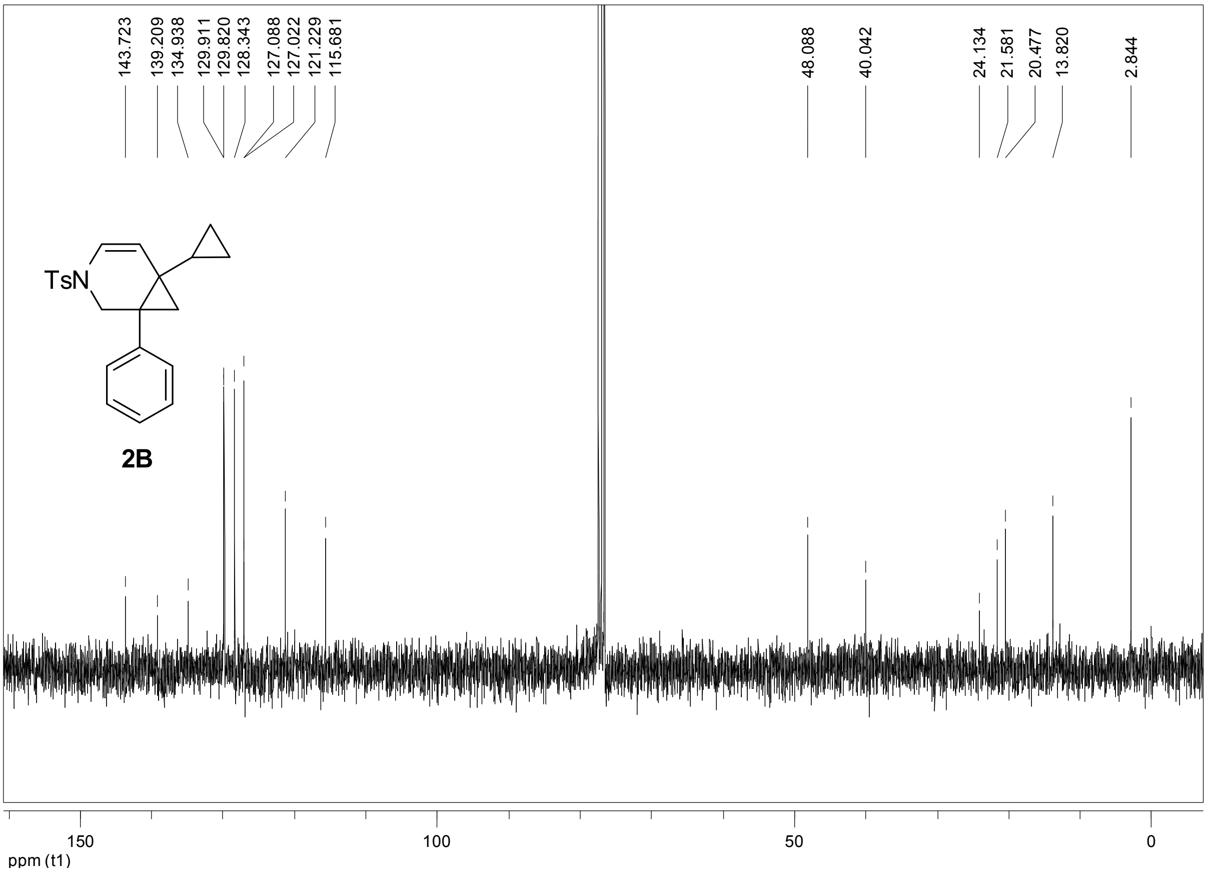
143.723
139.209
134.938
129.911
129.820
128.343
127.088
127.022
121.229
115.681
48.088
40.042
24.134
21.581
20.477
13.820
2.844
TsN
2B
150
100
50
0
ppm (t1)

3B

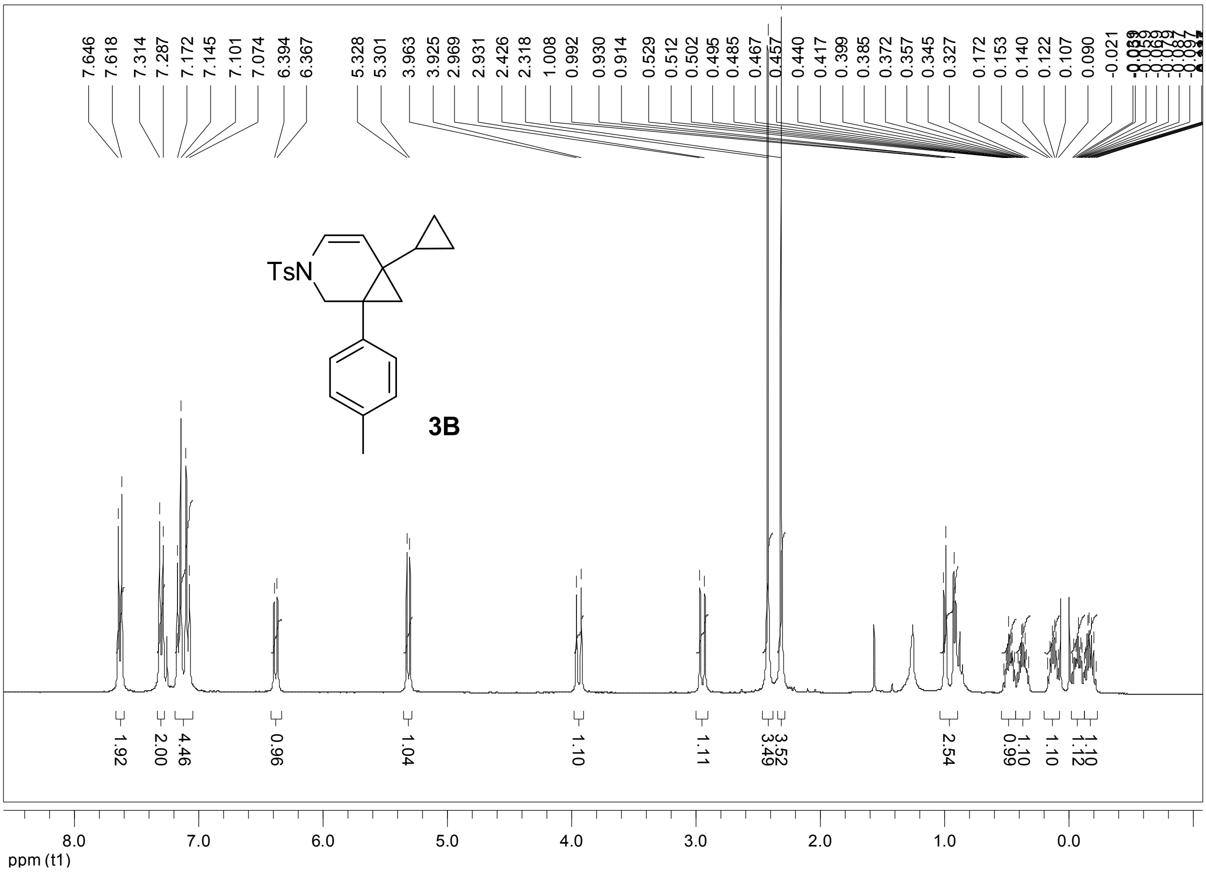
7.646
7.618
7.314
7.287
7.172
7.145
7.101
7.074
6.394
6.367
5.328
5.301
3.963
3.925
2.969
2.931
2.426
2.318
1.008
0.992
0.930
0.914
0.529
0.512
0.502
0.495
0.485
0.467
0.457
0.440
0.417
0.399
0.385
0.372
0.357
0.345
0.327
0.172
0.153
0.140
0.122
0.107
0.090
-0.021
TsN
3B
1.92
2.00
4.46
0.96
1.04
1.10
1.11
3.49
3.52
2.54
0.99
1.10
1.10
1.12
1.10
8.0
7.0
6.0
5.0
4.0
3.0
2.0
1.0
0.0
ppm (t1)

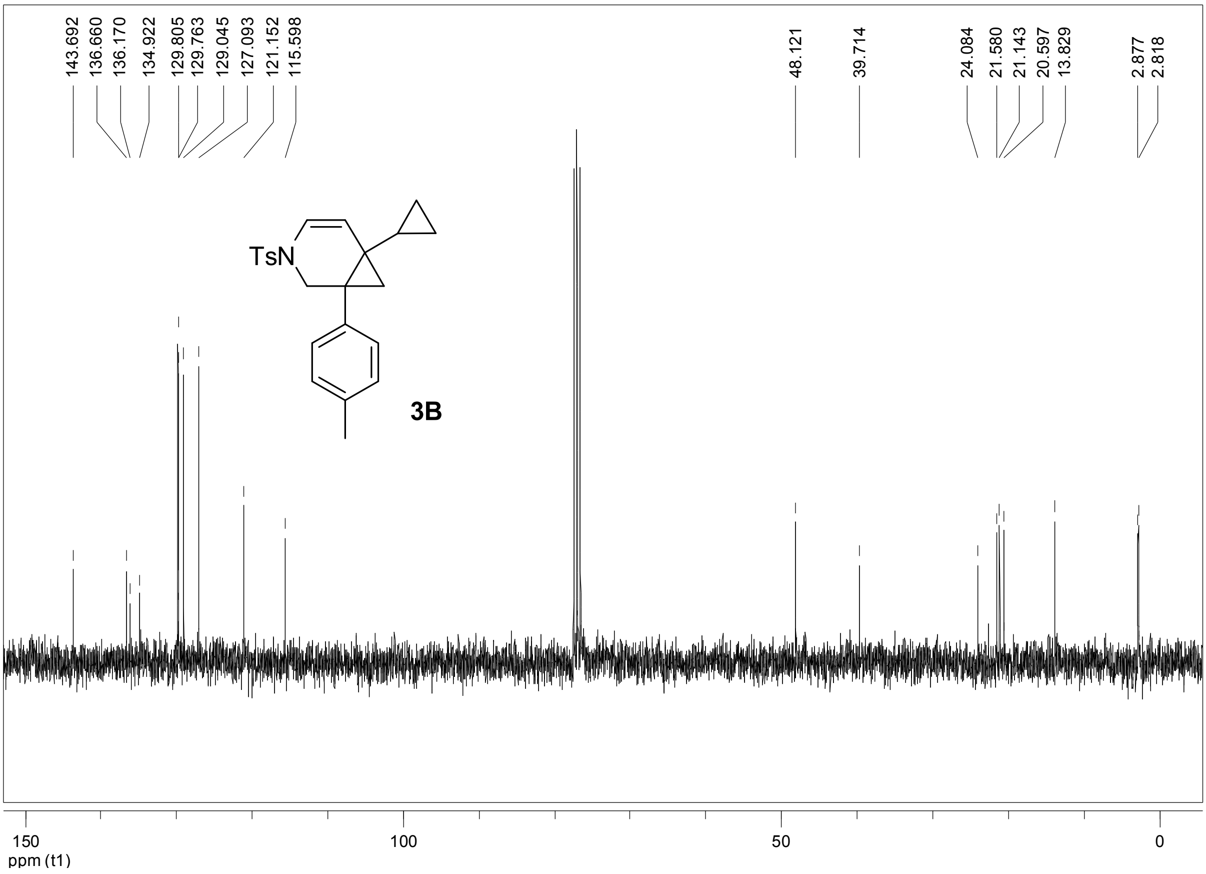
143.692
136.660
136.170
134.922
129.805
129.763
129.045
127.093
121.152
115.598
48.121
39.714
24.084
21.580
21.143
20.597
13.829
2.877
2.818
TsN
3B
150
100
50
0
ppm (t1)

4B

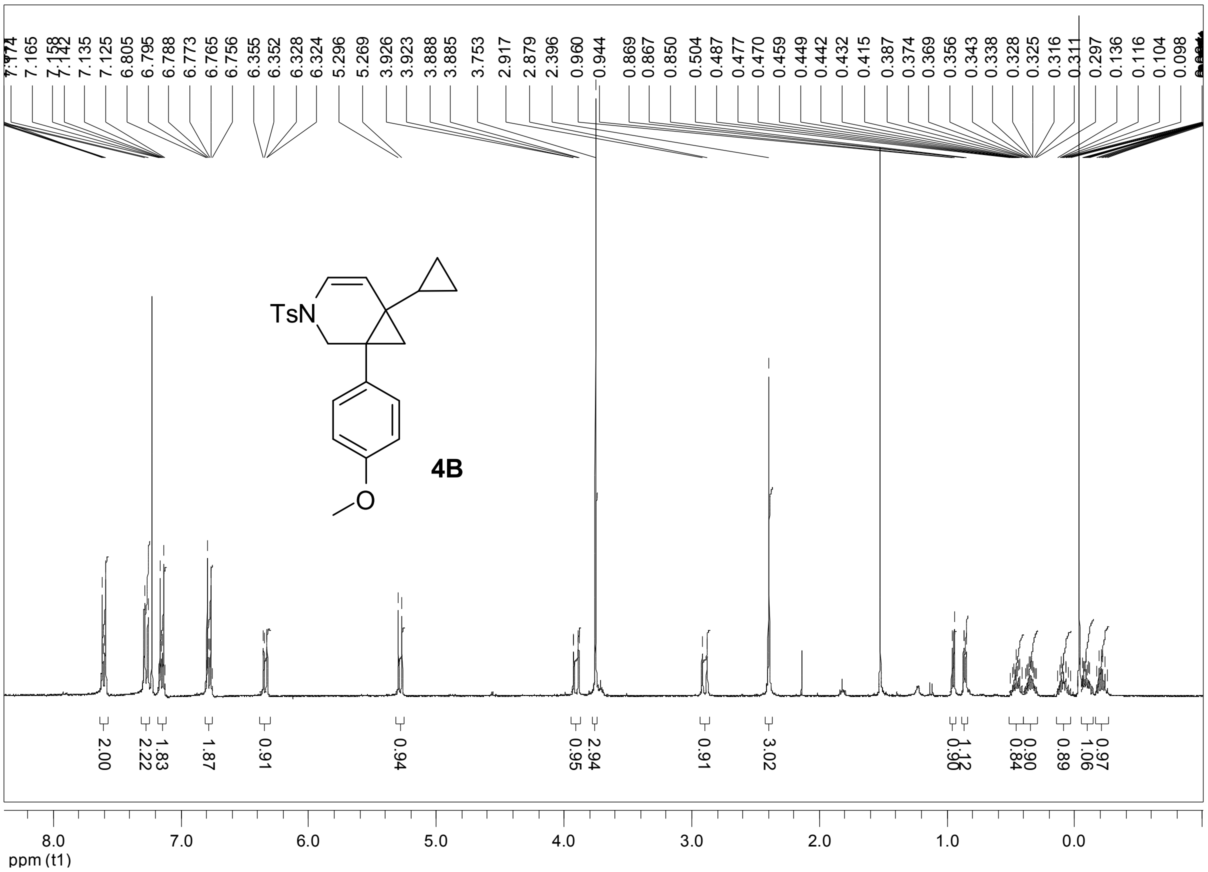

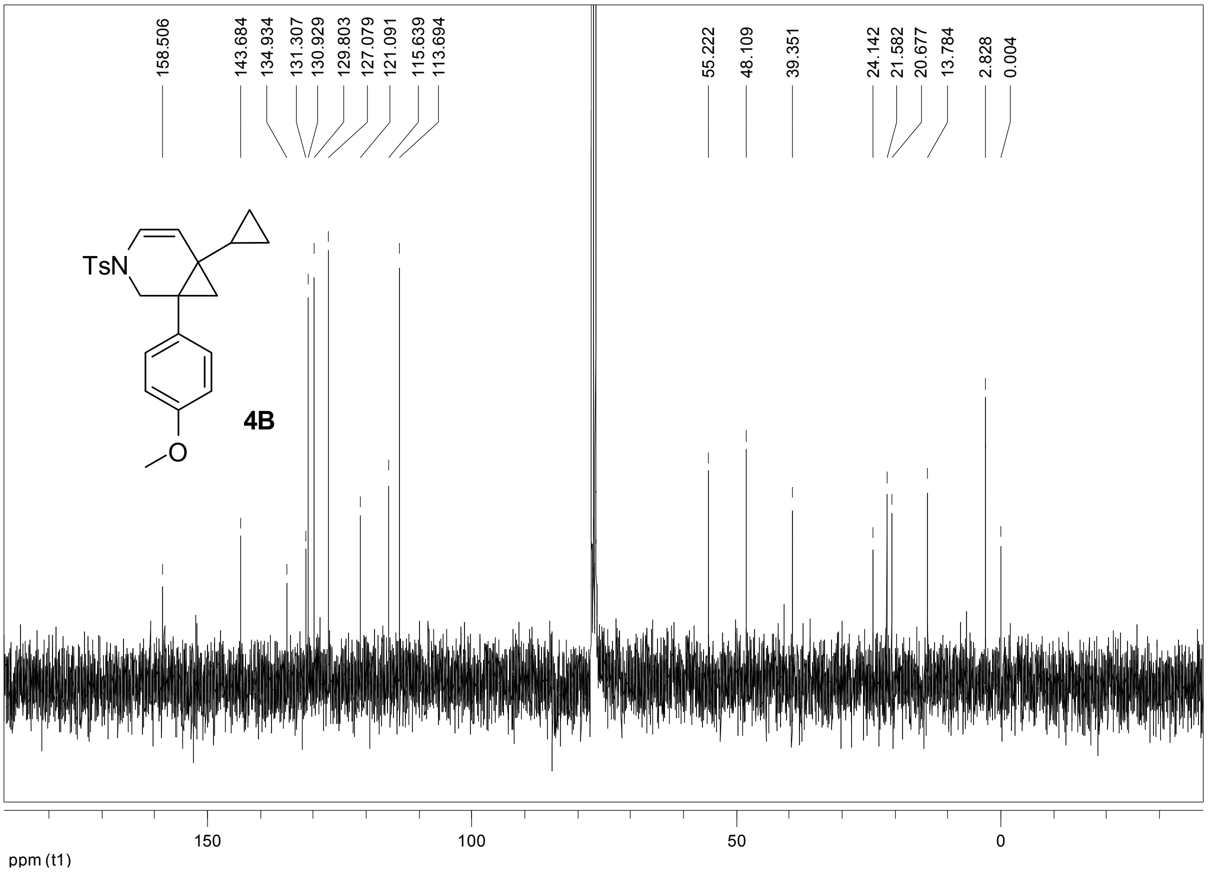

5B

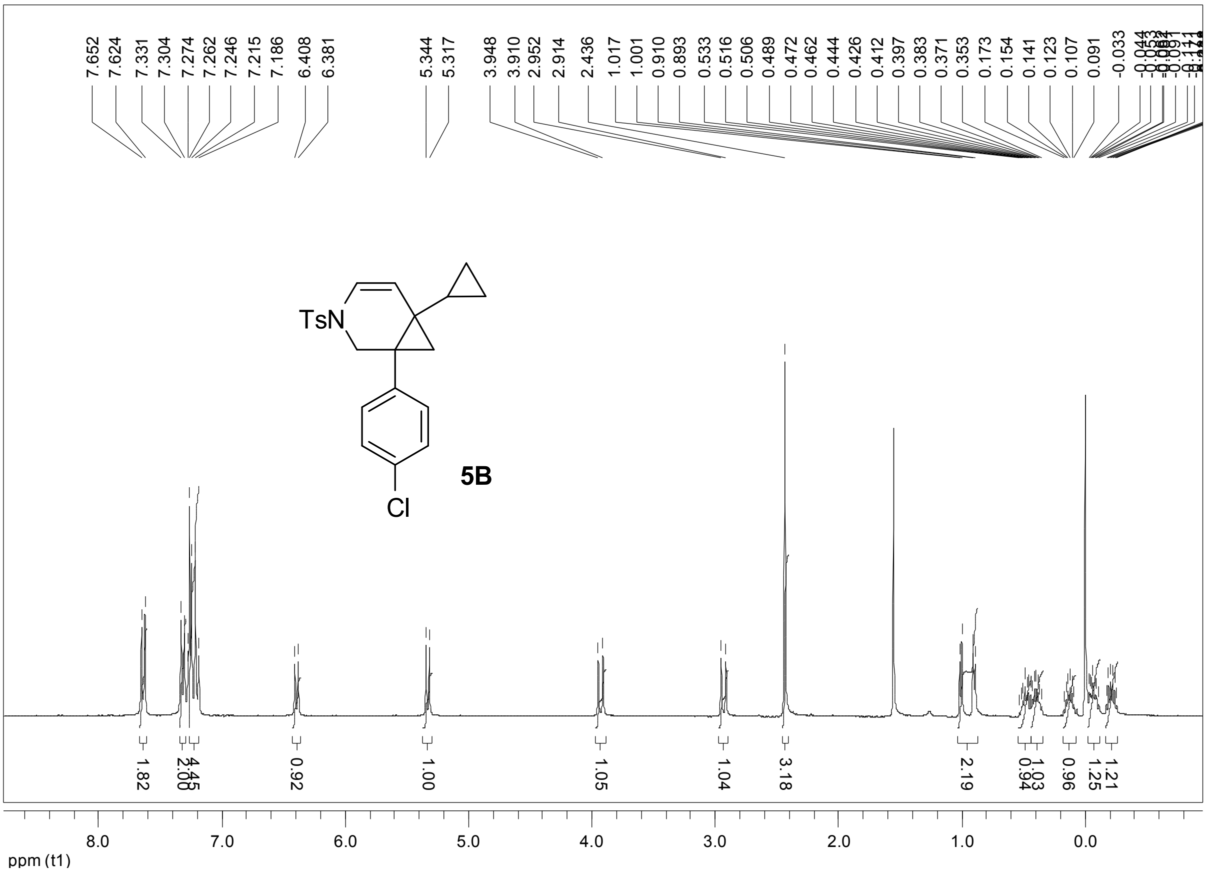
7.652
7.624
7.331
7.304
7.274
7.262
7.246
7.215
7.186
6.408
6.381
5.344
5.317
3.948
3.910
2.952
2.914
2.436
1.017
1.001
0.910
0.893
0.533
0.516
0.506
0.489
0.472
0.462
0.444
0.426
0.412
0.397
0.383
0.371
0.353
0.173
0.154
0.141
0.123
0.107
0.091
-0.033
TsN
Cl
5B
1.82
2.00
4.45
0.92
1.00
1.05
1.04
3.18
2.19
0.94
1.03
0.96
1.25
1.21
8.0
7.0
6.0
5.0
4.0
3.0
2.0
1.0
0.0
ppm (t1)

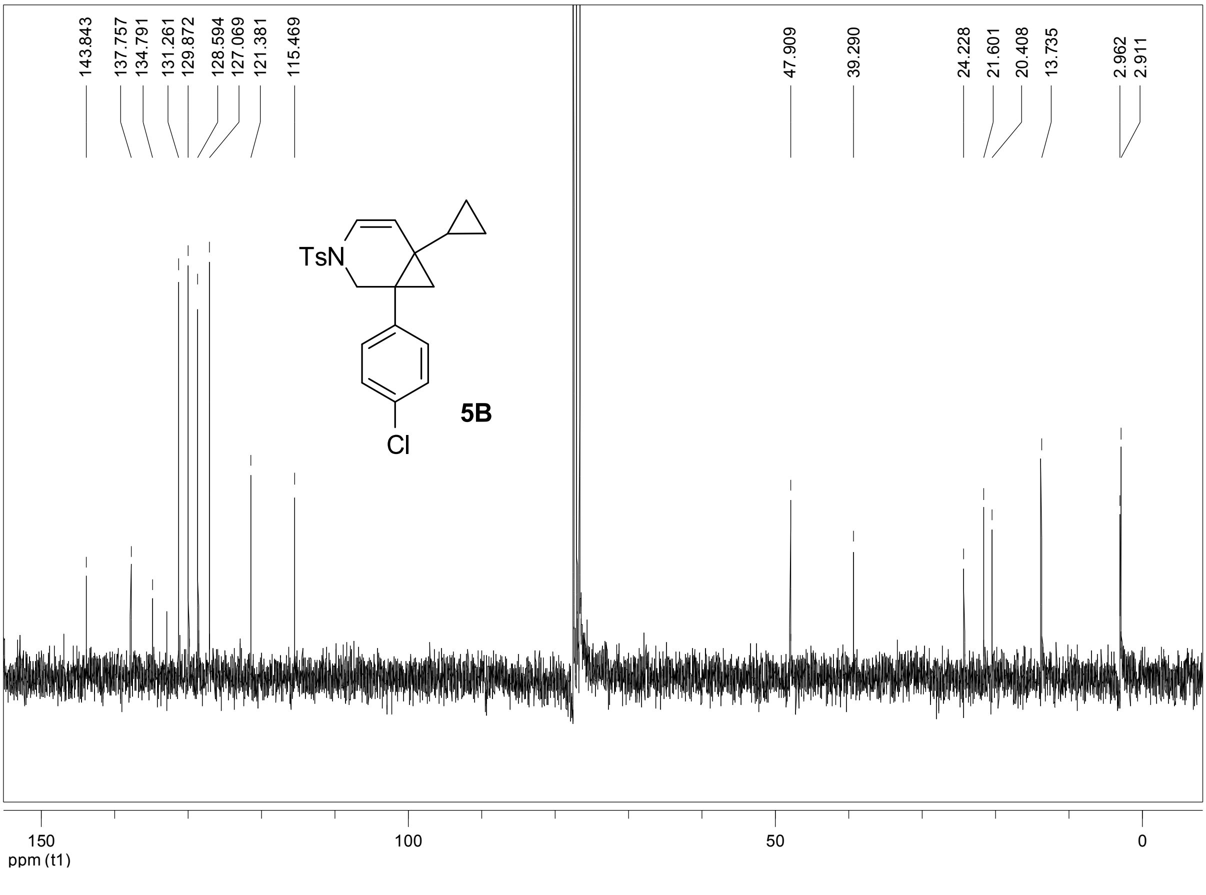
143.843
137.757
134.791
131.261
129.872
128.594
127.069
121.381
115.469
47.909
39.290
24.228
21.601
20.408
13.735
2.962
2.911
TsN
Cl
5B
150
100
50
0
ppm (t1)

6B

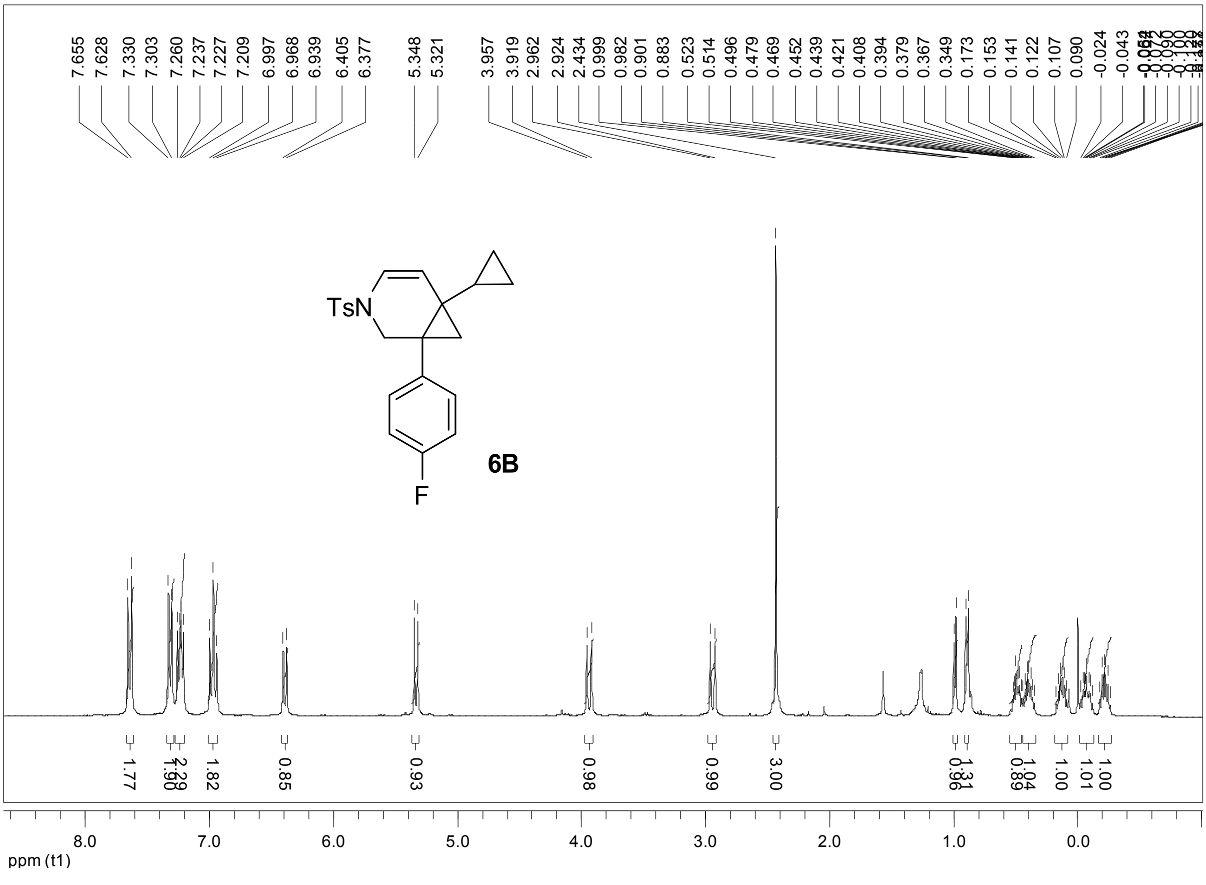
7.655
7.628
7.330
7.303
7.260
7.237
7.227
7.209
6.997
6.968
6.939
6.405
6.377
5.348
5.321
3.957
3.919
2.962
2.924
2.434
0.999
0.982
0.901
0.883
0.523
0.514
0.496
0.479
0.469
0.452
0.439
0.421
0.408
0.394
0.379
0.367
0.349
0.173
0.153
0.141
0.122
0.107
0.090
-0.024
-0.043
TsN
F
6B
1.77
1.90
2.29
1.82
0.85
0.93
0.98
0.99
3.00
0.96
1.31
0.89
1.04
1.00
1.01
1.00
8.0
7.0
6.0
5.0
4.0
3.0
2.0
1.0
0.0
ppm (t1)

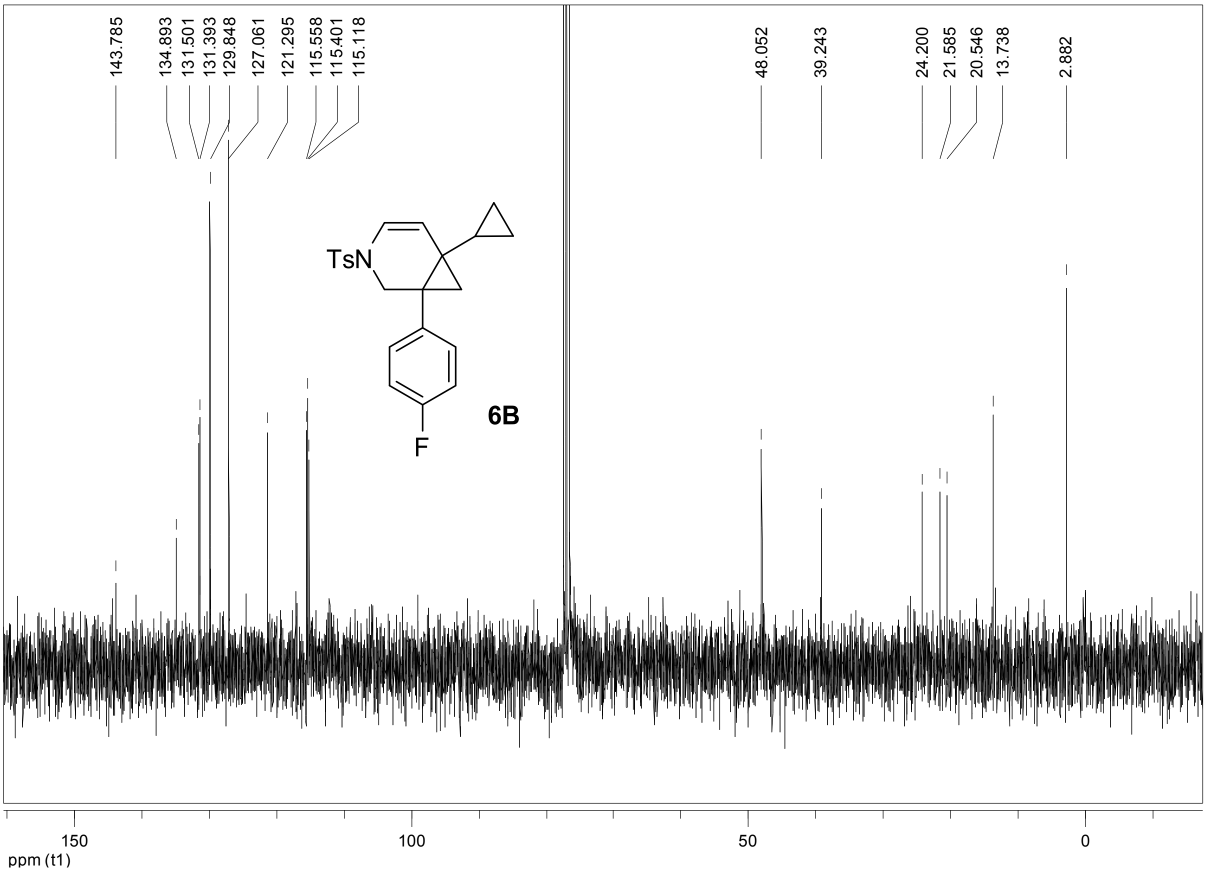
143.785
134.893
131.501
131.393
129.848
127.061
121.295
115.558
115.401
115.118
48.052
39.243
24.200
21.585
20.546
13.738
2.882
TsN
F
6B
150
100
50
0
ppm (t1)

7B

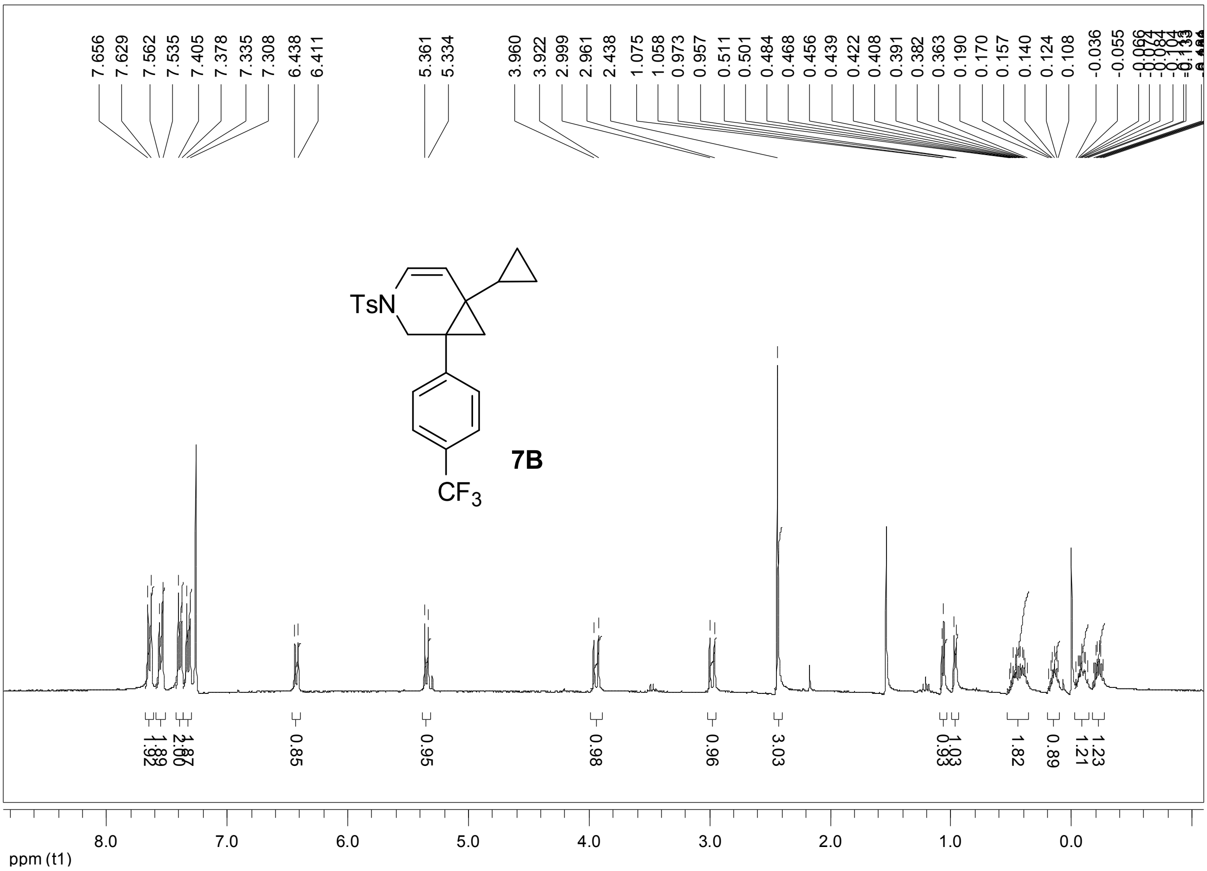
7.656
7.629
7.562
7.535
7.405
7.378
7.335
7.308
6.438
6.411
5.361
5.334
3.960
3.922
2.999
2.961
2.438
1.075
1.058
0.973
0.957
0.511
0.501
0.484
0.468
0.456
0.439
0.422
0.408
0.391
0.382
0.363
0.190
0.170
0.157
0.140
0.124
0.108
-0.036
-0.055
TsN
CF3
7B
0.85
0.95
0.98
0.96
3.03
1.82
0.89
1.21
1.23
8.0
7.0
6.0
5.0
4.0
3.0
2.0
1.0
0.0
ppm (t1)

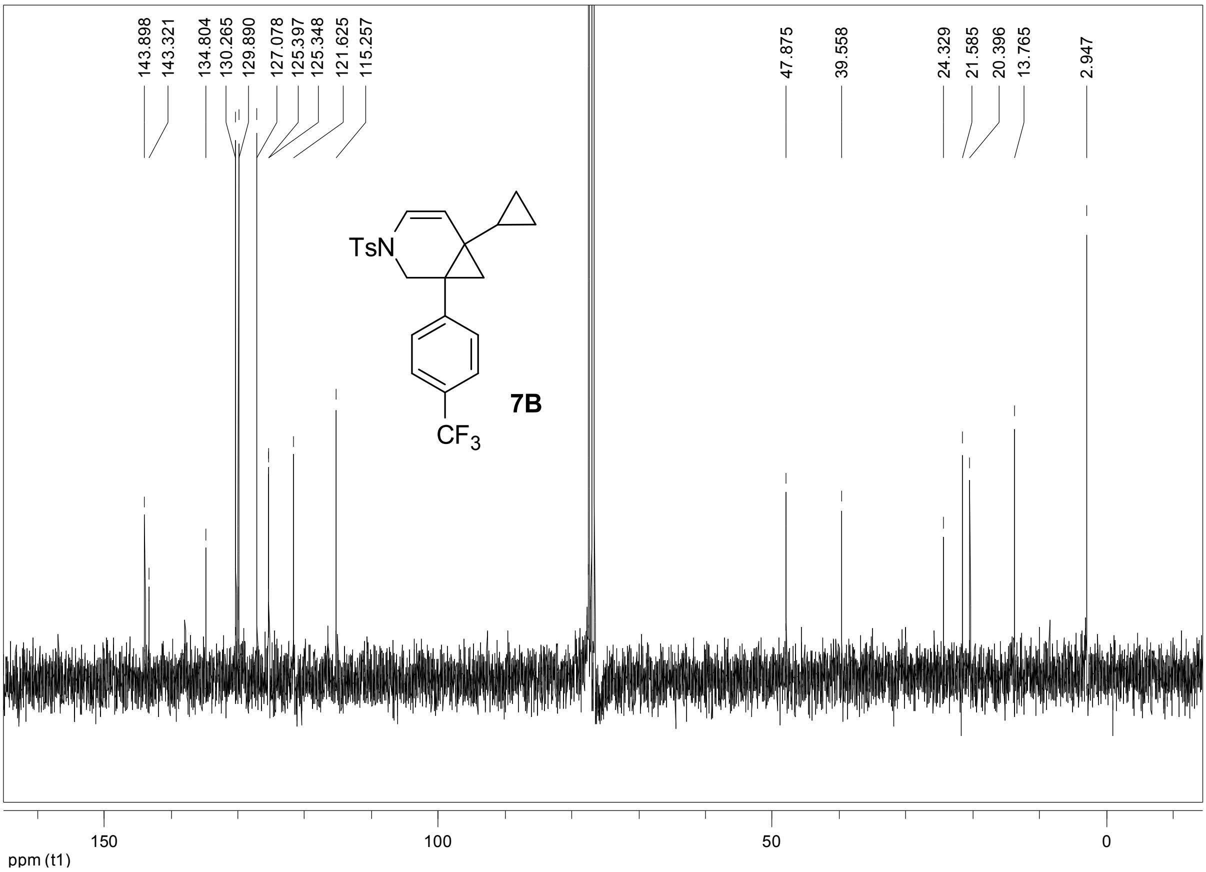
143.898
143.321
134.804
130.265
129.890
127.078
125.397
125.348
121.625
115.257
47.875
39.558
24.329
21.585
20.396
13.765
2.947
TsN
CF3
7B
150
100
50
0
ppm (t1)

8B

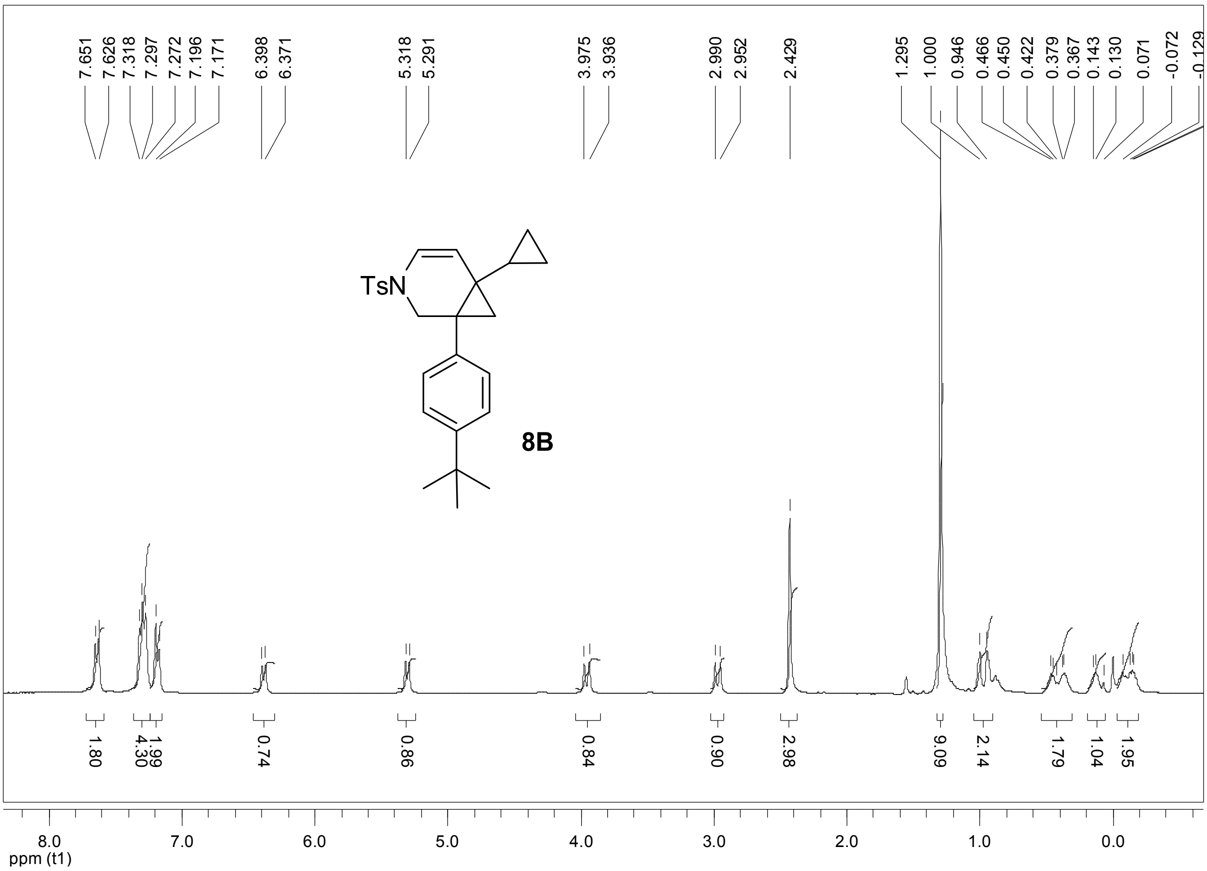

7.651
7.626
7.318
7.297
7.272
7.196
7.171
6.398
6.371
5.318
5.291
3.975
3.936
2.990
2.952
2.429
1.295
1.000
0.946
0.466
0.450
0.422
0.379
0.367
0.143
0.130
0.071
-0.072
-0.129
TsN
8B
1.80
4.30
1.99
0.74
0.86
0.84
0.90
2.98
9.09
2.14
1.79
1.04
1.95
8.0
7.0
6.0
5.0
4.0
3.0
2.0
1.0
0.0
ppm (t1)

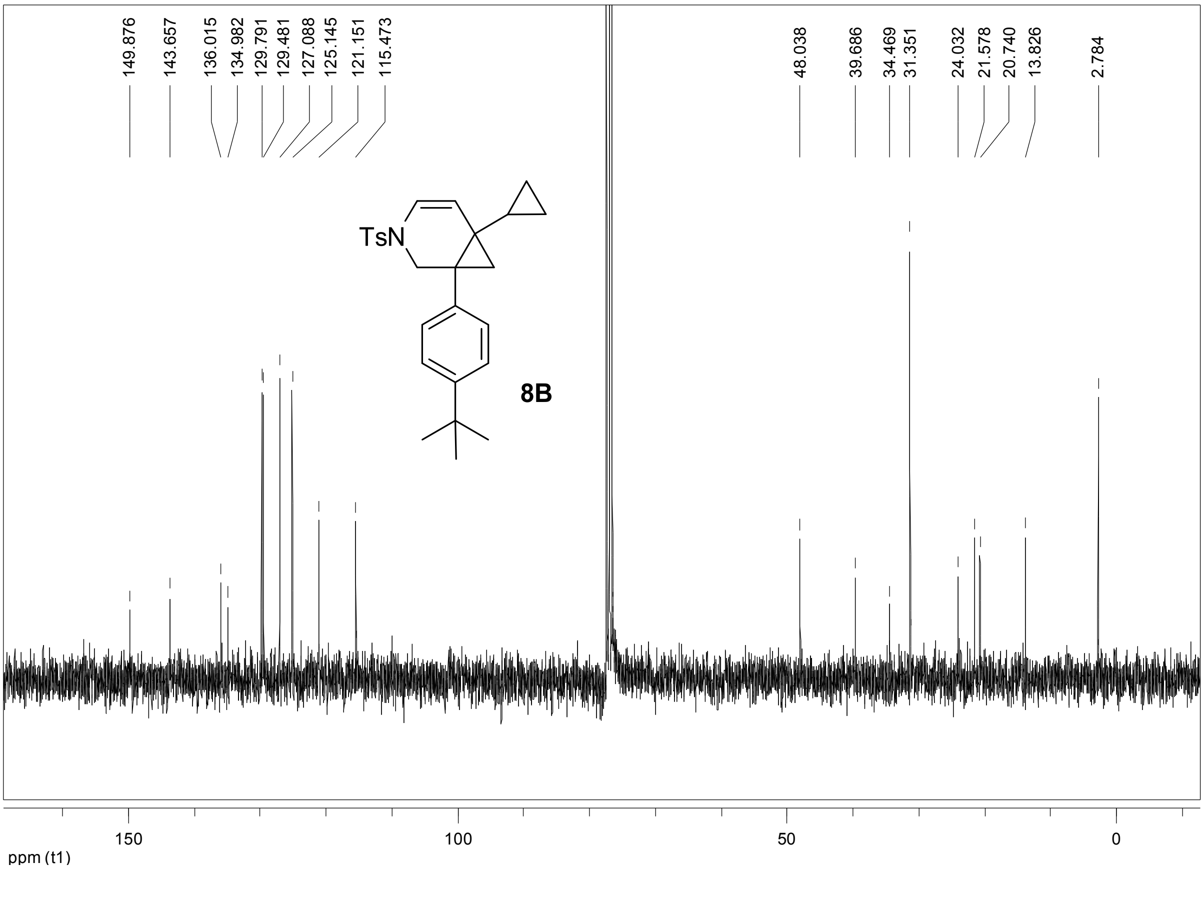

149.876
143.657
136.015
134.982
129.791
129.481
127.088
125.145
121.151
115.473
48.038
39.686
34.469
31.351
24.032
21.578
20.740
13.826
2.784
TsN
8B
150
100
50
0
ppm (t1)

9B

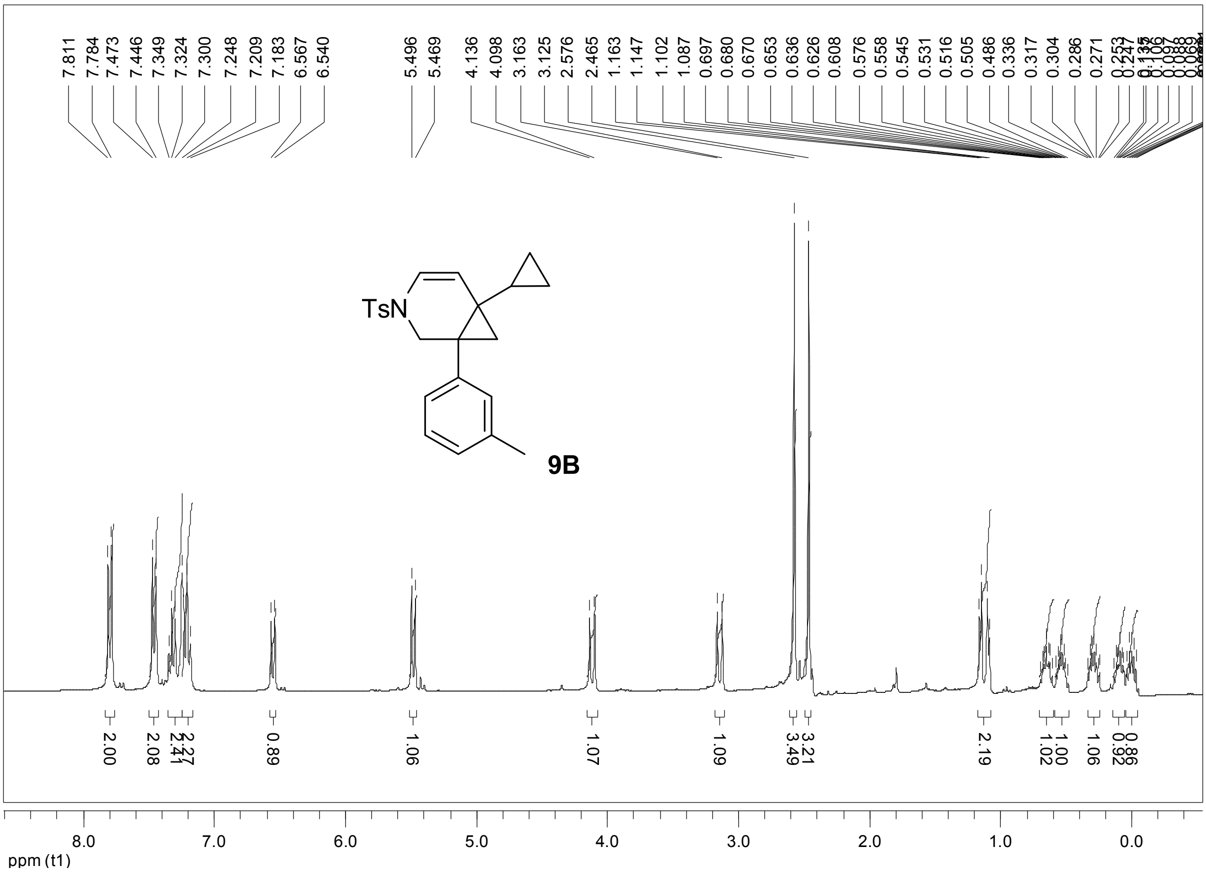
7.811
7.784
7.473
7.446
7.349
7.324
7.300
7.248
7.209
7.183
6.567
6.540
5.496
5.469
4.136
4.098
3.163
3.125
2.576
2.465
1.163
1.147
1.102
1.087
0.697
0.680
0.670
0.653
0.636
0.626
0.608
0.576
0.558
0.545
0.531
0.516
0.505
0.486
0.336
0.317
0.304
0.286
0.271
0.253
0.247
TsN
9B
2.00
2.08
2.41
2.27
0.89
1.06
1.07
1.09
3.49
3.21
2.19
1.02
1.00
1.06
0.92
0.86
8.0
7.0
6.0
5.0
4.0
3.0
2.0
1.0
0.0
ppm (t1)

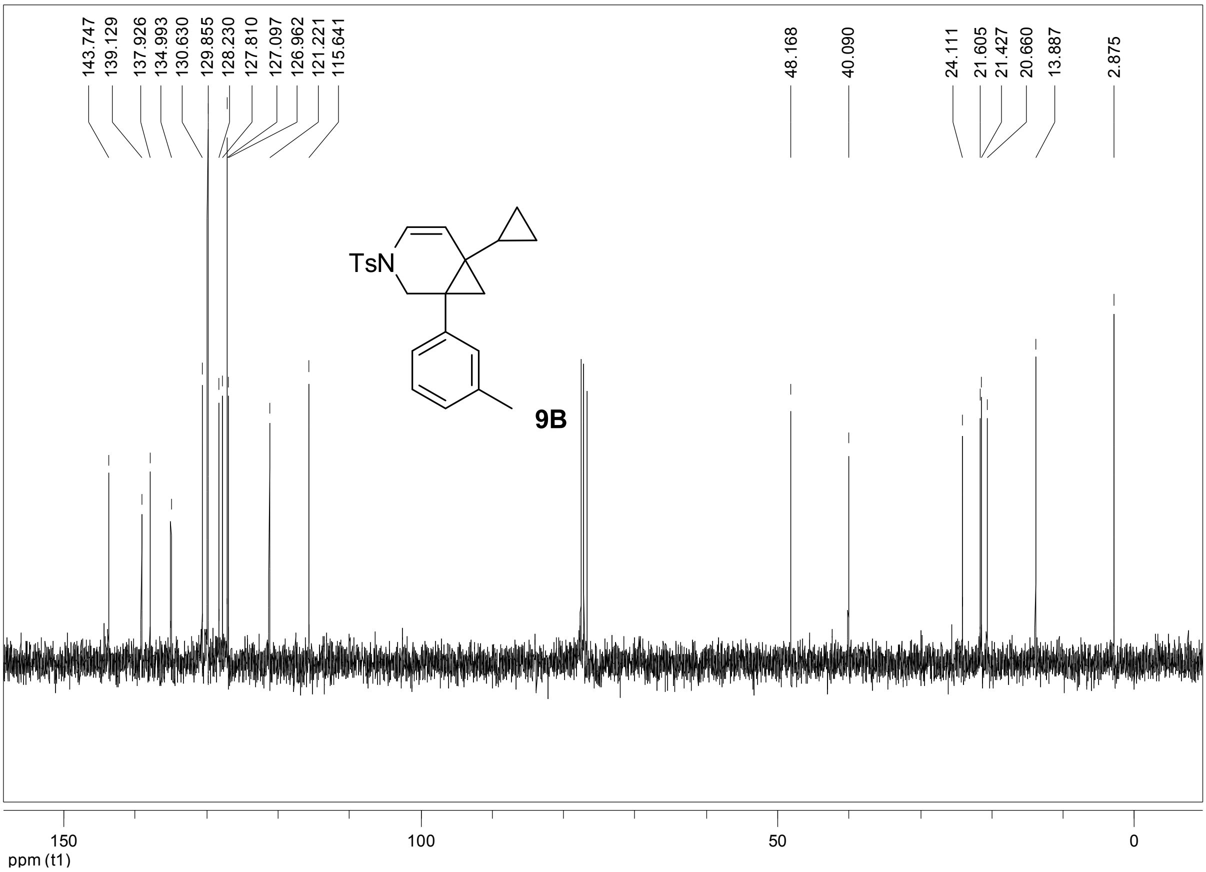
143.747
139.129
137.926
134.993
130.630
129.855
128.230
127.810
127.097
126.962
121.221
115.641
48.168
40.090
24.111
21.605
21.427
20.660
13.887
2.875
TsN
9B
150
100
50
0
ppm (t1)

10B

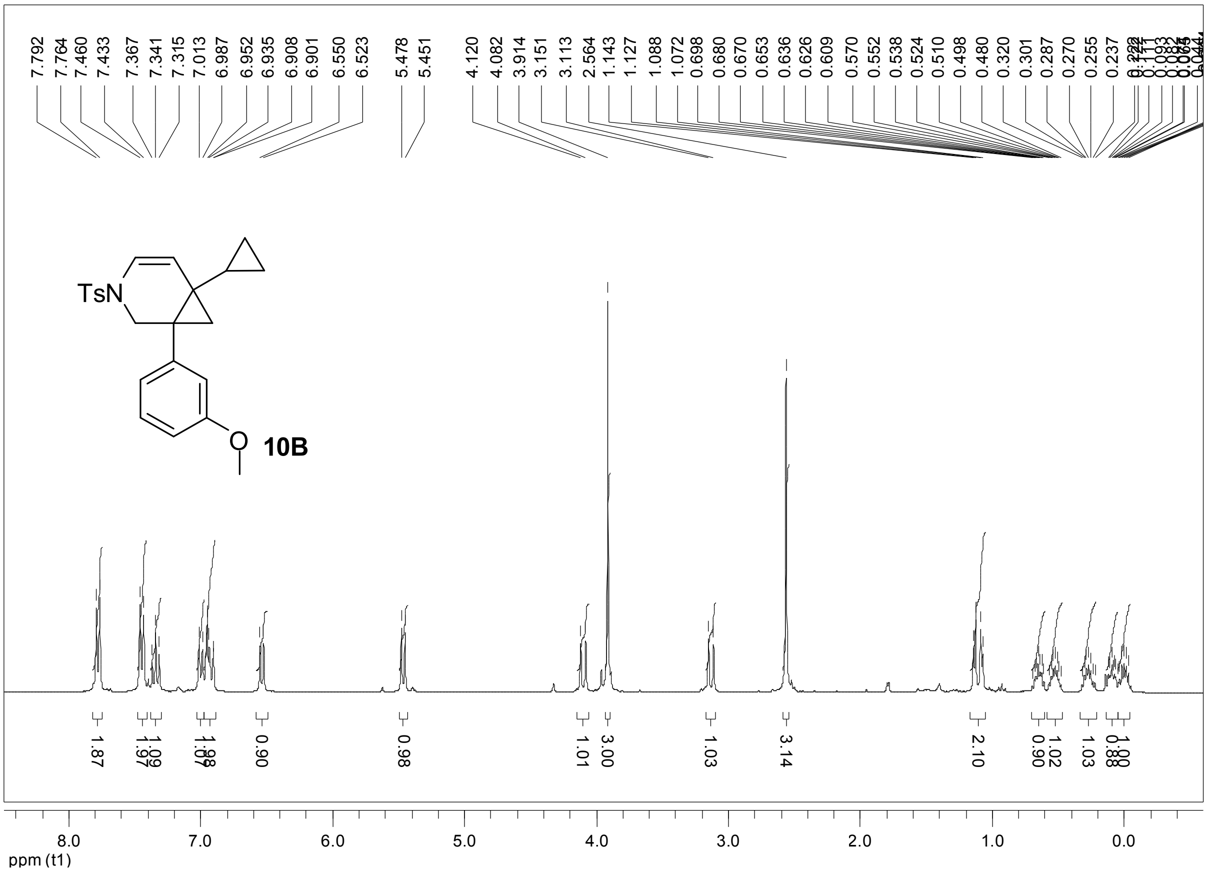
7.792
7.764
7.460
7.433
7.367
7.341
7.315
7.013
6.987
6.952
6.935
6.908
6.901
6.550
6.523
5.478
5.451
4.120
4.082
3.914
3.151
3.113
2.564
1.143
1.127
1.088
1.072
0.698
0.680
0.670
0.653
0.636
0.626
0.609
0.570
0.552
0.538
0.524
0.510
0.498
0.480
0.320
0.301
0.287
0.270
0.255
0.237
TsN
10B
1.87
0.90
0.98
1.01
3.00
1.03
3.14
2.10
0.90
1.02
1.03
8.0
7.0
6.0
5.0
4.0
3.0
2.0
1.0
0.0
ppm (t1)

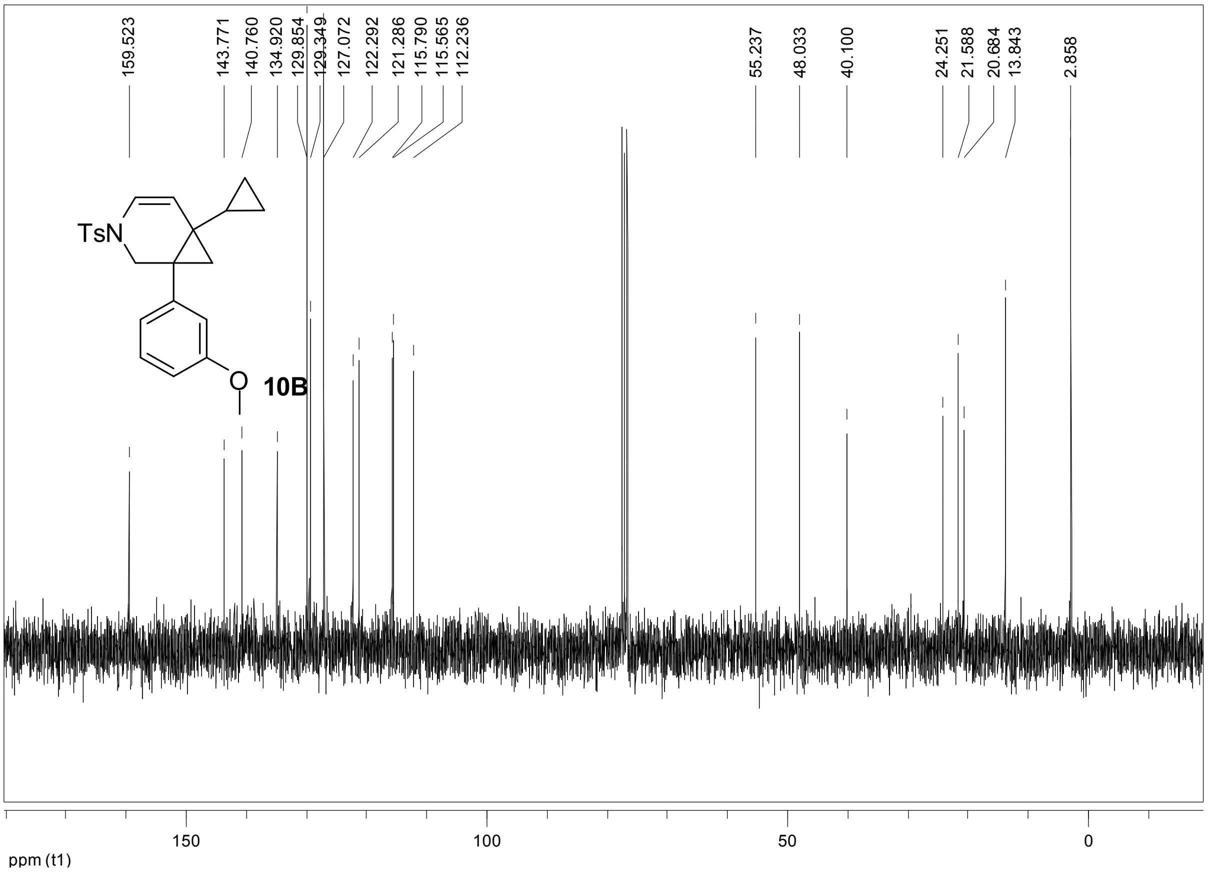
159.523
143.771
140.760
134.920
129.854
129.349
127.072
122.292
121.286
115.790
115.565
112.236
55.237
48.033
40.100
24.251
21.588
20.684
13.843
2.858
TsN
10B
150
100
50
0
ppm (t1)

11B

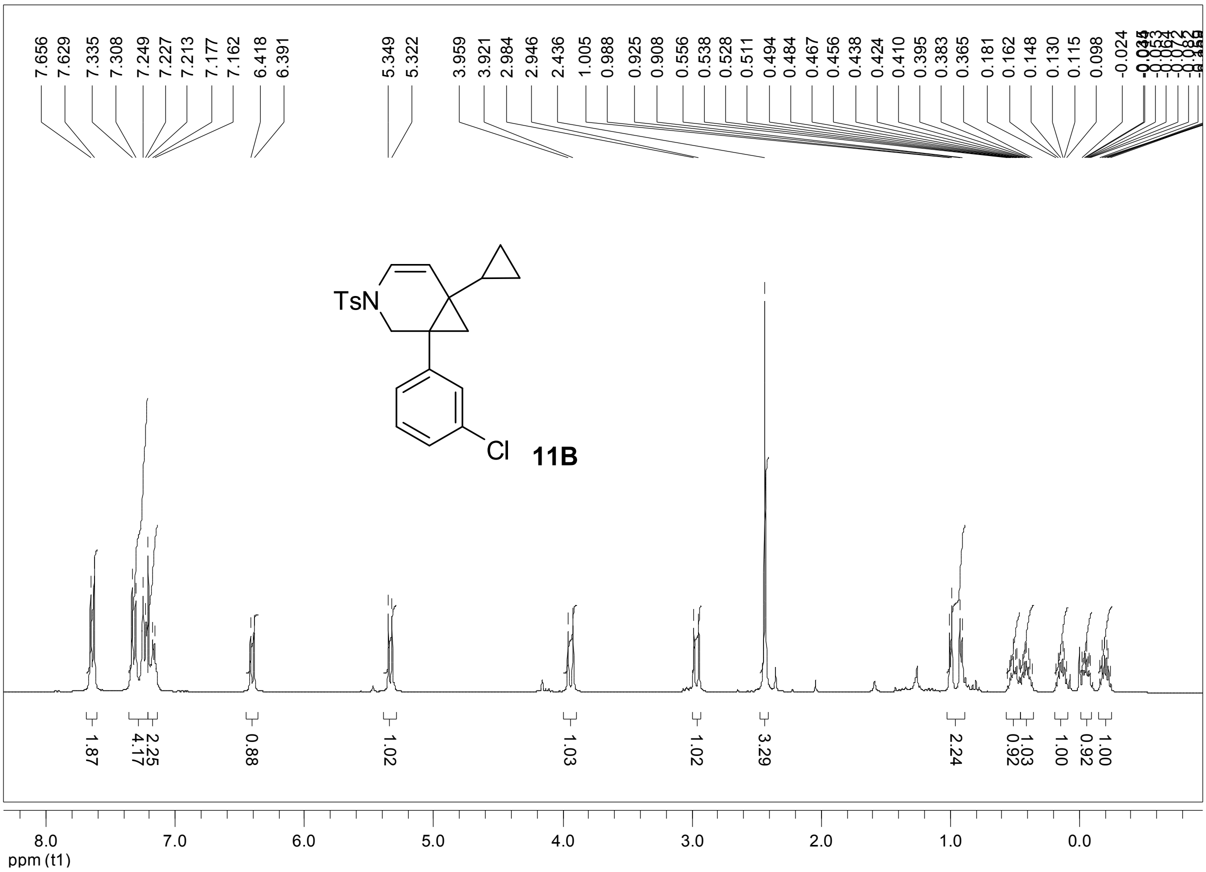
7.656
7.629
7.335
7.308
7.249
7.227
7.213
7.177
7.162
6.418
6.391
5.349
5.322
3.959
3.921
2.984
2.946
2.436
1.005
0.988
0.925
0.908
0.556
0.538
0.528
0.511
0.494
0.484
0.467
0.456
0.438
0.424
0.410
0.395
0.383
0.365
0.181
0.162
0.148
0.130
0.115
0.098
-0.024
TsN
Cl
11B
1.87
4.17
2.25
0.88
1.02
1.03
1.02
3.29
2.24
0.92
1.03
1.00
0.92
1.00
8.0
7.0
6.0
5.0
4.0
3.0
2.0
1.0
0.0
ppm (t1)

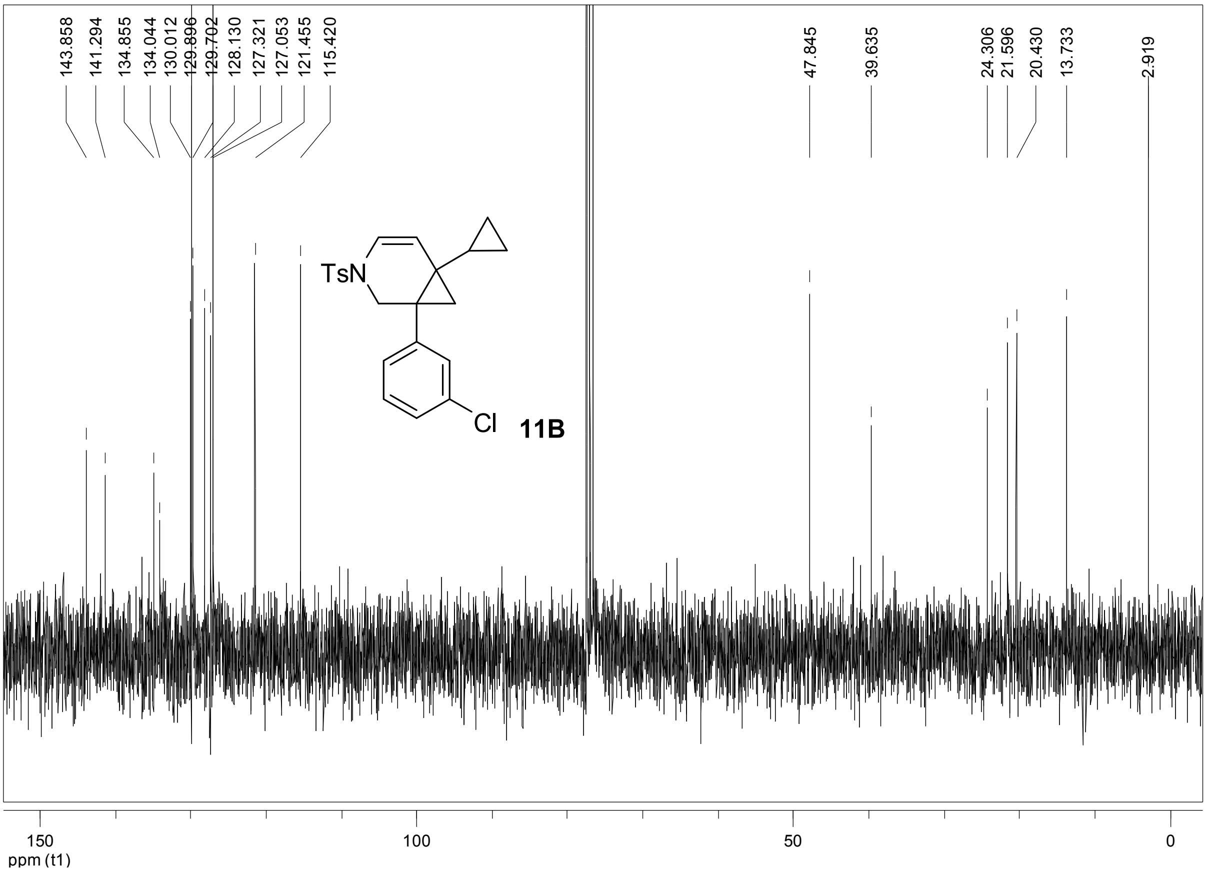
143.858
141.294
134.855
134.044
130.012
129.896
129.702
128.130
127.321
127.053
121.455
115.420
47.845
39.635
24.306
21.596
20.430
13.733
2.919
TsN
Cl
11B
150
100
50
0
ppm (t1)

12B

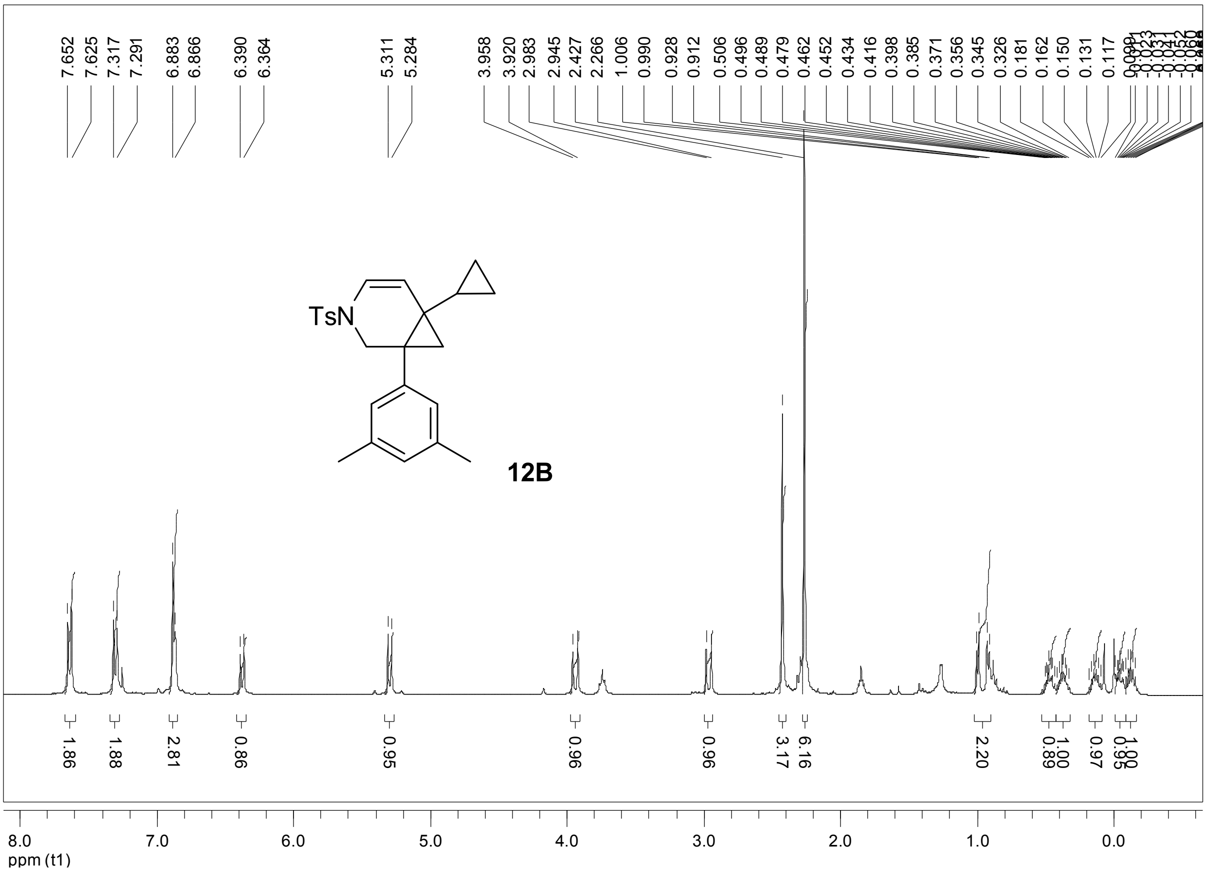
7.652
7.625
7.317
7.291
6.883
6.866
6.390
6.364
5.311
5.284
3.958
3.920
2.983
2.945
2.427
2.266
1.006
0.990
0.928
0.912
0.506
0.496
0.489
0.479
0.462
0.452
0.434
0.416
0.398
0.385
0.371
0.356
0.345
0.326
0.181
0.162
0.150
0.131
0.117
TsN
12B
1.86
1.88
2.81
0.86
0.95
0.96
0.96
3.17
6.16
2.20
0.89
1.00
0.97
0.95
1.00
8.0
7.0
6.0
5.0
4.0
3.0
2.0
1.0
0.0
ppm (t1)

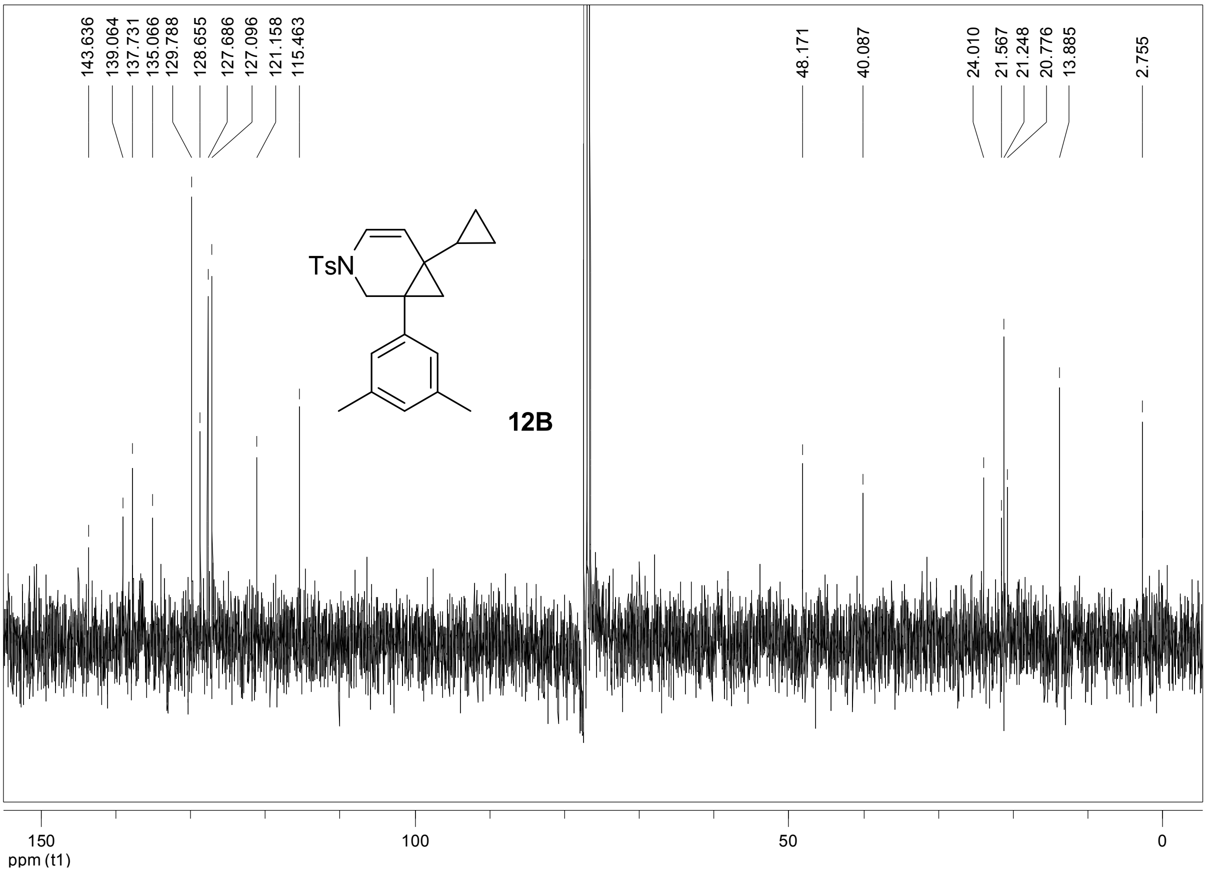
143.636
139.064
137.731
135.066
129.788
128.655
127.686
127.096
121.158
115.463
48.171
40.087
24.010
21.567
21.248
20.776
13.885
2.755
TsN
12B
150
100
50
0
ppm (t1)

13B

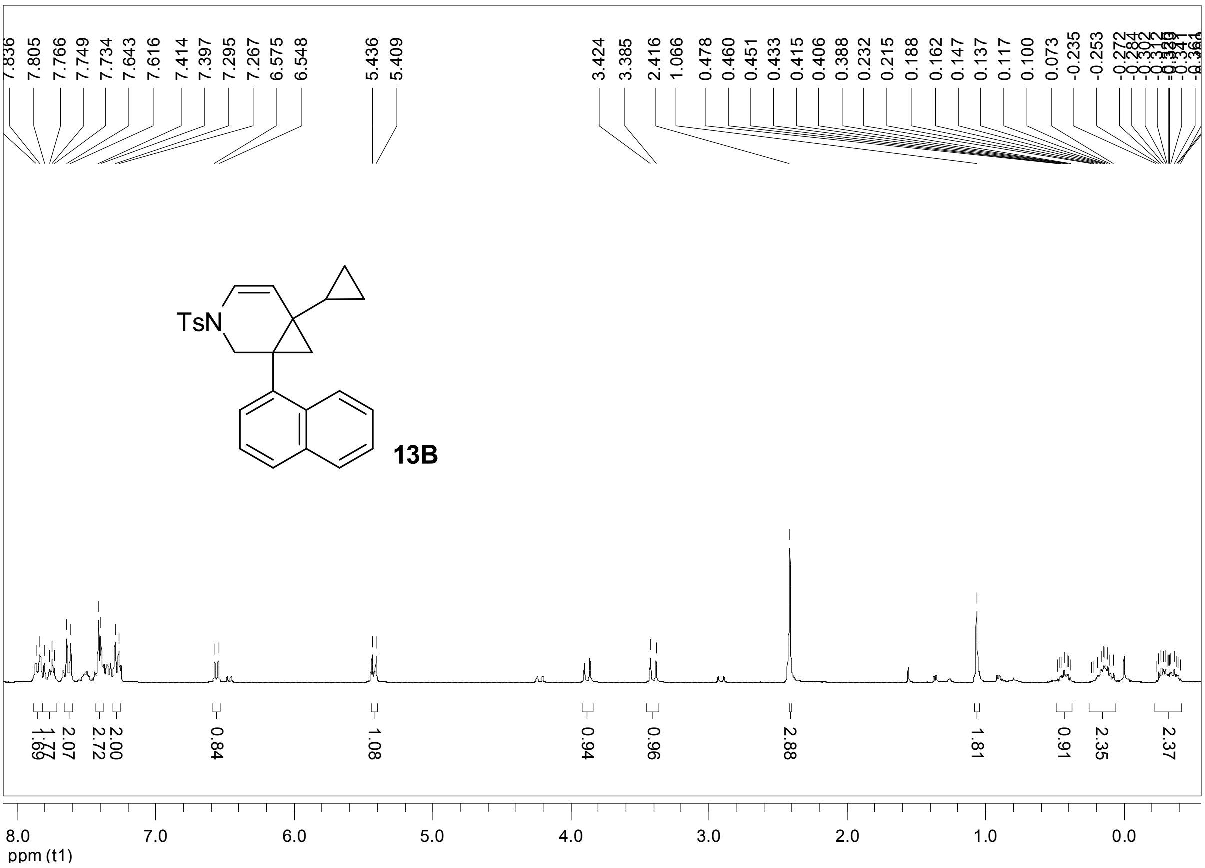
7.836
7.805
7.766
7.749
7.734
7.643
7.616
7.414
7.397
7.295
7.267
6.575
6.548
5.436
5.409
3.424
3.385
2.416
1.066
0.478
0.460
0.451
0.433
0.415
0.406
0.388
0.232
0.215
0.188
0.162
0.147
0.137
0.117
0.100
0.073
-0.235
-0.253
-0.272
TsN
13B
1.69
1.77
2.07
2.72
2.00
0.84
1.08
0.94
0.96
2.88
1.81
0.91
2.35
2.37
8.0
7.0
6.0
5.0
4.0
3.0
2.0
1.0
0.0
ppm (t1)

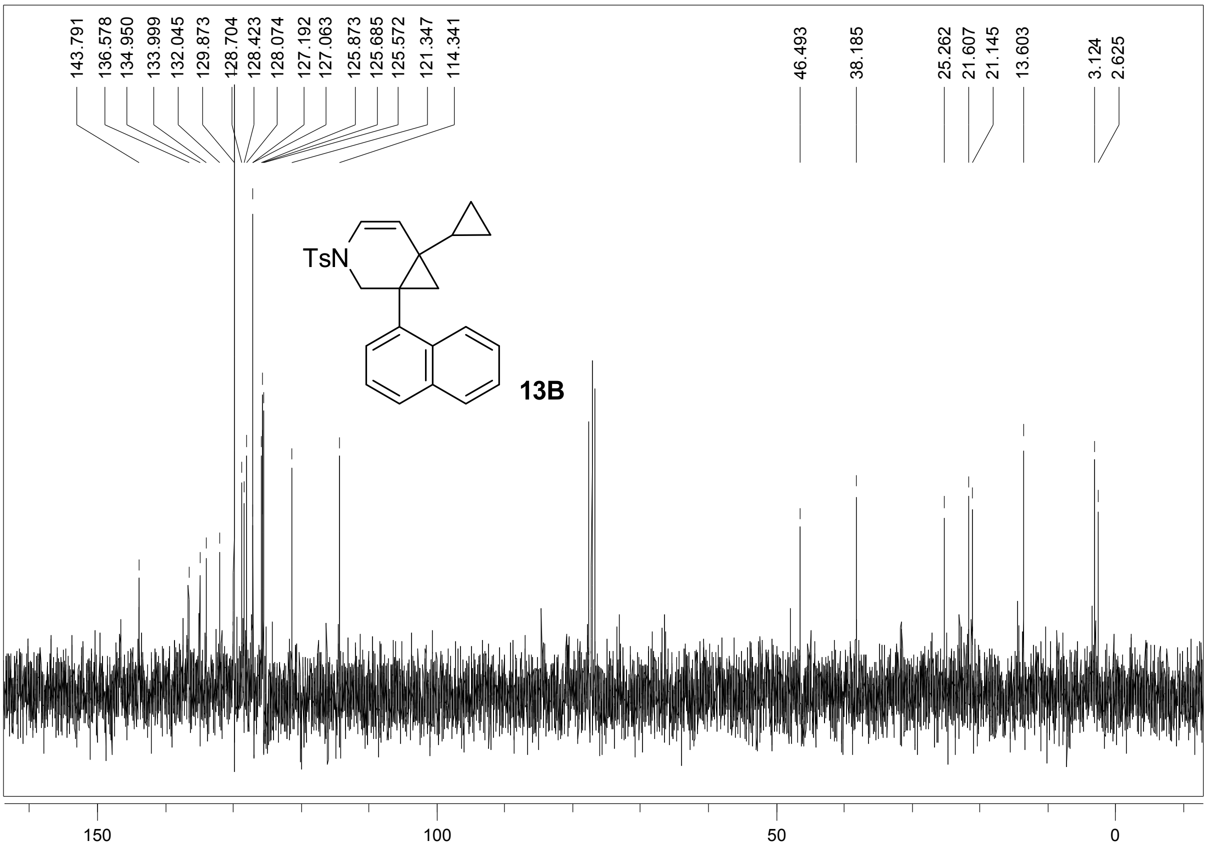
143.791
136.578
134.950
133.999
132.045
129.873
128.704
128.423
128.074
127.192
127.063
125.873
125.685
125.572
121.347
114.341
46.493
38.185
25.262
21.607
21.145
13.603
3.124
2.625
TsN
13B
150
100
50
0
ppm (t1)

14B

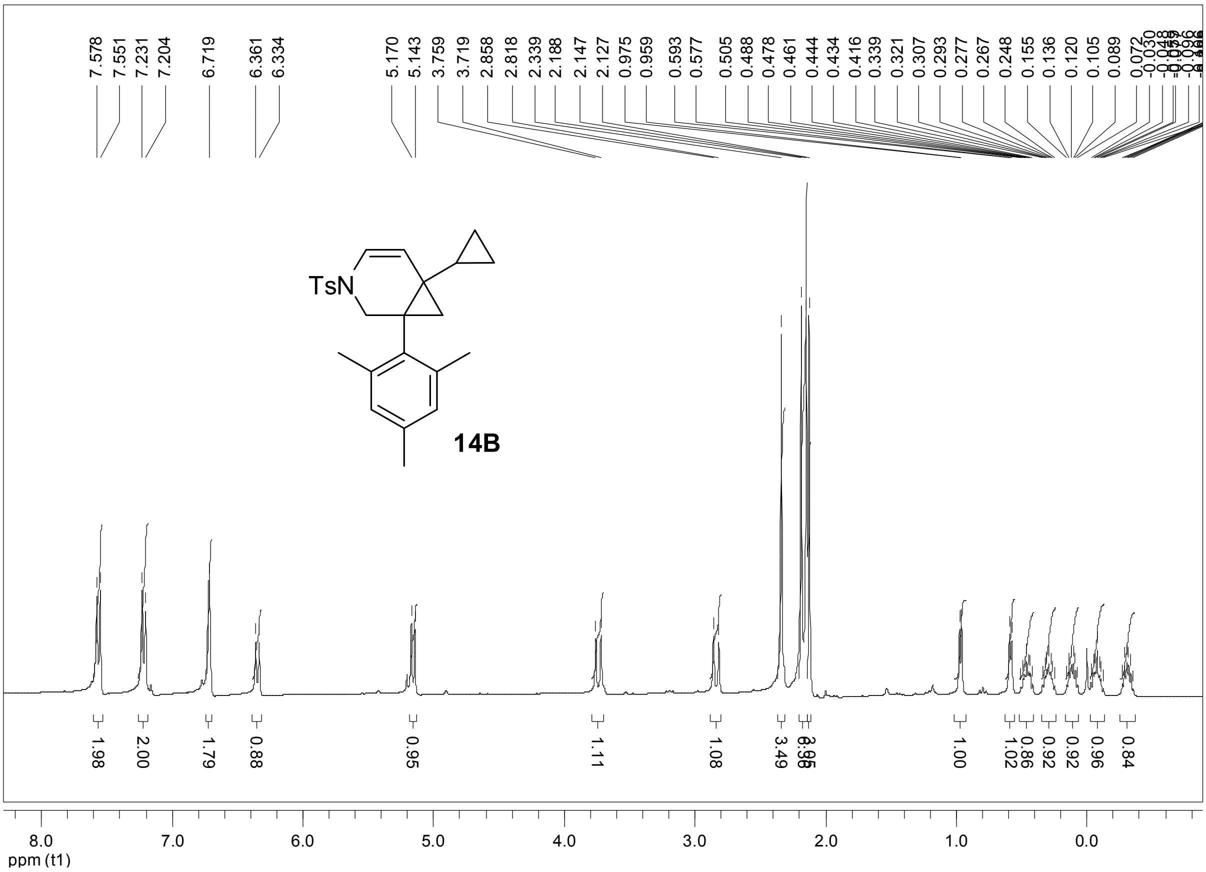
7.578
7.551
7.231
7.204
6.719
6.361
6.334
5.170
5.143
3.759
3.719
2.858
2.818
2.339
2.188
2.147
2.127
0.975
0.959
0.593
0.577
0.505
0.488
0.478
0.461
0.444
0.434
0.416
0.339
0.321
0.307
0.293
0.277
0.267
0.248
0.155
0.136
0.120
0.105
0.089
0.072
TsN
14B
1.98
2.00
1.79
0.88
0.95
1.11
1.08
3.49
1.00
1.02
0.86
0.92
0.92
0.96
0.84
8.0
7.0
6.0
5.0
4.0
3.0
2.0
1.0
0.0
ppm (t1)

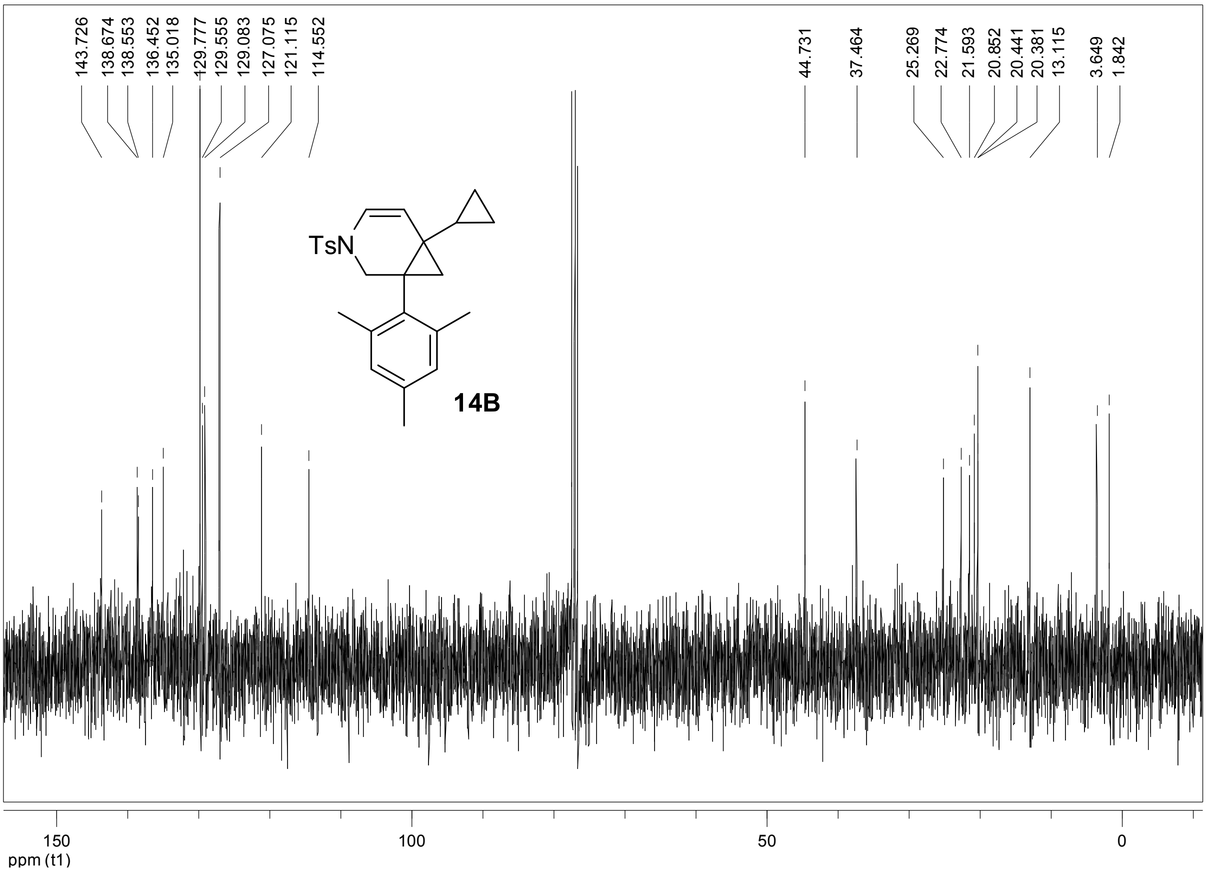
143.726
138.674
138.553
136.452
135.018
129.777
129.555
129.083
127.075
121.115
114.552
44.731
37.464
25.269
22.774
21.593
20.852
20.441
20.381
13.115
3.649
1.842
TsN
14B
150
100
50
0
ppm (t1)

15B

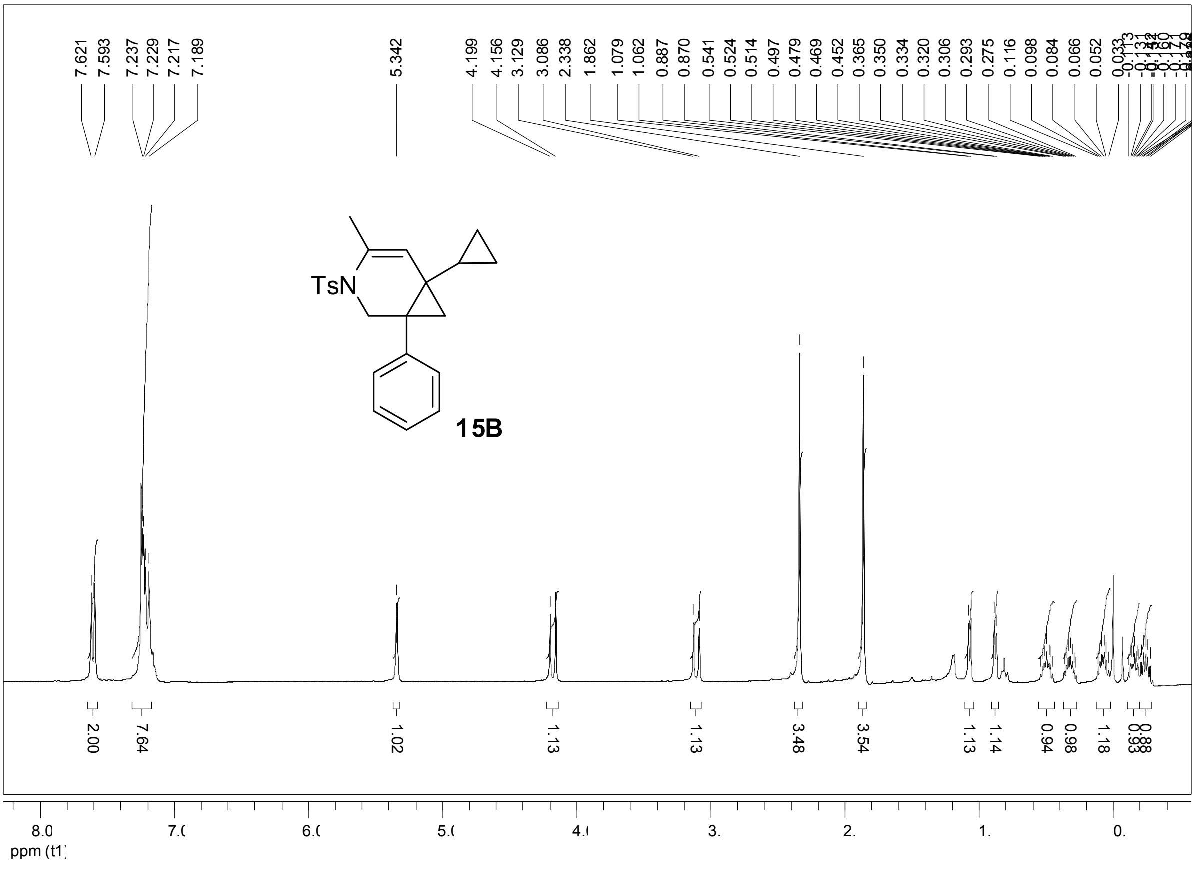
7.621
7.593
7.237
7.229
7.217
7.189
5.342
4.199
4.156
3.129
3.086
2.338
1.862
1.079
1.062
0.887
0.870
0.541
0.524
0.514
0.497
0.479
0.469
0.452
0.365
0.350
0.334
0.320
0.306
0.293
0.275
0.116
0.098
0.084
0.066
0.052
0.033
TsN
15B
2.00
7.64
1.02
1.13
1.13
3.48
3.54
1.13
1.14
0.94
0.98
1.18
ppm (t1)

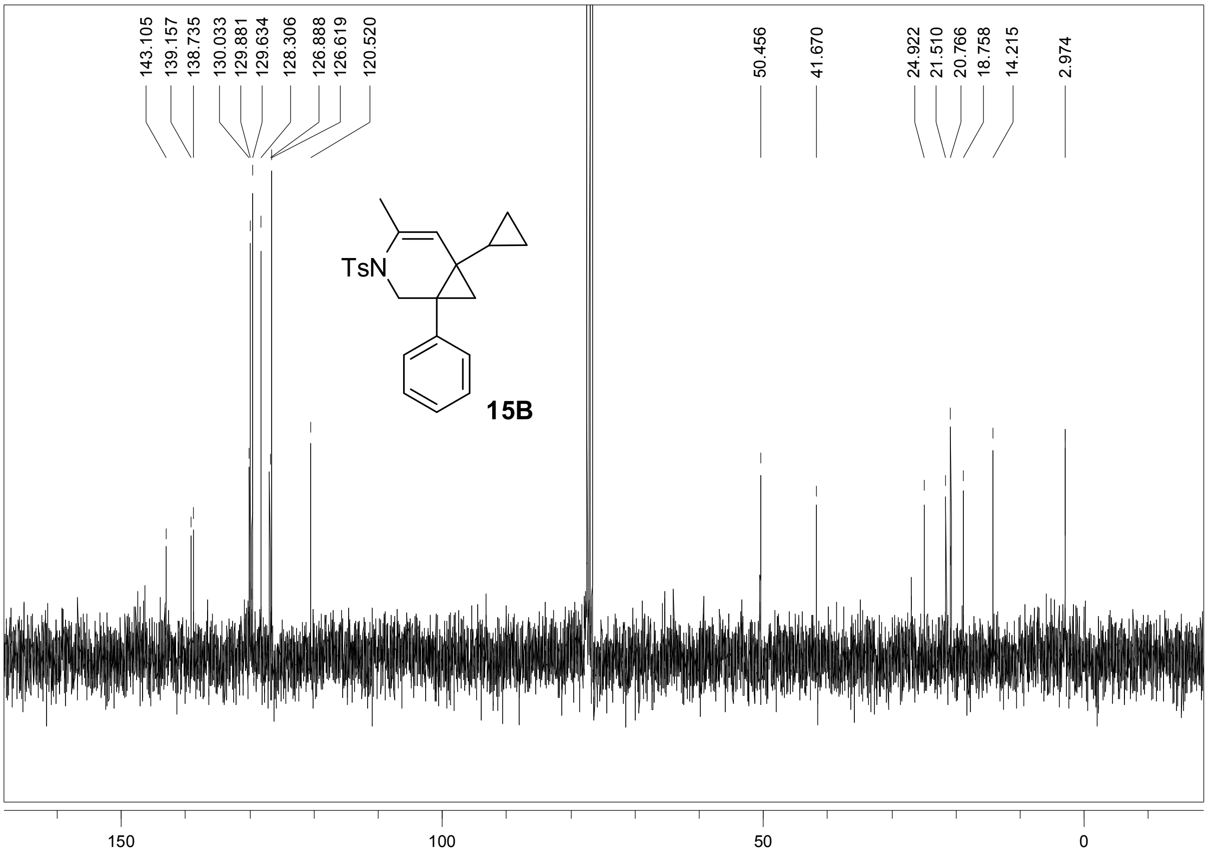
143.105
139.157
138.735
130.033
129.881
129.634
128.306
126.888
126.619
120.520
50.456
41.670
24.922
21.510
20.766
18.758
14.215
2.974
TsN
15B
150
100
50
0
ppm (t1)

16B

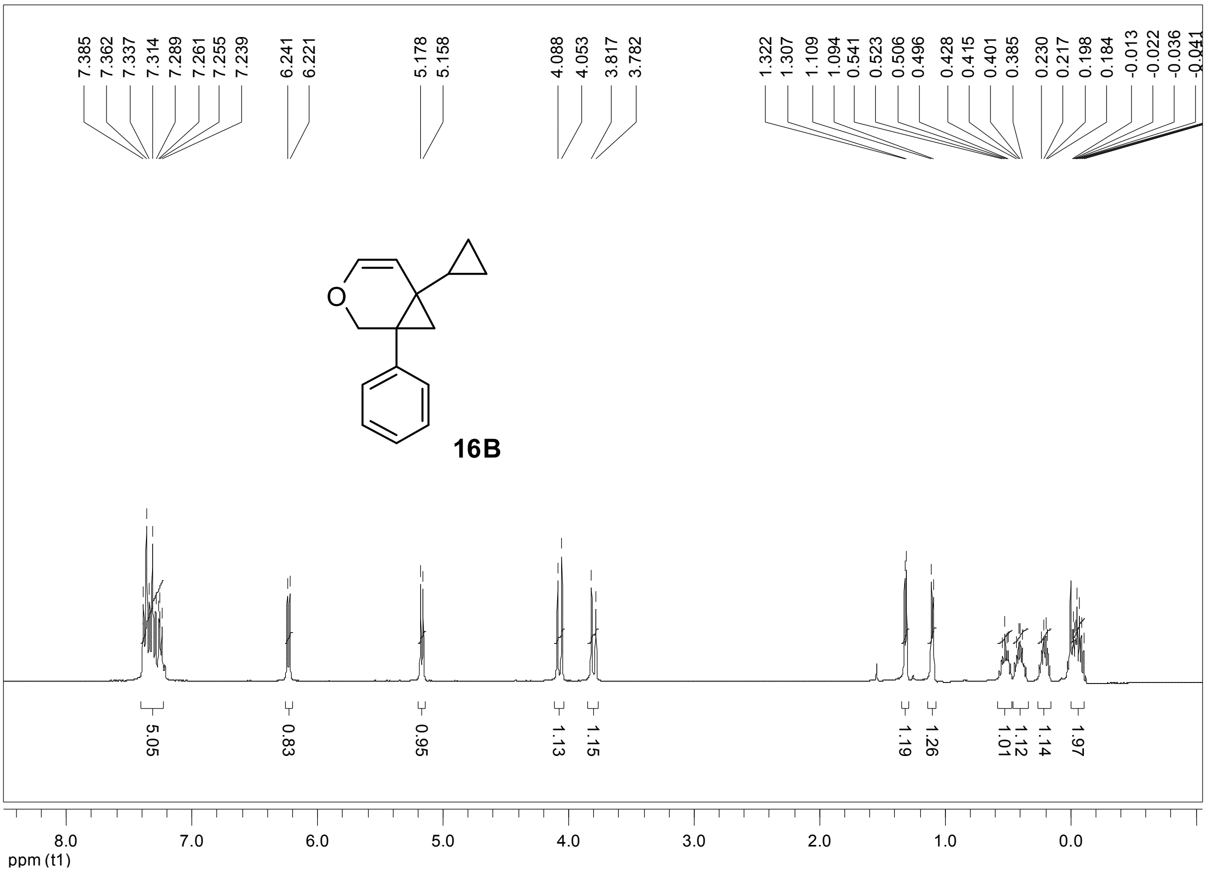
7.385
7.362
7.337
7.314
7.289
7.261
7.255
7.239
6.241
6.221
5.178
5.158
4.088
4.053
3.817
3.782
1.322
1.307
1.109
1.094
0.541
0.523
0.506
0.496
0.428
0.415
0.401
0.385
0.230
0.217
0.198
0.184
-0.013
-0.022
-0.036
-0.041
O
16B
5.05
0.83
0.95
1.13
1.15
1.19
1.26
1.01
1.12
1.14
1.97
8.0
7.0
6.0
5.0
4.0
3.0
2.0
1.0
0.0
ppm (t1)

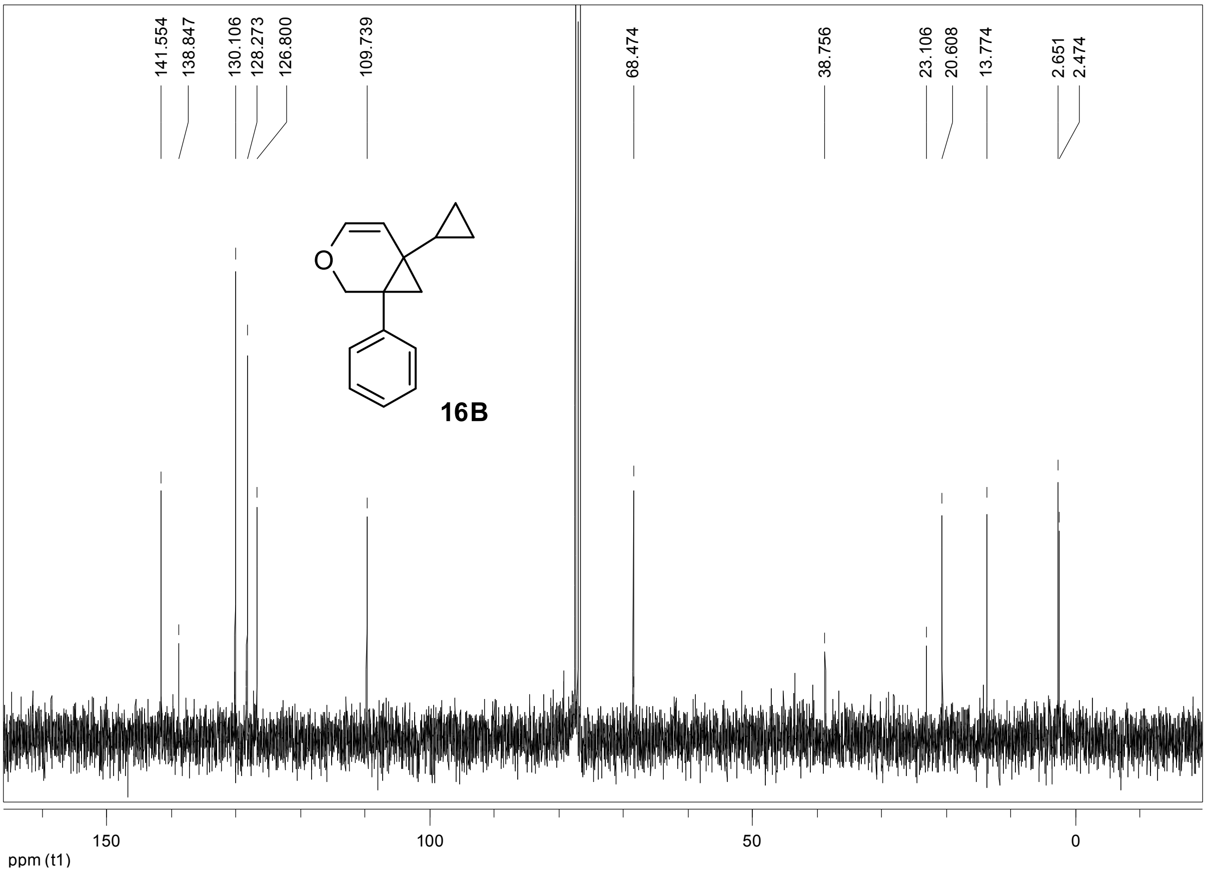
141.554
138.847
130.106
128.273
126.800
109.739
68.474
38.756
23.106
20.608
13.774
2.651
2.474
O
16B
150
100
50
0
ppm (t1)

2C

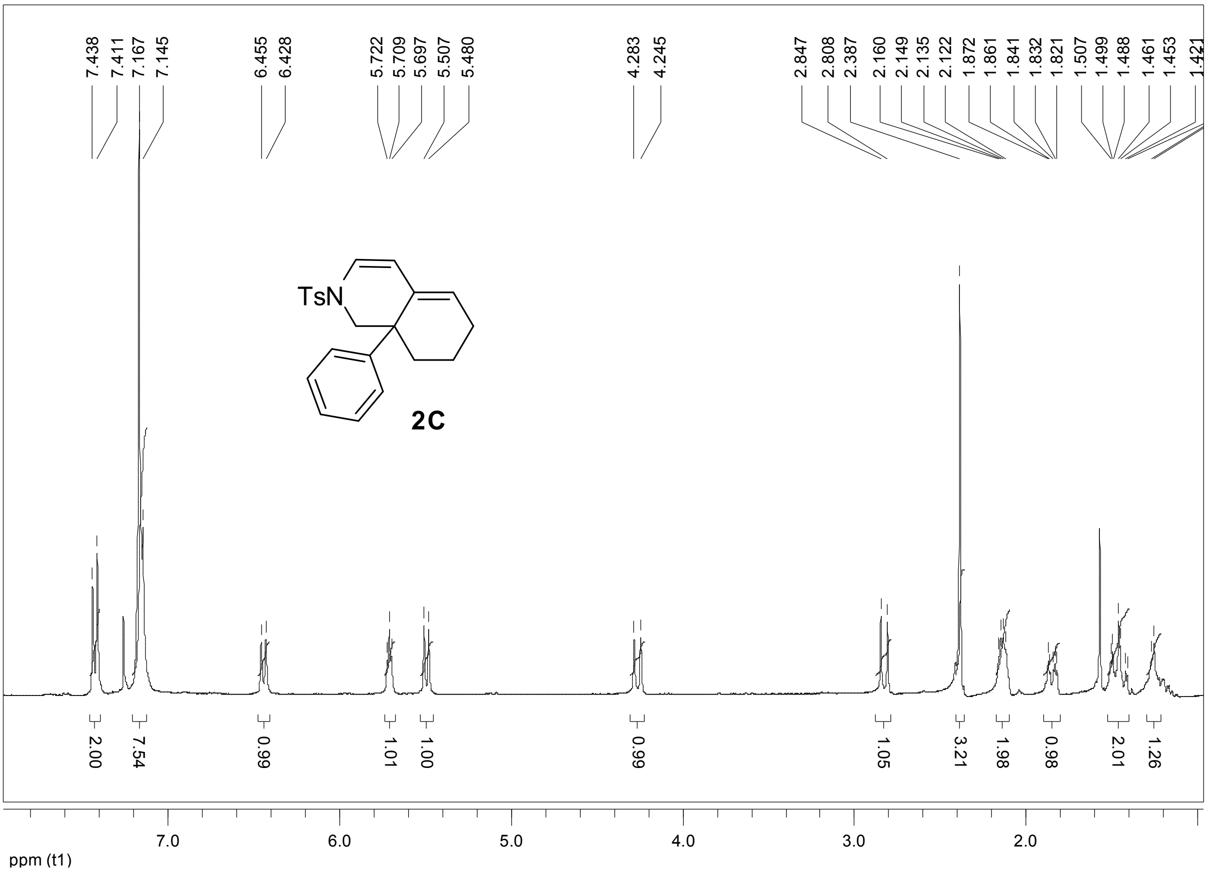
7.438
7.411
7.167
7.145
6.455
6.428
5.722
5.709
5.697
5.507
5.480
4.283
4.245
2.847
2.808
2.387
2.160
2.149
2.135
2.122
1.872
1.861
1.841
1.832
1.821
1.507
1.499
1.488
1.461
1.453
1.421
TsN
2C
2.00
7.54
0.99
1.01
1.00
0.99
1.05
3.21
1.98
0.98
2.01
1.26
7.0
6.0
5.0
4.0
3.0
2.0
ppm (t1)

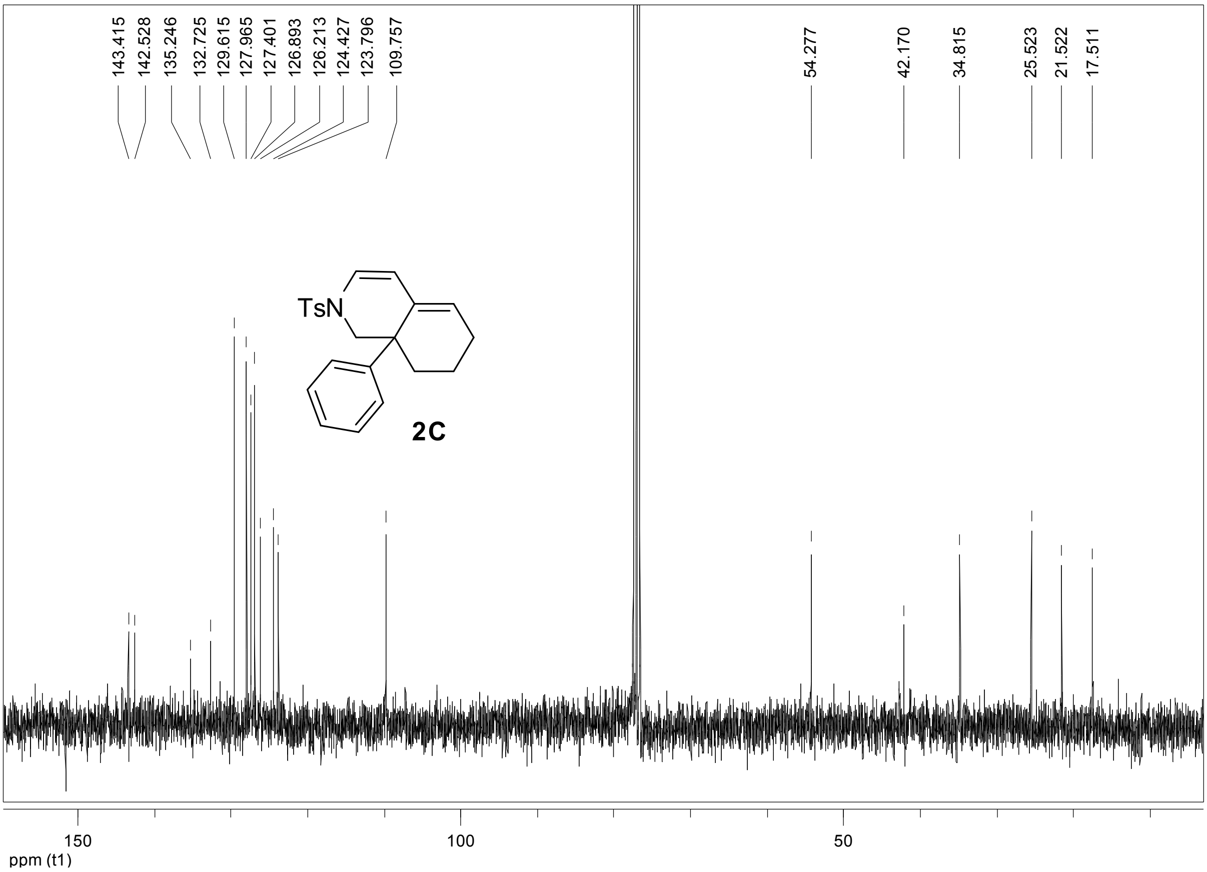
143.415
142.528
135.246
132.725
129.615
127.965
127.401
126.893
126.213
124.427
123.796
109.757
54.277
42.170
34.815
25.523
21.522
17.511
TsN
2C
150
100
50
ppm (t1)

3C

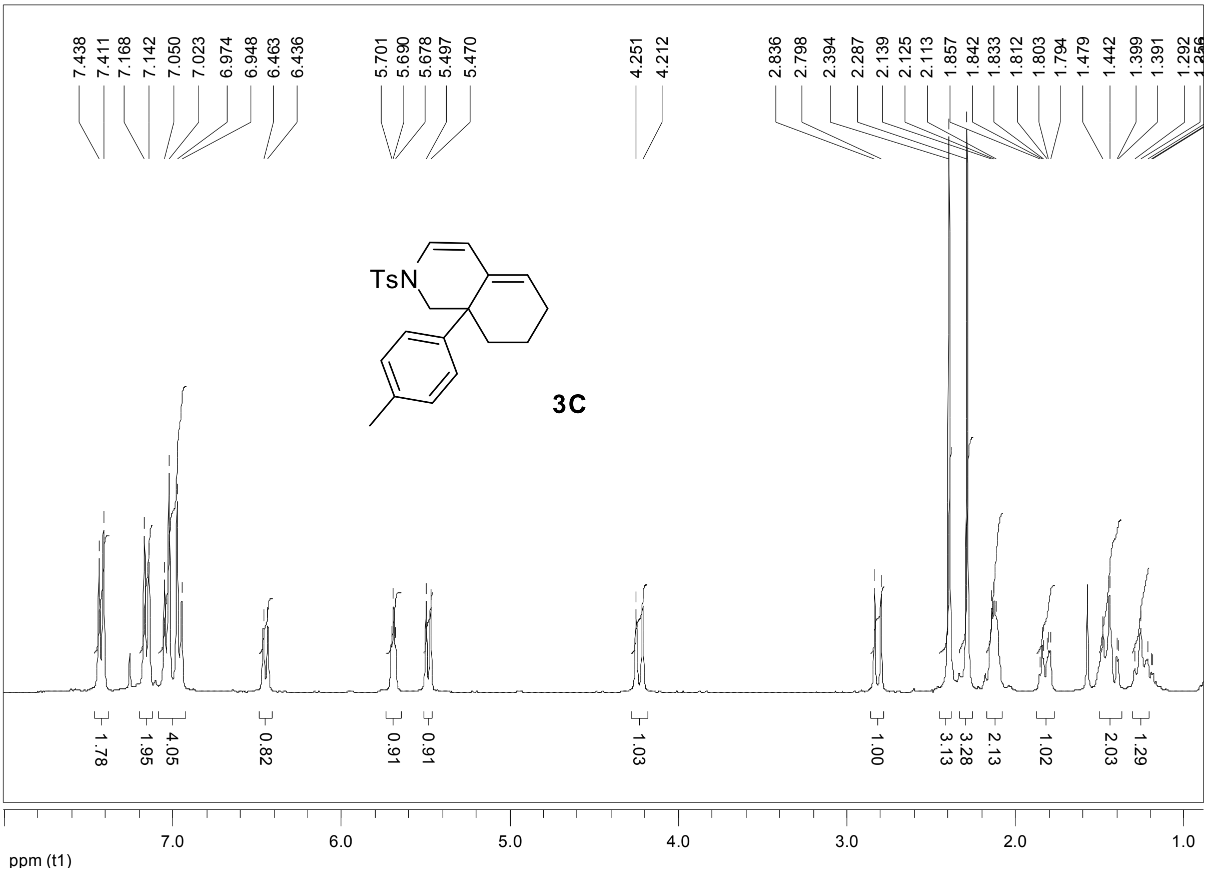
7.438
7.411
7.168
7.142
7.050
7.023
6.974
6.948
6.463
6.436
5.701
5.690
5.678
5.497
5.470
4.251
4.212
2.836
2.798
2.394
2.287
2.139
2.125
2.113
1.857
1.842
1.833
1.812
1.803
1.794
1.479
1.442
1.399
1.391
1.292
TsN
3C
1.78
1.95
4.05
0.82
0.91
0.91
1.03
1.00
3.13
3.28
2.13
1.02
2.03
1.29
7.0
6.0
5.0
4.0
3.0
2.0
1.0
ppm (t1)

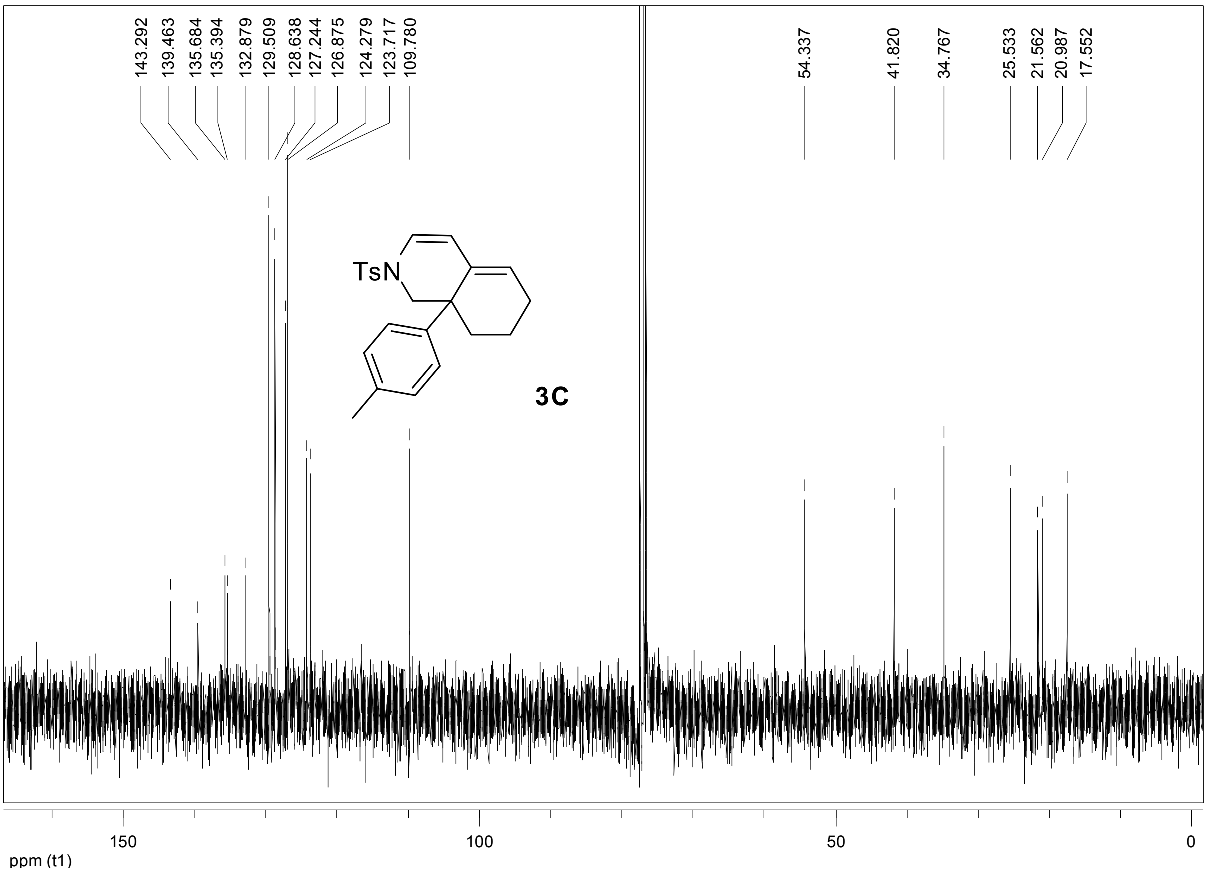
143.292
139.463
135.684
135.394
132.879
129.509
128.638
127.244
126.875
124.279
123.717
109.780
54.337
41.820
34.767
25.533
21.562
20.987
17.552
TsN
3C
150
100
50
0
ppm (t1)

4C

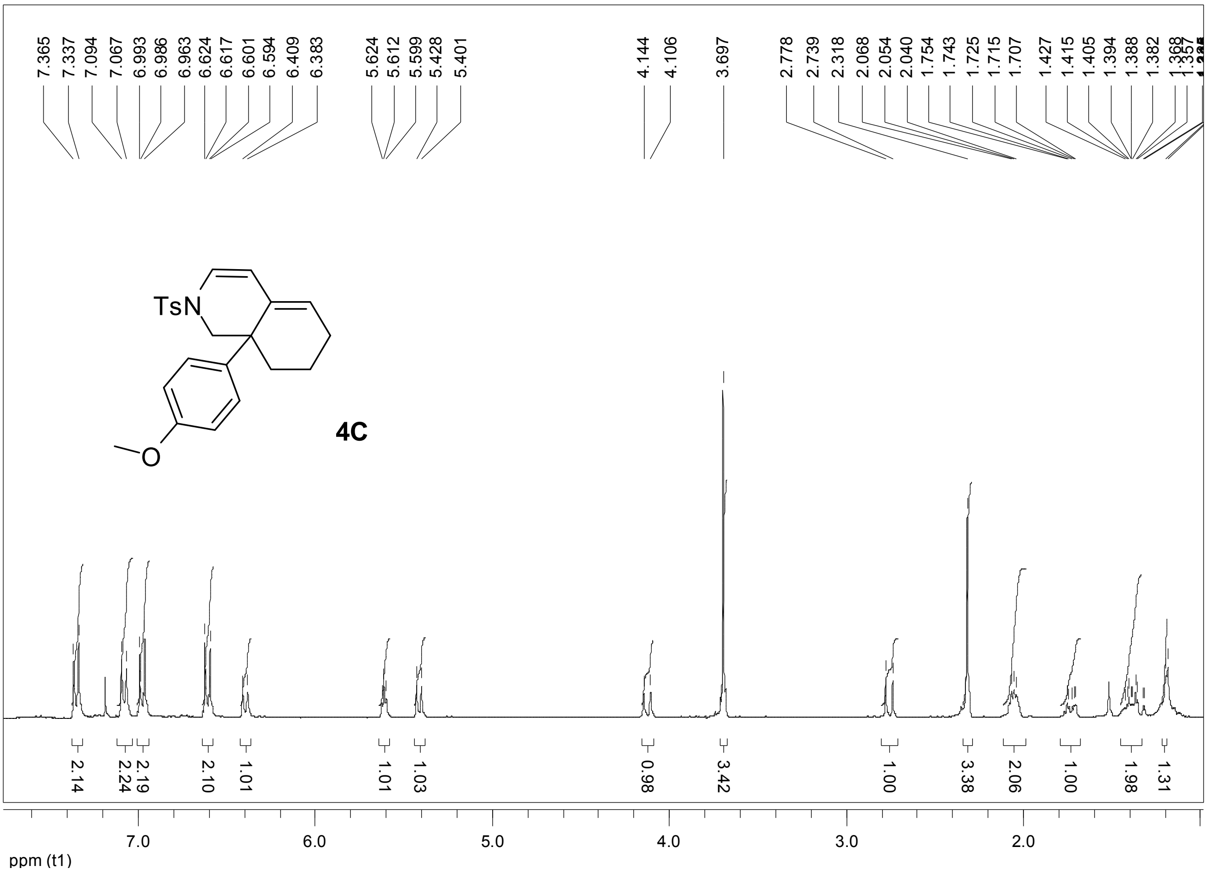
7.365
7.337
7.094
7.067
6.993
6.986
6.963
6.624
6.617
6.601
6.594
6.409
6.383
5.624
5.612
5.599
5.428
5.401
4.144
4.106
3.697
2.778
2.739
2.318
2.068
2.054
2.040
1.754
1.743
1.725
1.715
1.707
1.427
1.415
1.405
1.394
1.388
1.382
1.368
1.357
TsN
O
4C
2.14
2.24
2.19
2.10
1.01
1.01
1.03
0.98
3.42
1.00
3.38
2.06
1.00
1.98
1.31
7.0
6.0
5.0
4.0
3.0
2.0
ppm (t1)

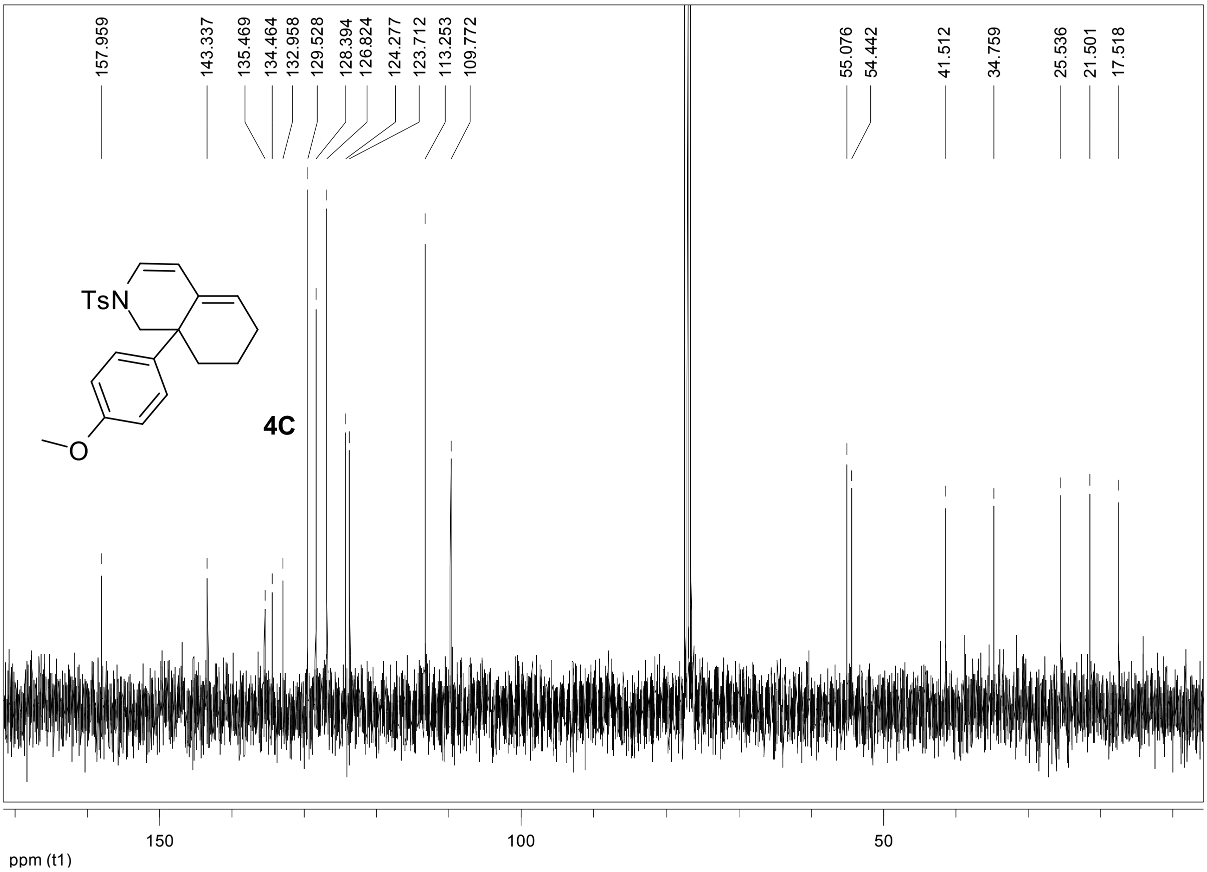
157.959
143.337
135.469
134.464
132.958
129.528
128.394
126.824
124.277
123.712
113.253
109.772
55.076
54.442
41.512
34.759
25.536
21.501
17.518
TsN
O
4C
150
100
50
ppm (t1)

5C

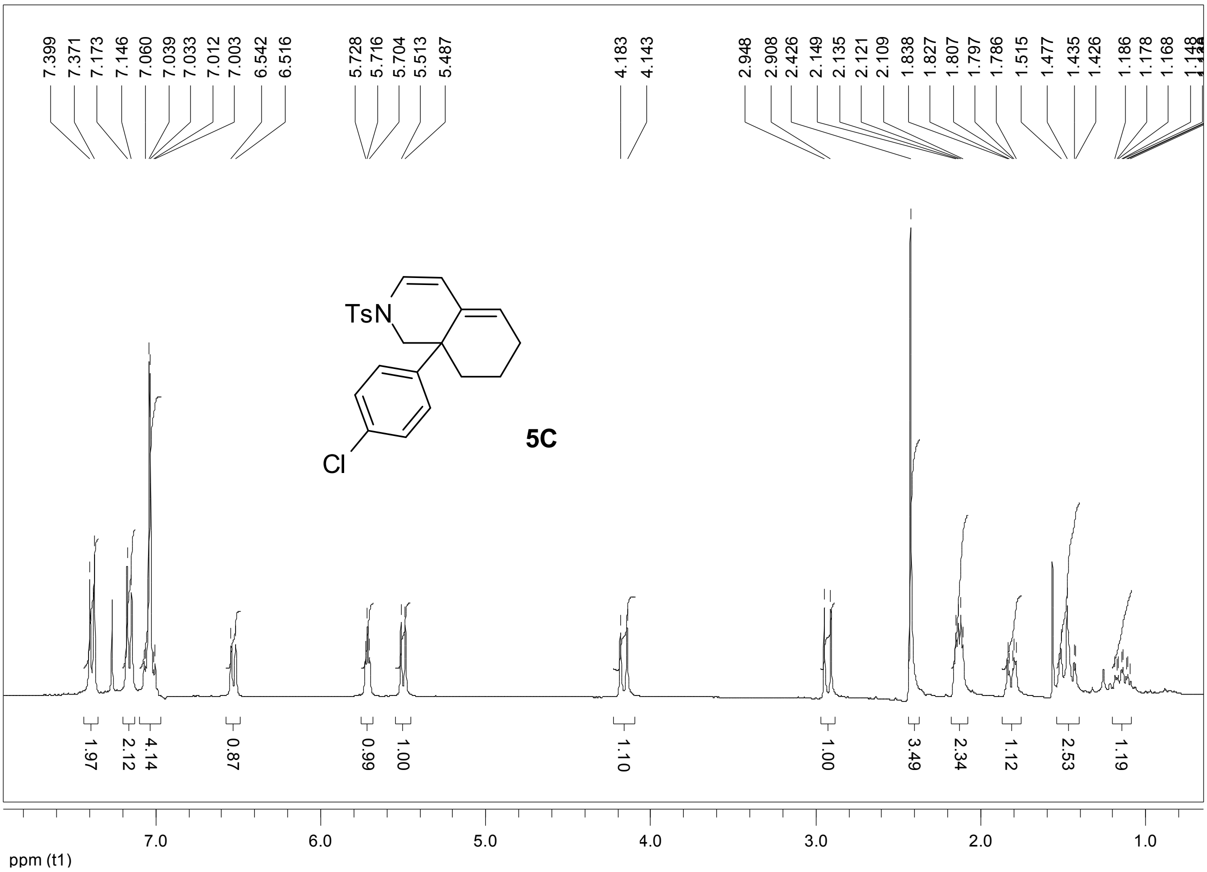
7.399
7.371
7.173
7.146
7.060
7.039
7.033
7.012
7.003
6.542
6.516
5.728
5.716
5.704
5.513
5.487
4.183
4.143
2.948
2.908
2.426
2.149
2.135
2.121
2.109
1.838
1.827
1.807
1.797
1.786
1.515
1.477
1.435
1.426
1.186
1.178
1.168
1.148
TsN
Cl
5C
1.97
2.12
4.14
0.87
0.99
1.00
1.10
1.00
3.49
2.34
1.12
2.53
1.19
7.0
6.0
5.0
4.0
3.0
2.0
1.0
ppm (t1)

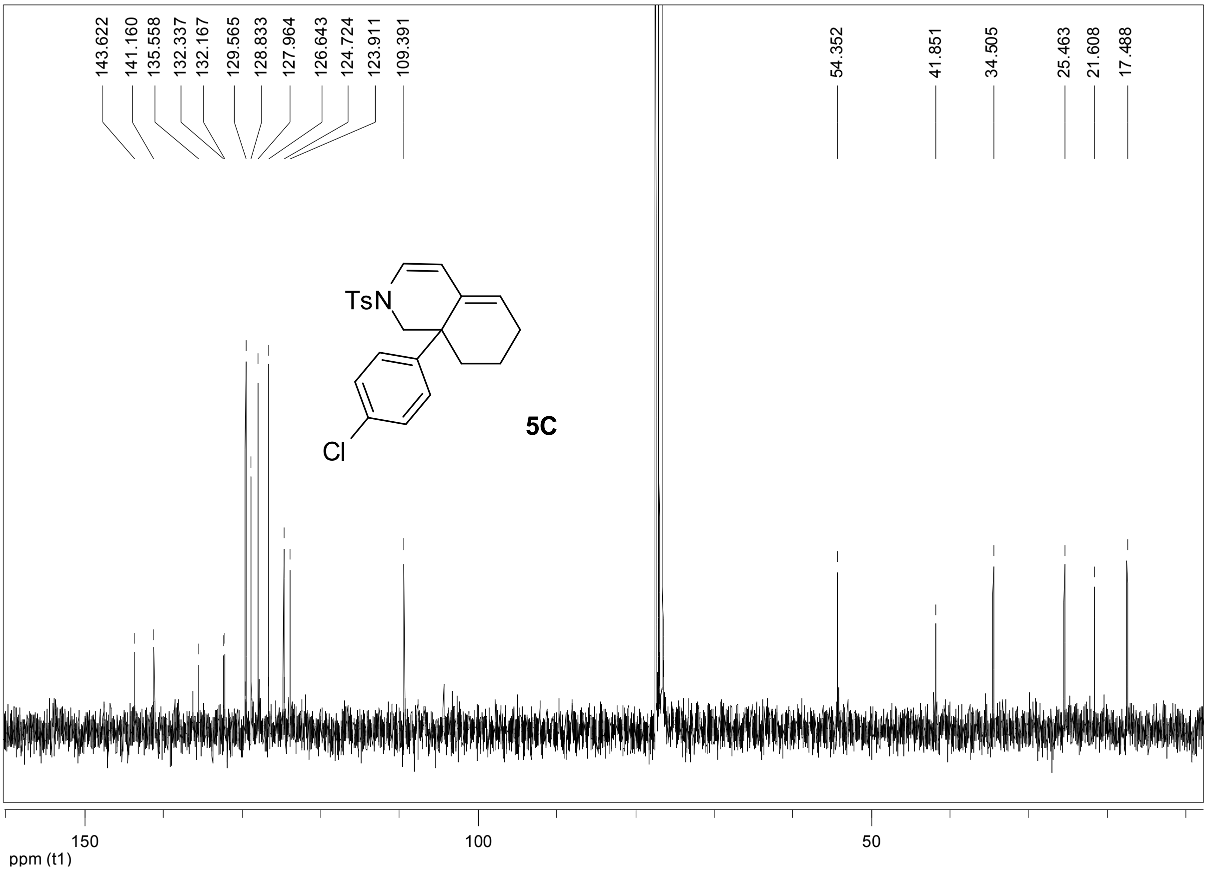
143.622
141.160
135.558
132.337
132.167
129.565
128.833
127.964
126.643
124.724
123.911
109.391
54.352
41.851
34.505
25.463
21.608
17.488
TsN
Cl
5C
150
100
50
ppm (t1)

6C

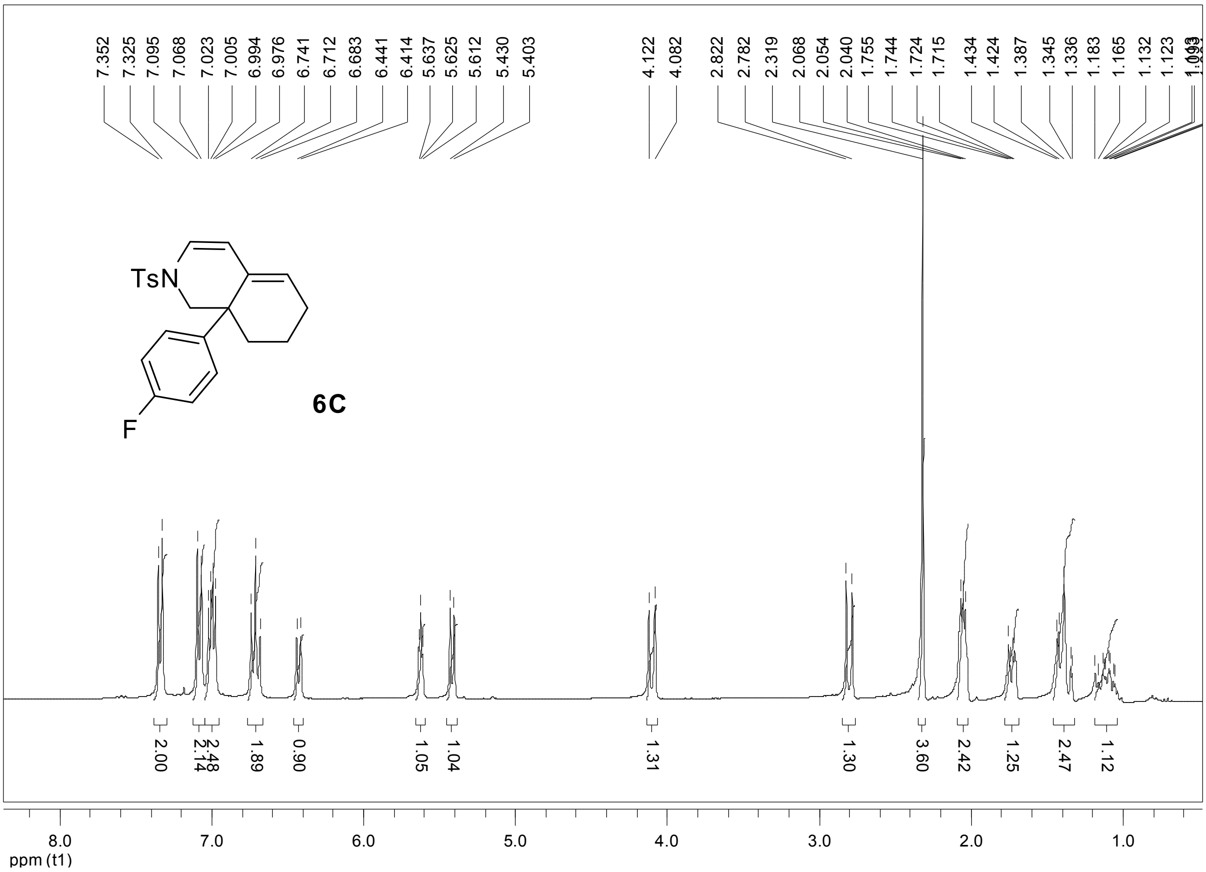

7.352
7.325
7.095
7.068
7.023
7.005
6.994
6.976
6.741
6.712
6.683
6.441
6.414
5.637
5.625
5.612
5.430
5.403
4.122
4.082
2.822
2.782
2.319
2.068
2.054
2.040
1.755
1.744
1.724
1.715
1.434
1.424
1.387
1.345
1.336
1.183
1.165
1.132
1.123
TsN
F
6C
2.00
2.14
2.48
1.89
0.90
1.05
1.04
1.31
1.30
3.60
2.42
1.25
2.47
1.12
8.0
7.0
6.0
5.0
4.0
3.0
2.0
1.0
ppm (t1)

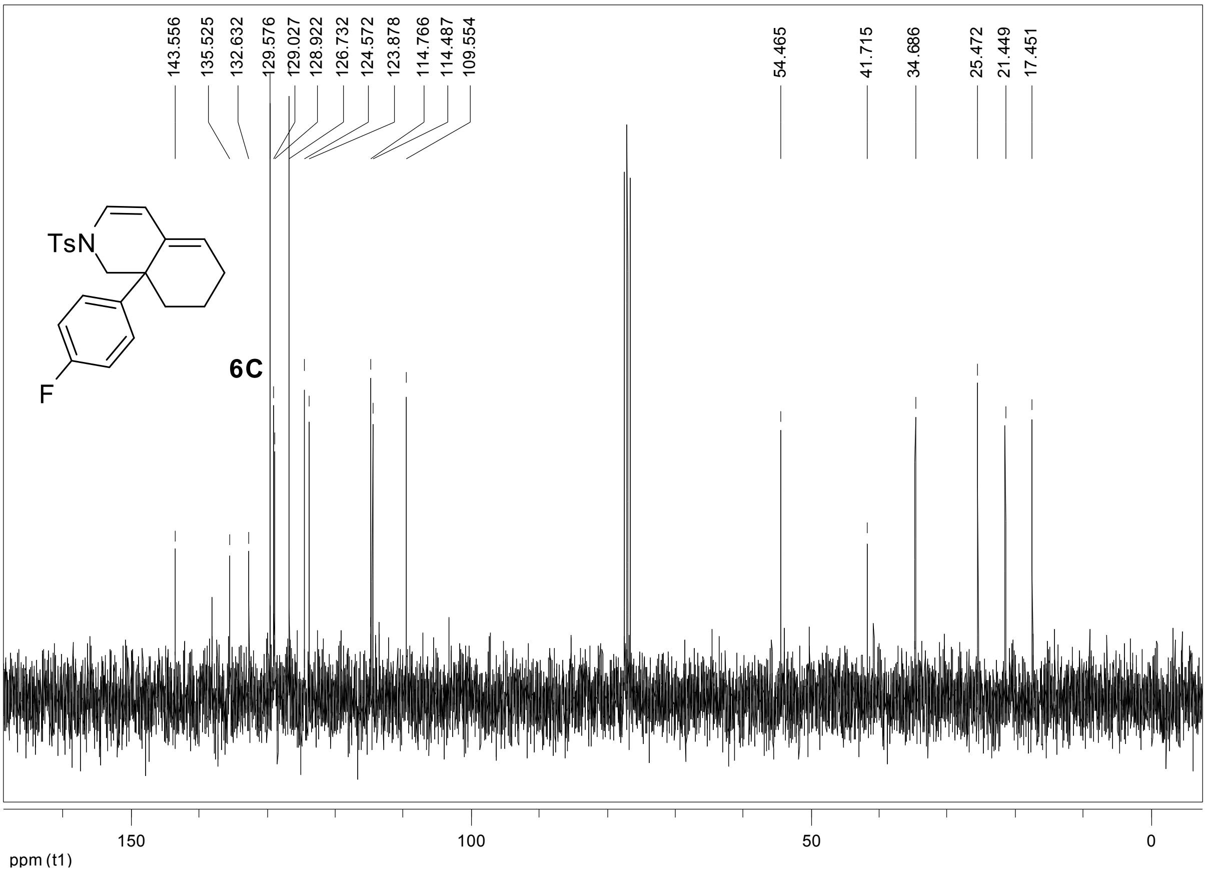

143.556
135.525
132.632
129.576
129.027
128.922
126.732
124.572
123.878
114.766
114.487
109.554
54.465
41.715
34.686
25.472
21.449
17.451
TsN
F
6C
150
100
50
0
ppm (t1)

7C

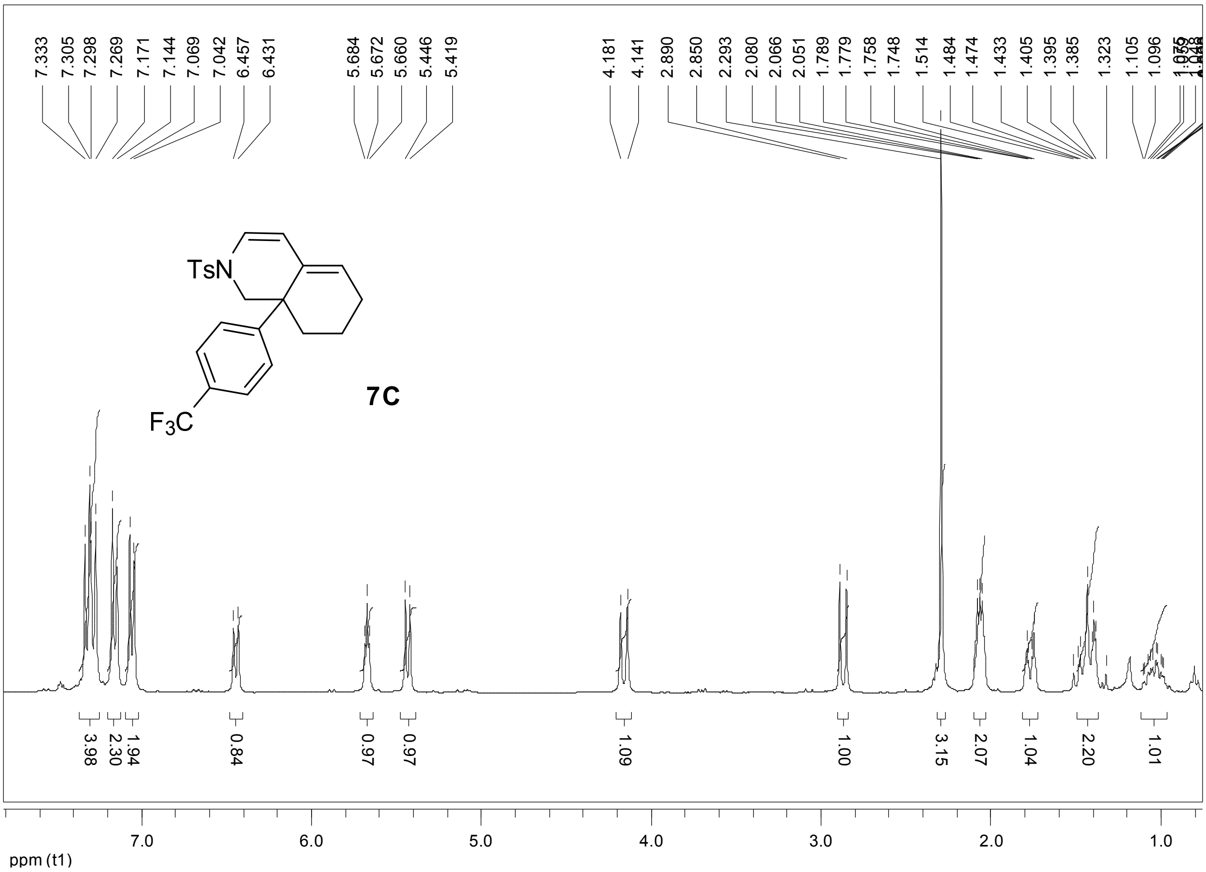
7.333
7.305
7.298
7.269
7.171
7.144
7.069
7.042
6.457
6.431
5.684
5.672
5.660
5.446
5.419
4.181
4.141
2.890
2.850
2.293
2.080
2.066
2.051
1.789
1.779
1.758
1.748
1.514
1.484
1.474
1.433
1.405
1.395
1.385
1.323
1.105
1.096
TsN
F_3C
7C
3.98
2.30
1.94
0.84
0.97
0.97
1.09
1.00
3.15
2.07
1.04
2.20
1.01
7.0
6.0
5.0
4.0
3.0
2.0
1.0
ppm (t1)

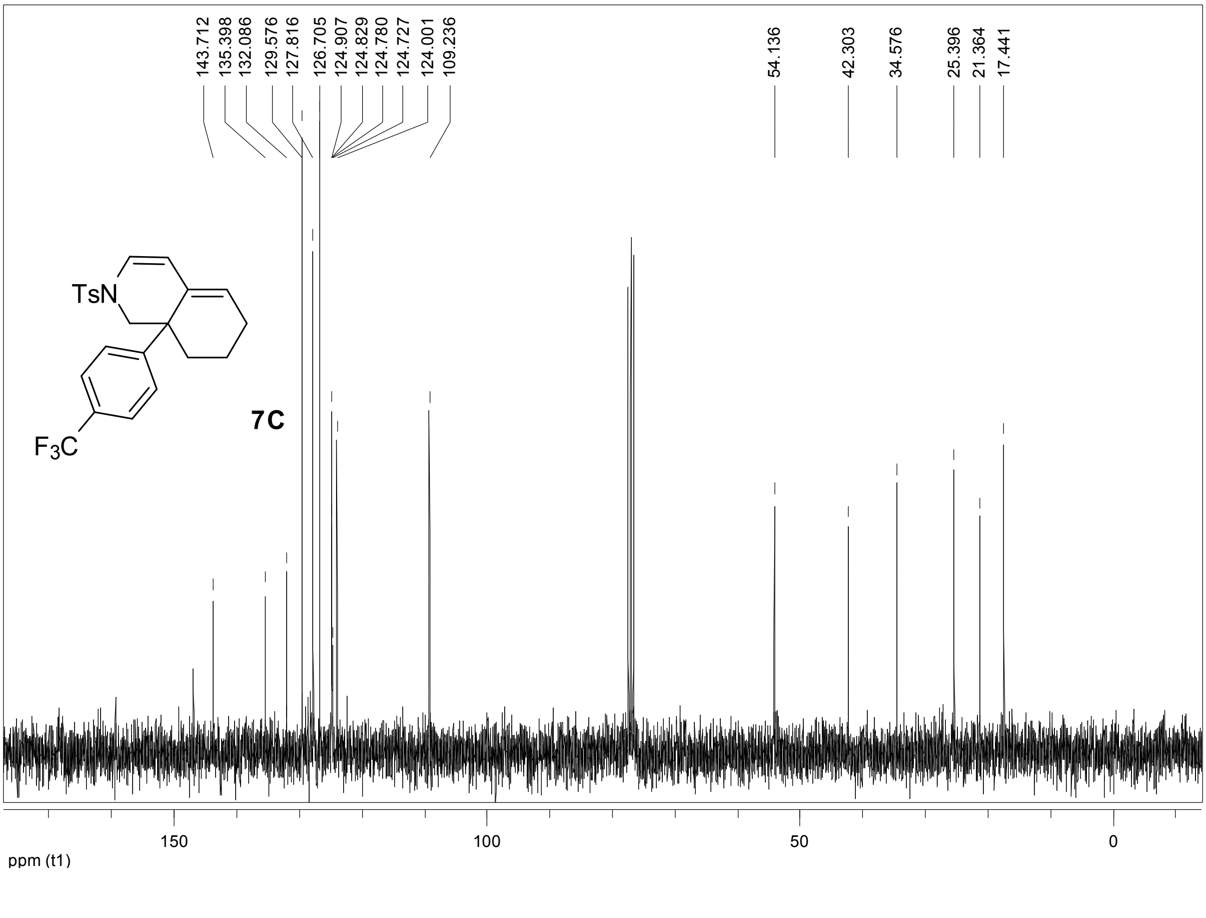
143.712
135.398
132.086
129.576
127.816
126.705
124.907
124.829
124.780
124.727
124.001
109.236
54.136
42.303
34.576
25.396
21.364
17.441
TsN
F_3C
7C
150
100
50
0
ppm (t1)

8C

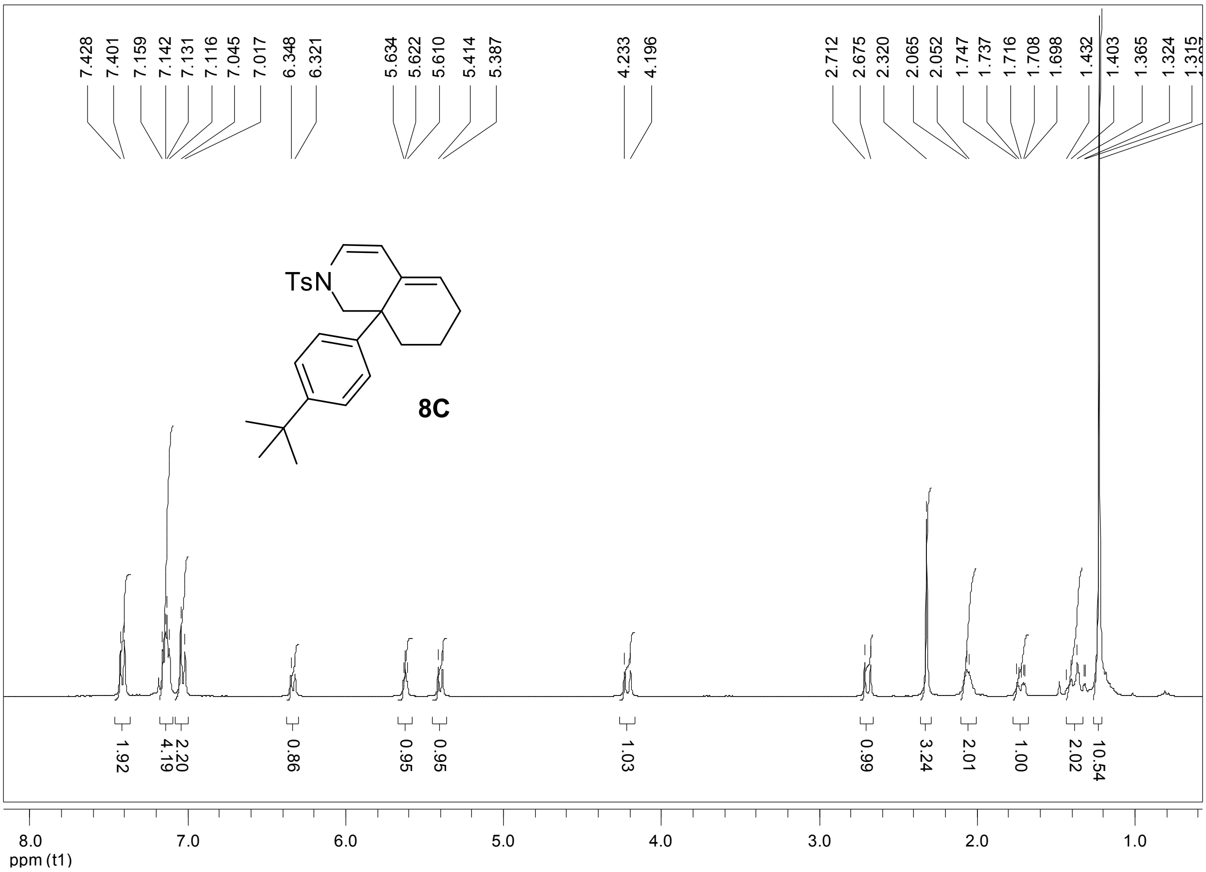
7.428
7.401
7.159
7.142
7.131
7.116
7.045
7.017
6.348
6.321
5.634
5.622
5.610
5.414
5.387
4.233
4.196
2.712
2.675
2.320
2.065
2.052
1.747
1.737
1.716
1.708
1.698
1.432
1.403
1.365
1.324
1.315
TsN
8C
1.92
4.19
2.20
0.86
0.95
0.95
1.03
0.99
3.24
2.01
1.00
2.02
10.54
8.0
7.0
6.0
5.0
4.0
3.0
2.0
1.0
ppm (t1)

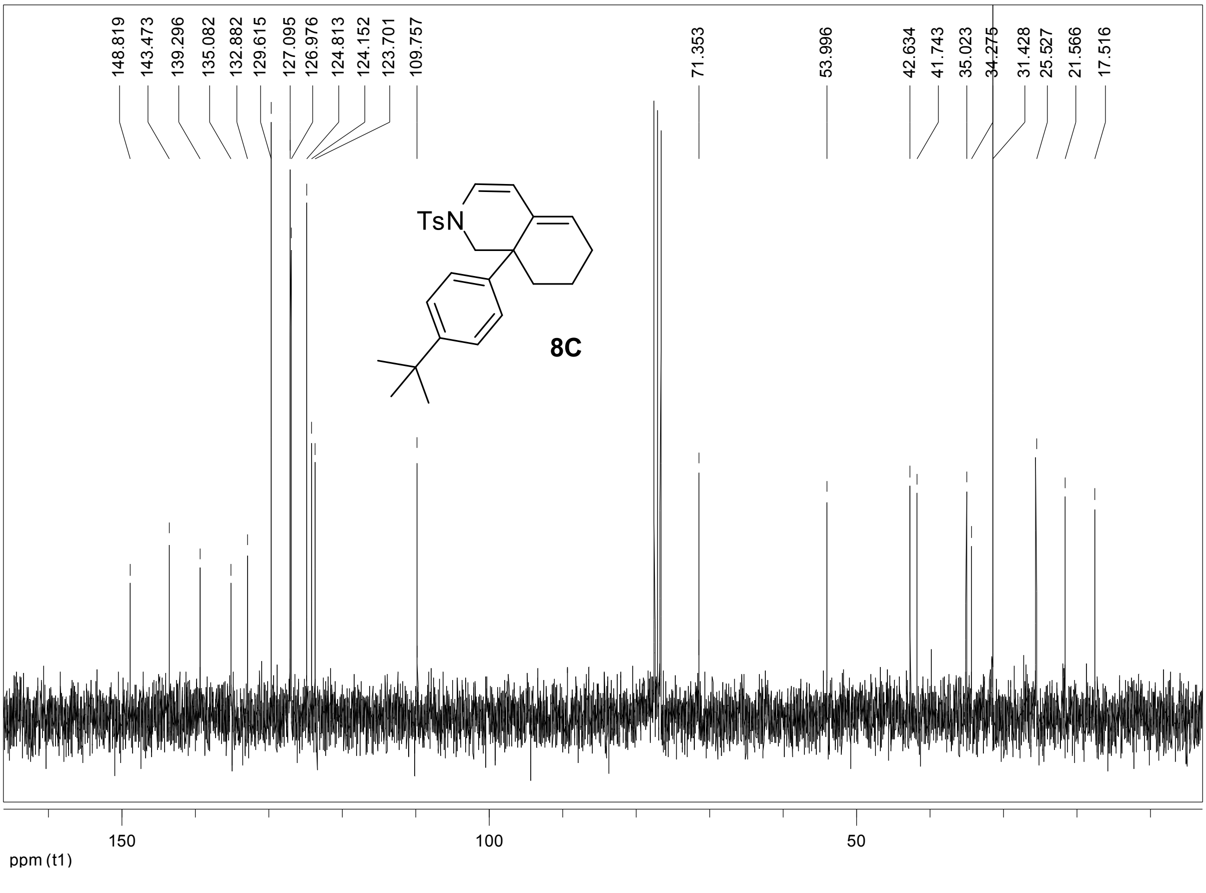
148.819
143.473
139.296
135.082
132.882
129.615
127.095
126.976
124.813
124.152
123.701
109.757
71.353
53.996
42.634
41.743
35.023
34.275
31.428
25.527
21.566
17.516
TsN
8C
150
100
50
ppm (t1)

9C

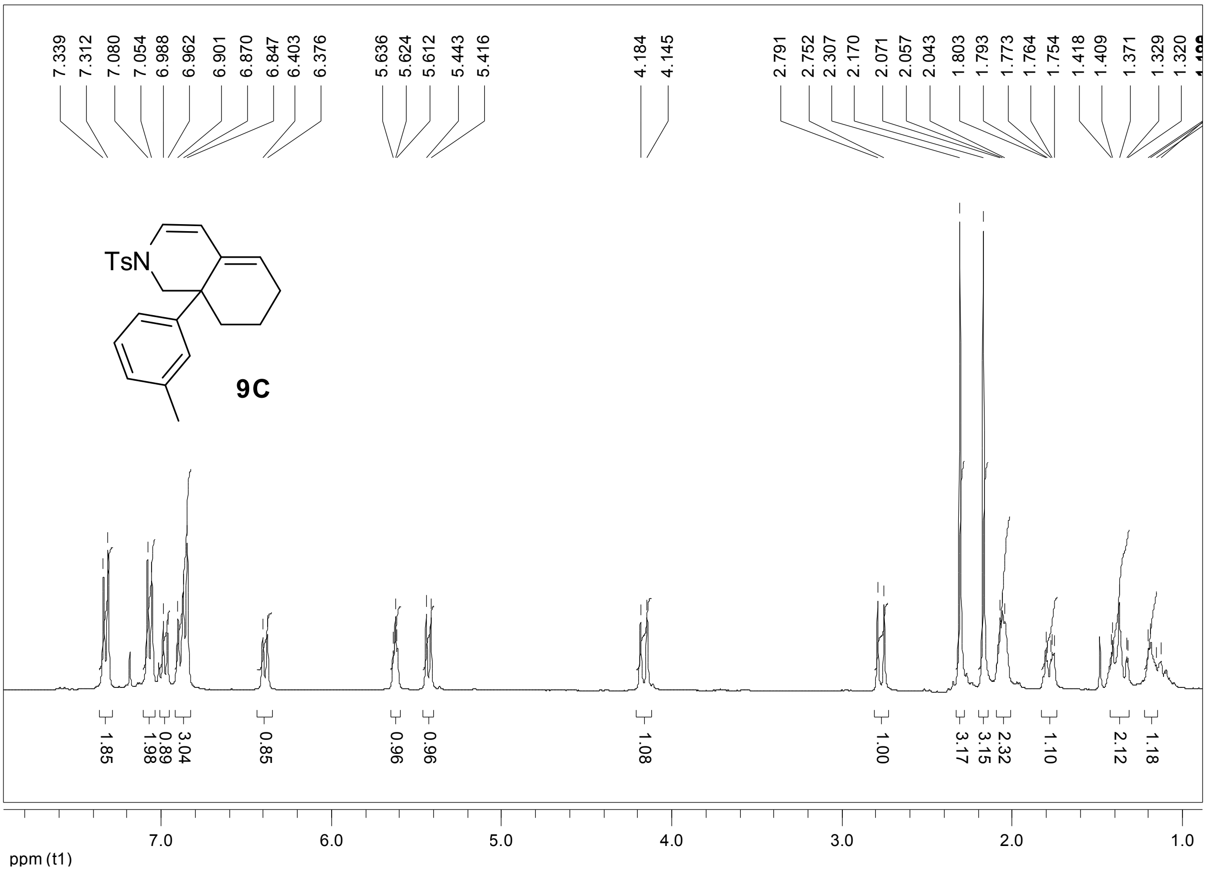
7.339
7.312
7.080
7.054
6.988
6.962
6.901
6.870
6.847
6.403
6.376
5.636
5.624
5.612
5.443
5.416
4.184
4.145
2.791
2.752
2.307
2.170
2.071
2.057
2.043
1.803
1.793
1.773
1.764
1.754
1.418
1.409
1.371
1.329
1.320
TsN
9C
1.85
1.98
0.89
3.04
0.85
0.96
0.96
1.08
1.00
3.17
3.15
2.32
1.10
2.12
1.18
7.0
6.0
5.0
4.0
3.0
2.0
1.0
ppm (t1)

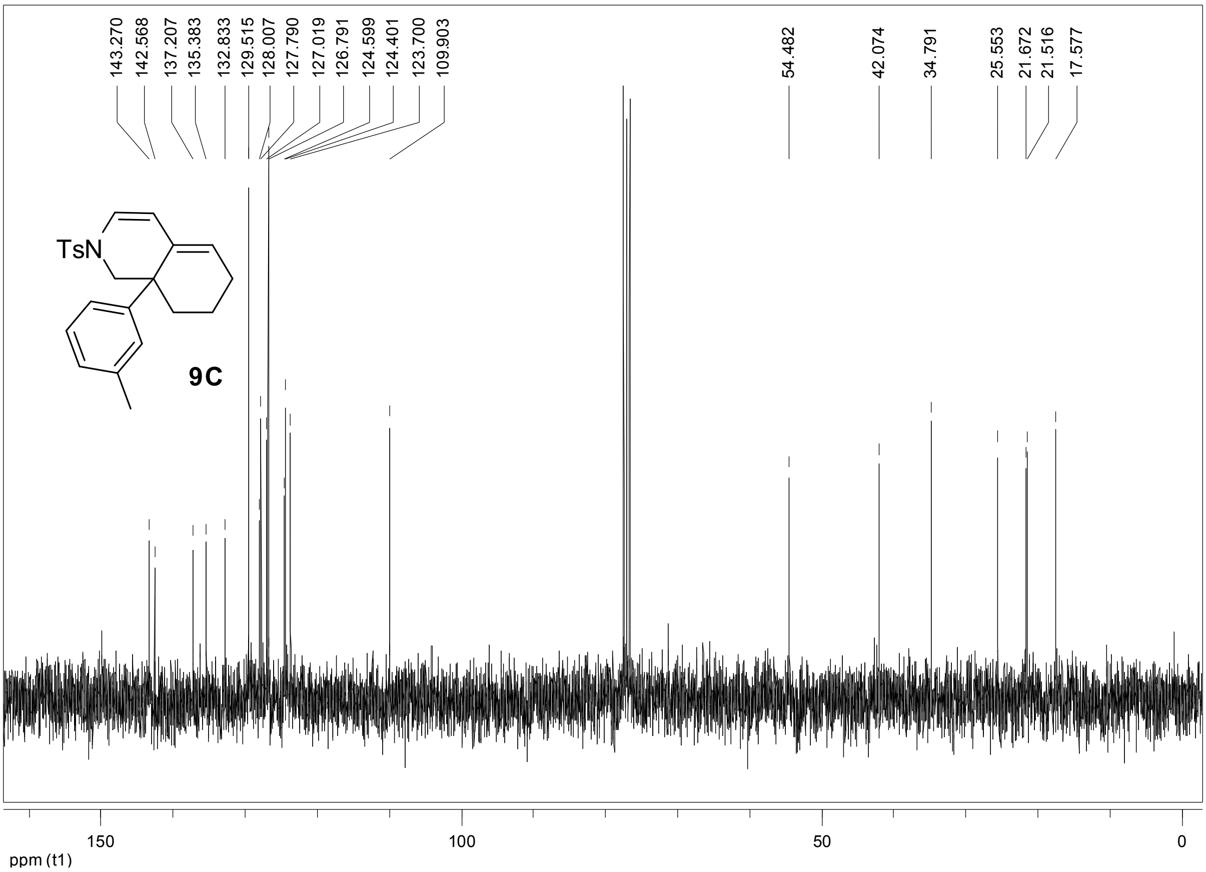
143.270
142.568
137.207
135.383
132.833
129.515
128.007
127.790
127.019
126.791
124.599
124.401
123.700
109.903
54.482
42.074
34.791
25.553
21.672
21.516
17.577
TsN
9C
150
100
50
0
ppm (t1)

10C

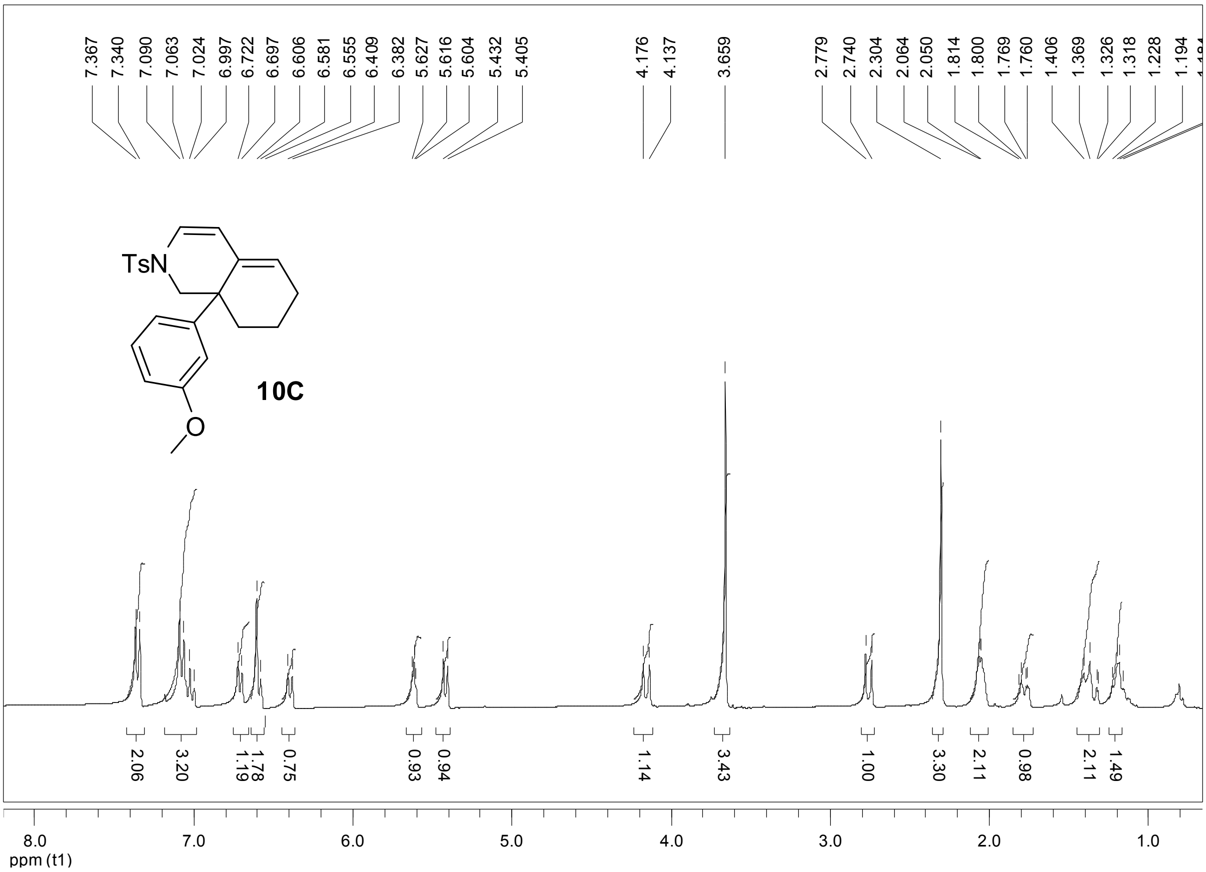
7.367
7.340
7.090
7.063
7.024
6.997
6.722
6.697
6.606
6.581
6.555
6.409
6.382
5.627
5.616
5.604
5.432
5.405
4.176
4.137
3.659
2.779
2.740
2.304
2.064
2.050
1.814
1.800
1.769
1.760
1.406
1.369
1.326
1.318
1.228
1.194
TsN
10C
O
2.06
3.20
1.19
1.78
0.75
0.93
0.94
1.14
3.43
1.00
3.30
2.11
0.98
2.11
1.49
8.0
7.0
6.0
5.0
4.0
3.0
2.0
1.0
ppm (t1)

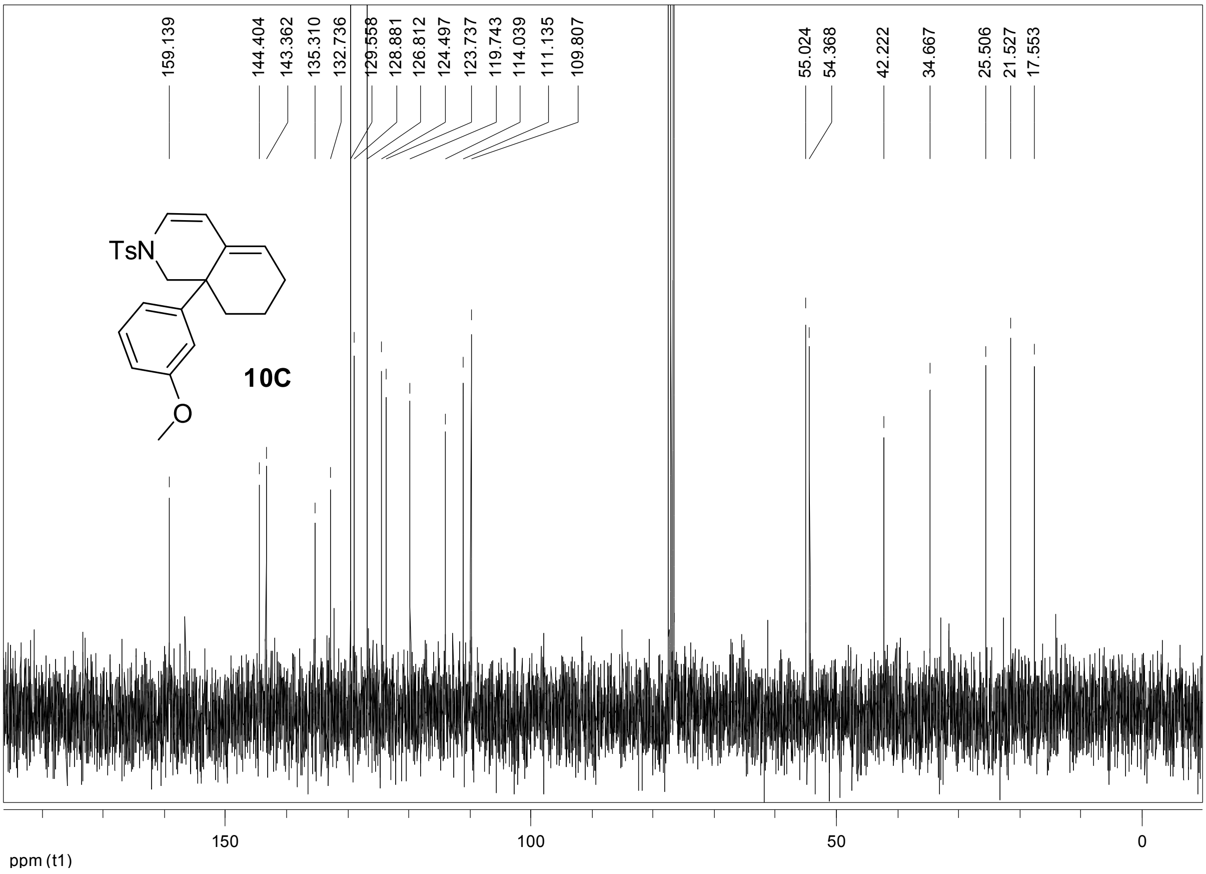
159.139
144.404
143.362
135.310
132.736
129.558
128.881
126.812
124.497
123.737
119.743
114.039
111.135
109.807
55.024
54.368
42.222
34.667
25.506
21.527
17.553
TsN
10C
O
150
100
50
0
ppm (t1)

11C

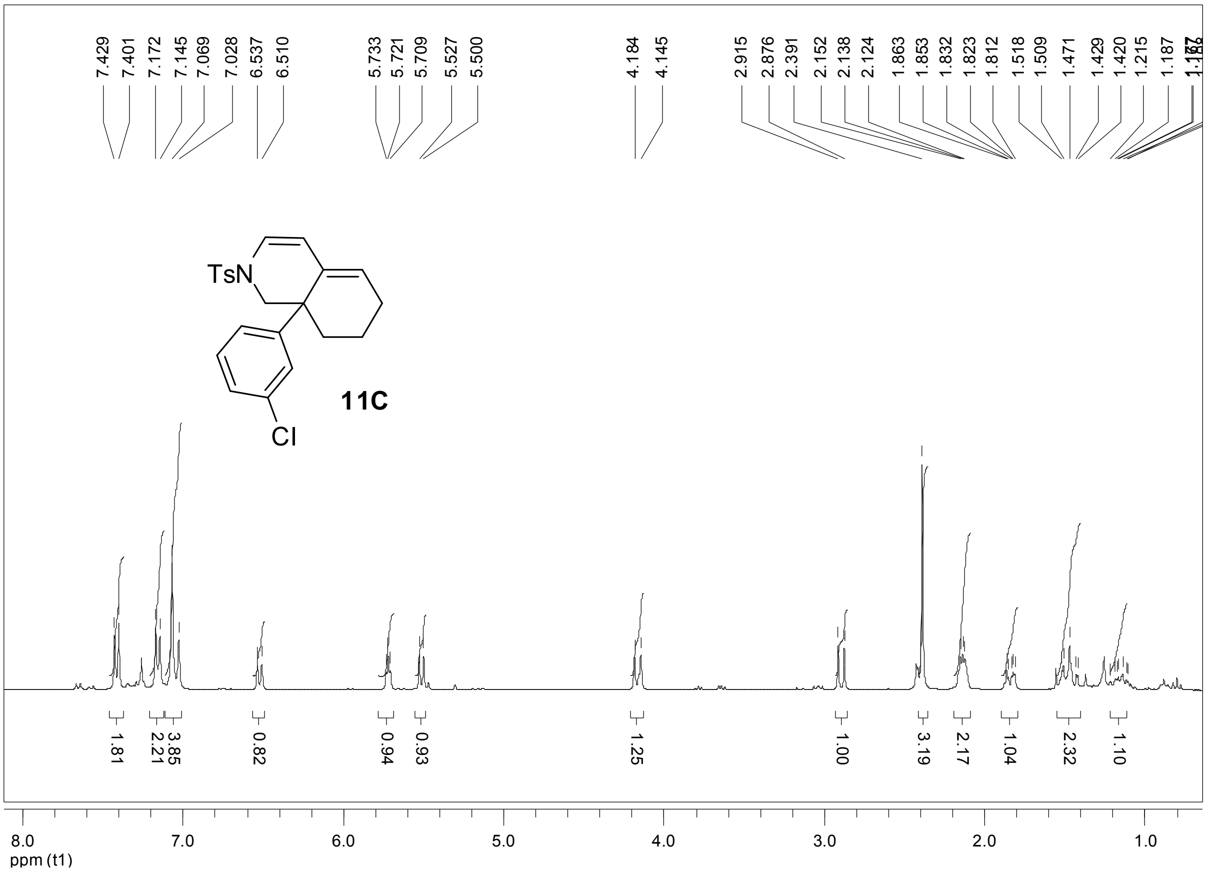
7.429
7.401
7.172
7.145
7.069
7.028
6.537
6.510
5.733
5.721
5.709
5.527
5.500
4.184
4.145
2.915
2.876
2.391
2.152
2.138
2.124
1.863
1.853
1.832
1.823
1.812
1.518
1.509
1.471
1.429
1.420
1.215
1.187
TsN
11C
Cl
1.81
2.21
3.85
0.82
0.94
0.93
1.25
1.00
3.19
2.17
1.04
2.32
1.10
8.0
7.0
6.0
5.0
4.0
3.0
2.0
1.0
ppm (t1)

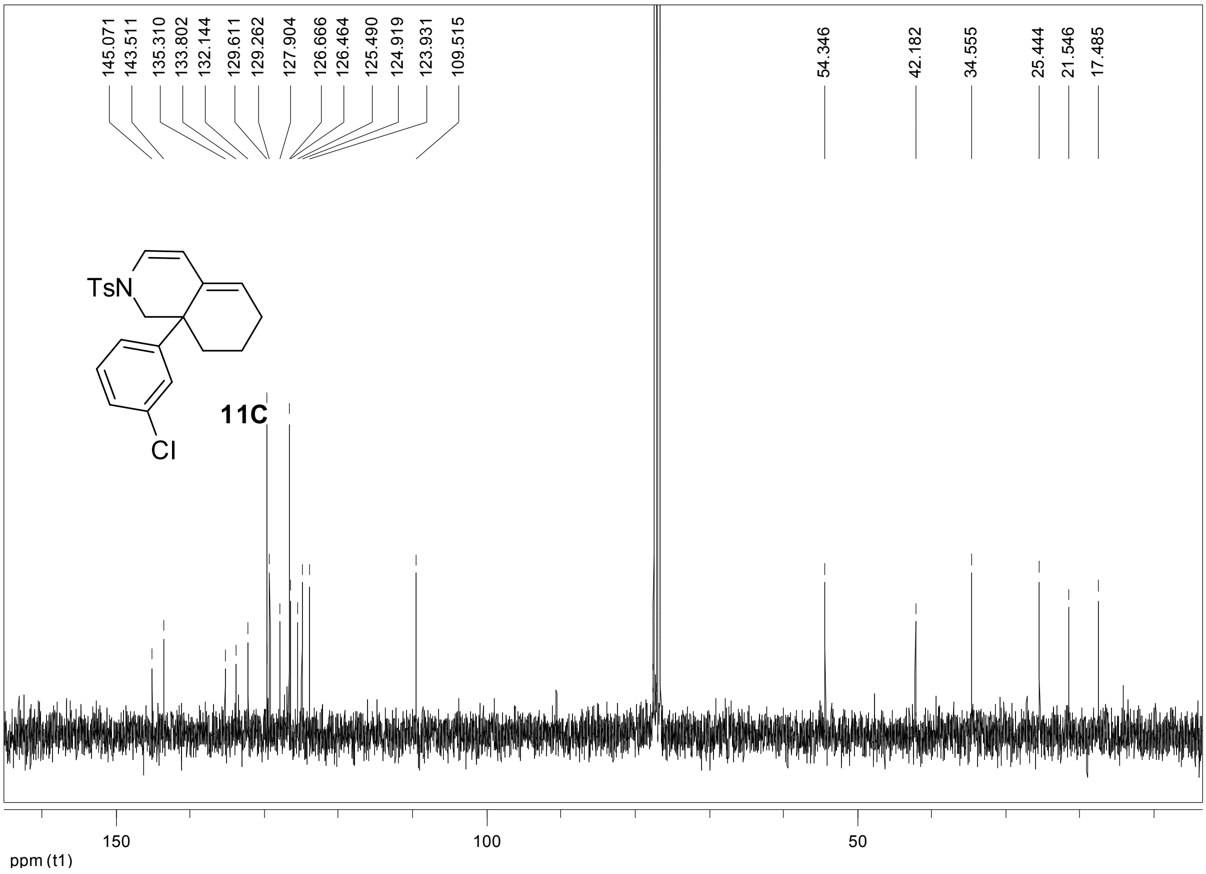
145.071
143.511
135.310
133.802
132.144
129.611
129.262
127.904
126.666
126.464
125.490
124.919
123.931
109.515
54.346
42.182
34.555
25.444
21.546
17.485
TsN
11C
Cl
150
100
50
ppm (t1)

12C

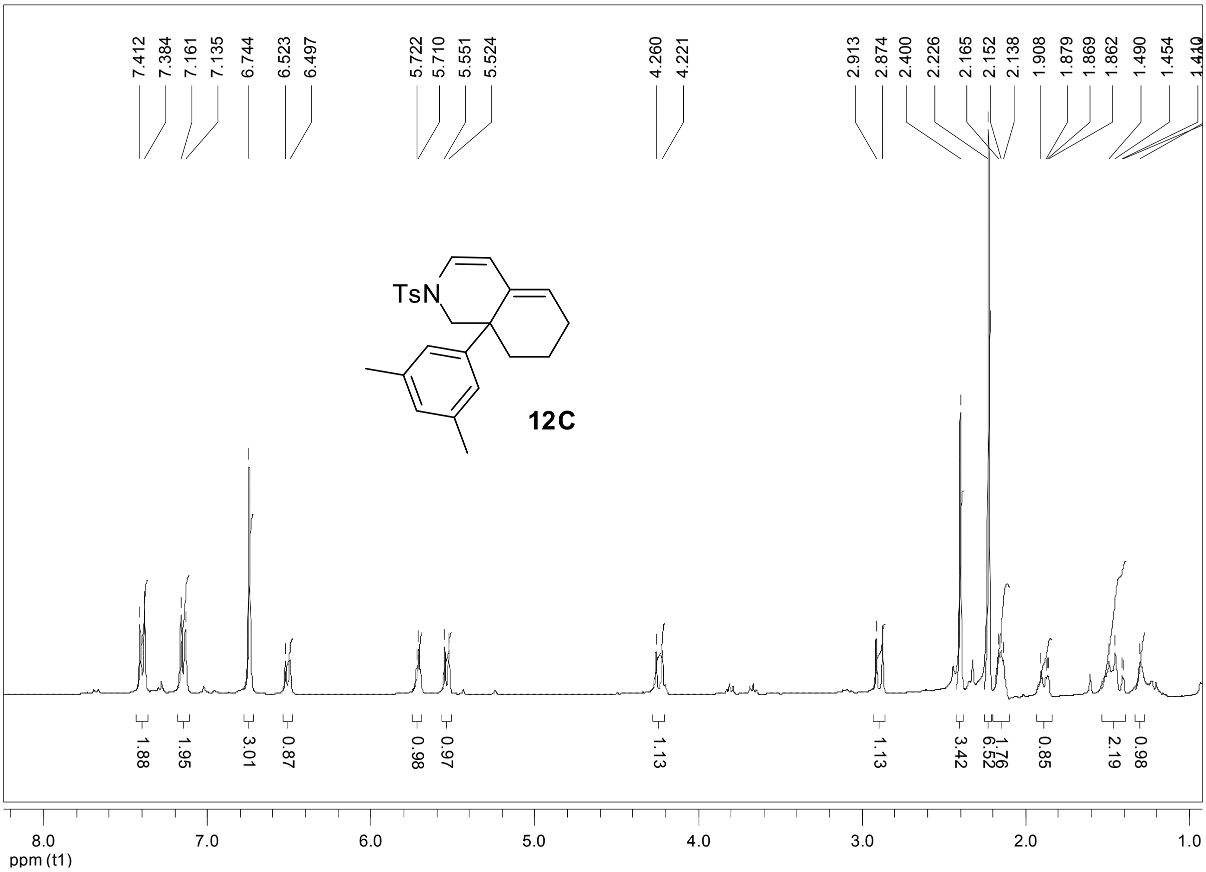
7.412
7.384
7.161
7.135
6.744
6.523
6.497
5.722
5.710
5.551
5.524
4.260
4.221
2.913
2.874
2.400
2.226
2.165
2.152
2.138
1.908
1.879
1.869
1.862
1.490
1.454
TsN
12C
1.88
1.95
3.01
0.87
0.98
0.97
1.13
1.13
3.42
6.52
1.76
0.85
2.19
0.98
8.0
7.0
6.0
5.0
4.0
3.0
2.0
1.0
ppm (t1)

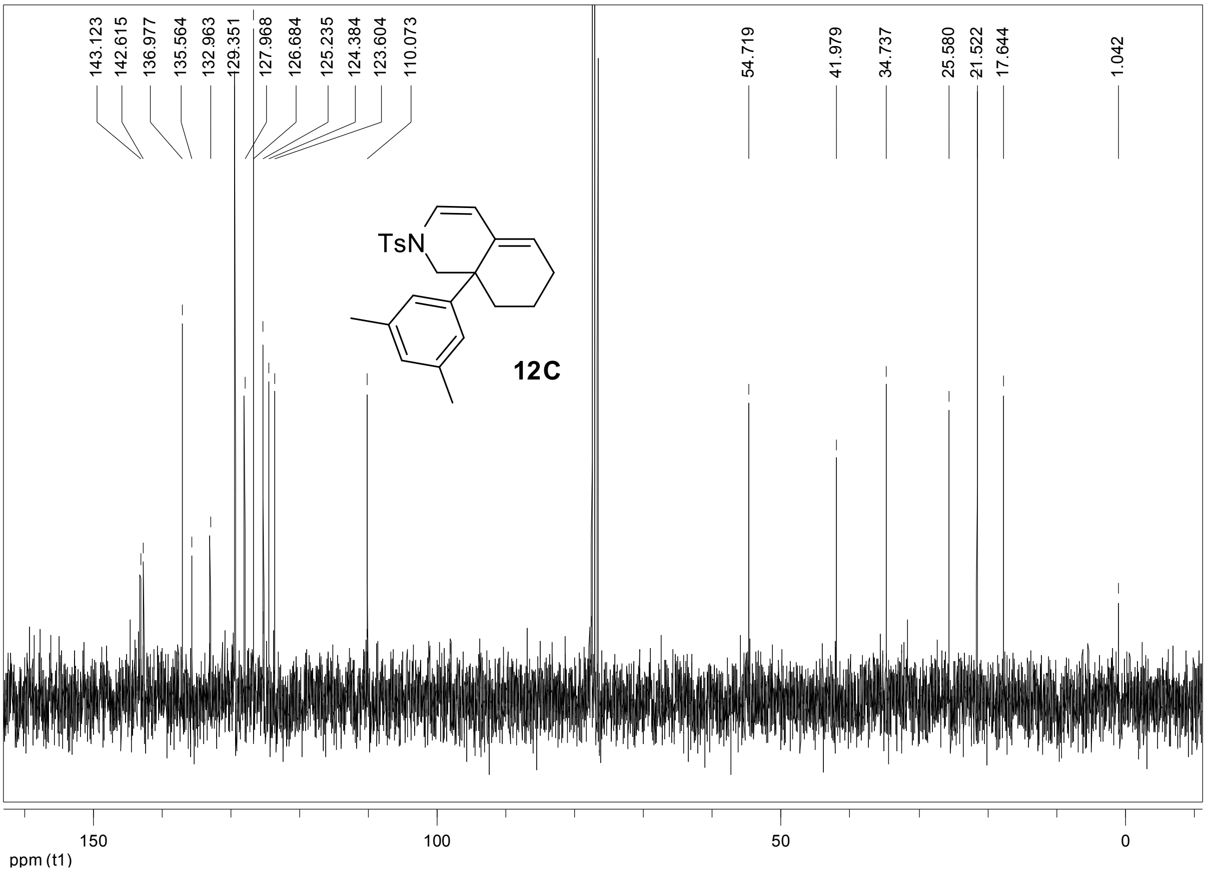
143.123
142.615
136.977
135.564
132.963
129.351
127.968
126.684
125.235
124.384
123.604
110.073
54.719
41.979
34.737
25.580
21.522
17.644
1.042
TsN
12C
150
100
50
0
ppm (t1)

15C

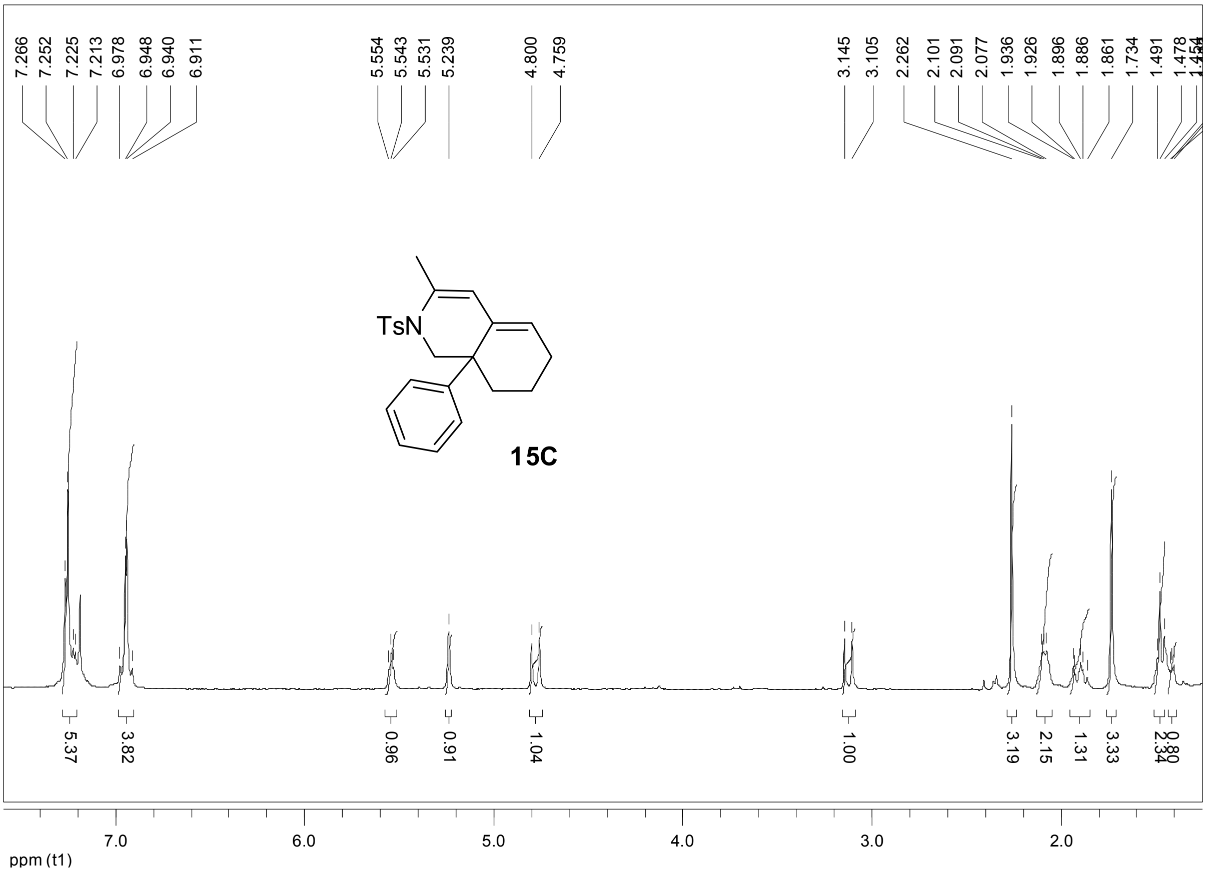
7.266
7.252
7.225
7.213
6.978
6.948
6.940
6.911
5.554
5.543
5.531
5.239
4.800
4.759
3.145
3.105
2.262
2.101
2.091
2.077
1.936
1.926
1.896
1.886
1.861
1.734
1.491
1.478
TsN
15C
5.37
3.82
0.96
0.91
1.04
1.00
3.19
2.15
1.31
3.33
2.34
0.80
7.0
6.0
5.0
4.0
3.0
2.0
ppm (t1)

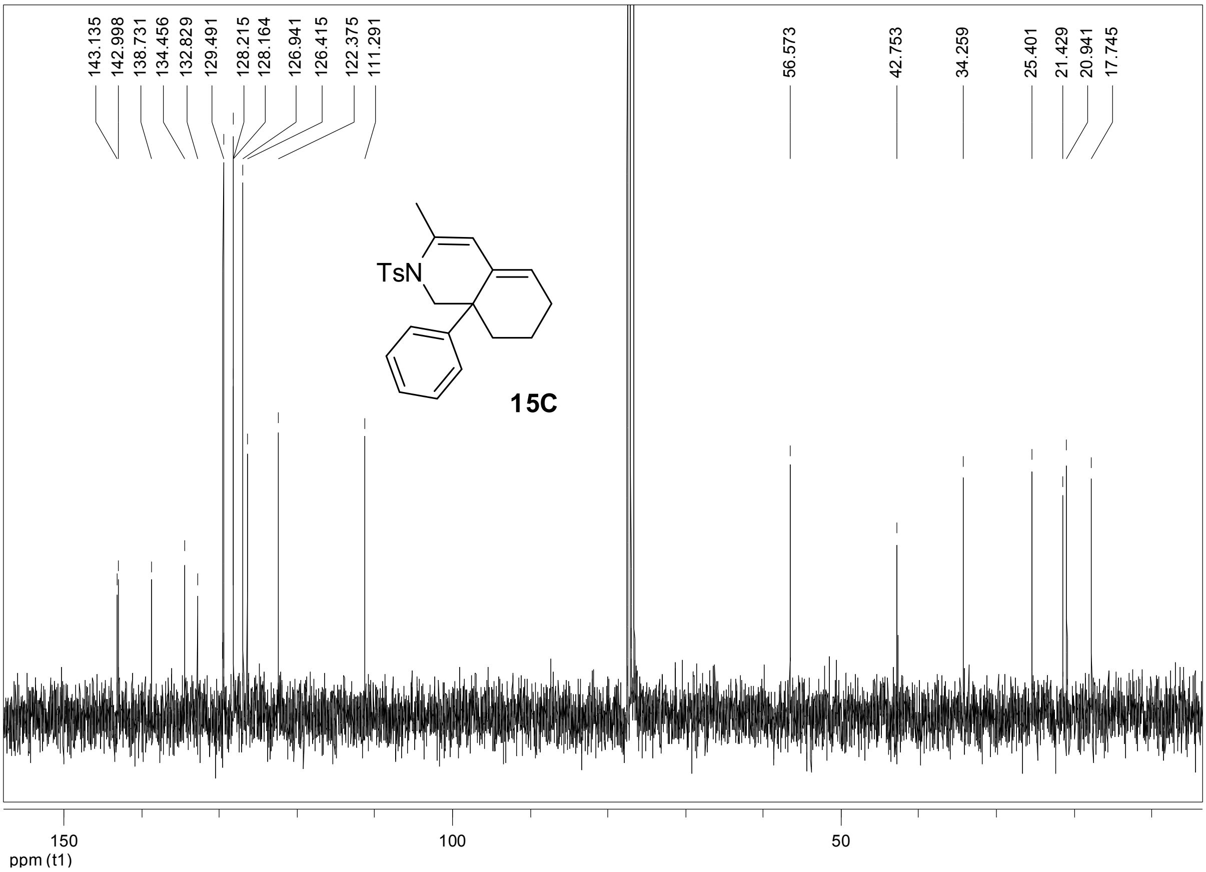
143.135
142.998
138.731
134.456
132.829
129.491
128.215
128.164
126.941
126.415
122.375
111.291
56.573
42.753
34.259
25.401
21.429
20.941
17.745
TsN
15C
150
100
50
ppm (t1)

X-ray analysis
Single crystal diffraction data were measured by a Bruker-Nonius CCD single-crystal X-ray diffractometer at room temperature by using graphite-monochromated Mo Ka radiation (l = 0.71073 Å). Preliminary orientation matrices and unit cell parameters were obtained from the peaks of the first 10 frames and then refined using the whole data set. Frames were integrated and corrected for Lorentz and polarization effects using DENZO. The structure was solved by direct methods using SHELXS-97, and refined by full-matrix least-squares with SHELXL-97. All non-hydrogen atoms were refined anisotropically and all hydrogen atoms were treated as idealized contributions. Crystal data for **2c**: $C_{22}H_{23}NO_2S$ (295K). M = 365.5, orthorhombic, space group P c a b , *a* = 13.4556(11) Å, *b* = 13.6408(10) Å, *c* = *21.0602*(12) Å, *α* = 90.000(0)°, *β* = 90.000(0)°, *γ* = 90.000(0)°, *V* = 3865.50(5) Å^3, *Z* = 8, r $_{calc.}$ = 1.25 g/cm^{-3}, absorption coefficient = 0.183 mm-1, total reflections collected 8093, unique 4407 (R_{int} = 0.059), GOF = 0.975, R_1 = 0.156, R_w = 0.125 (I >2s(I)). CCDC reference numbers CCDC 882216 contains the supplementary crystallographic data for this paper. These data can be obtained free of charge from The Cambridge Crystallographic Data Centre via www.ccdc.cam.ac.uk/data_request/cif.

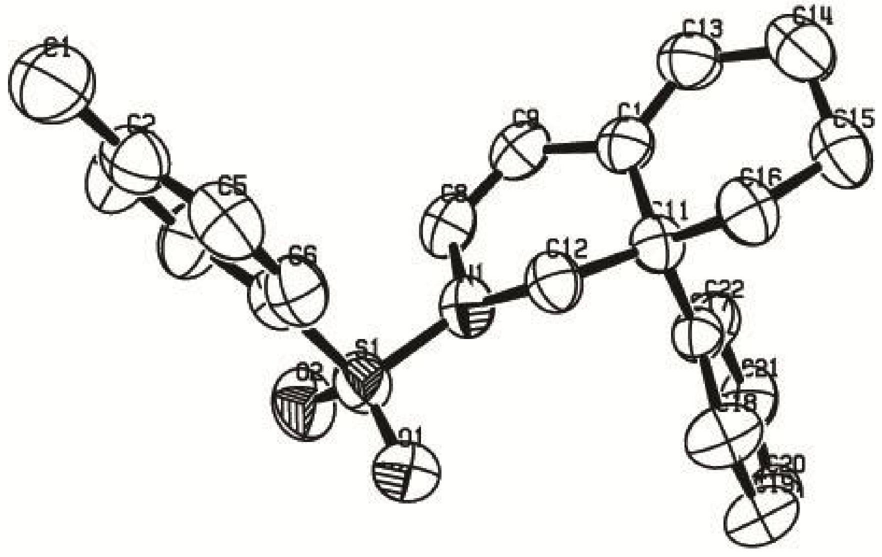

An ORTEP drawing of **2C.** (CCDC 882216)

Single crystals of **2C** suitable for an X-ray diffraction study were grown by evaporation of the dichloromethane solution of **2C** at -5°C

References:

[1] P. Serp, M. Hernandez, B. Richard, P. Kalck, *Eur. J. Inorg. Chem.* **2001**, *9*, 2327..
